# Supplementary material for: Genetic connectivity of the scalloped hammerhead shark Sphyrna lewini across Indonesia and the Western Indian Ocean
Source: PLoS One. 2020 Oct 1;15(10):e0230763. doi: 10.1371/journal.pone.0230763 (PMC7529310; doi:10.1371/journal.pone.0230763)

PLOS ONE

Genetic connectivity and diversity of endangered species the scalloped hammerhead shark Sphyrna lewini (Griffith & Smith 1834) population in Indonesia and Western Indian Ocean

--Manuscript Draft--

Manuscript Number: PONE-D-20-06557

Article Type: Research Article

Full Title: Genetic connectivity and diversity of endangered species the scalloped hammerhead shark Sphyrna lewini (Griffith & Smith 1834) population in Indonesia and Western Indian Ocean

Short Title: Genetic connectivity and diversity of the scalloped hammerhead shark population

Corresponding Author: Hawis Madduppa
Institut Pertanian Bogor Fakultas Perikanan dan Ilmu Kelautan
Bogor, INDONESIA

Keywords: shark fisheries; population genetics; conservation genetics; coral triangle; genetic connectivity

Abstract: The scalloped hammerhead shark Sphyrna lewini is an endangered species which expected to population declined worldwide including in Indonesia due to overexploited. However, there is a lack of information regarding recent population structure to promote proper management and conservation status in Indonesia. This study aimed to investigate the genetic diversity, population structure and connectivity of S. lewini population in Indonesia from three major sharks landing sites in Aceh (n= 41), Balikpapan (n= 30), Lombok (n= 29), and additional sequences retrieved from West Papua (n= 14) and Western Indian Ocean population (n= 65). Analyses of mitochondrial CO1 gene successfully identified a total of 179 sequences of S. lewini with an average 594 bp nucleotide with 4[illegible]lymorphic loci in 4 haplotypes for Indonesian population and 8 haplotypes f[illegible]Western Indian Ocean. The overall values of genetic diversity in Indonesia was high (Hd= 0.7171; π= 0.0126), with the highest was in Aceh (Hd= 0.6683; π= 0.0198), and the lowest was in Papua (Hd= 0.1429; π= 0.0005), while in Western Indian Ocean the overall value was fairly low (Hd= 0.2322; π= 0.0010). The AMOVA and FST revealed three significant population subdivisions in Indonesia (FST= 0.4415; p < 0.001) with separated population for Aceh and West Papua, and a mixing population between Balikpapan and Lombok (FST= 0.044; p = 0.089), whereas relatively no significant differentiation within population in Western Indian Ocean (FST= -0.0131; p = 0.6011), and significant different level showed by Indonesian population compared with Western Indian Ocean population (FST= 0.7403; p < 0.001). The construction of haplotype network exhibited evidence of gene flow and haplotype sharing between populations. This result indicated a complex and limited connectivity population of S. lewini in Indonesia, and between Western Indian Ocean in regional scale which need co-management action across region.

Order of Authors:
Hawis Madduppa, Dr.
Sutanto Hadi
Noviar Andayani
Effin Muttaqin
Benaya Simeon
Muhammad Ichsan
Beginer Subhan

Opposed Reviewers:

Additional Information:

Question	Response

Powered by Editorial Manager® and ProduXion Manager® from Aries Systems Corporation

**Financial Disclosure**<br><br>Enter a financial disclosure statement that describes the sources of funding for the work included in this submission. Review the submission guidelines for detailed requirements. View published research articles from *PLOS ONE* for specific examples.<br><br>This statement is required for submission and **will appear in the published article** if the submission is accepted. Please make sure it is accurate.<br><br>**Unfunded studies**<br>Enter: *The author(s) received no specific funding for this work.*<br><br>**Funded studies**<br>Enter a statement with the following details:<br>• Initials of the authors who received each award<br>• Grant numbers awarded to each author<br>• The full name of each funder<br>• URL of each funder website<br>• Did the sponsors or funders play any role in the study design, data collection and analysis, decision to publish, or preparation of the manuscript?<br>• **NO** - Include this sentence at the end of your statement: *The funders had no role in study design, data collection and analysis, decision to publish, or preparation of the manuscript.*<br>• **YES** - Specify the role(s) played.<br><br>* typeset	NO - The funders had no role in study design, data collection and analysis, decision to publish, or preparation of the manuscript.
**Competing Interests**<br><br>Use the instructions below to enter a competing interest statement for this submission. On behalf of all authors, disclose any competing interests that could be perceived to bias this work—acknowledging all financial support and any other relevant financial or non-financial competing interests.<br><br>This statement **will appear in the published article** if the submission is accepted. Please make sure it is	The authors have declared that no competing interests exist.

accurate. View published research articles from *PLOS ONE* for specific examples.<br><br>**NO authors have competing interests**<br><br>Enter: *The authors have declared that no competing interests exist.*<br><br>**Authors with competing interests**<br><br>Enter competing interest details beginning with this statement:<br><br>*I have read the journal's policy and the authors of this manuscript have the following competing interests: [insert competing interests here]*<br><br>* typeset	
**Ethics Statement**<br><br>Enter an ethics statement for this submission. This statement is required if the study involved:<br><br>• Human participants<br>• Human specimens or tissue<br>• Vertebrate animals or cephalopods<br>• Vertebrate embryos or tissues<br>• Field research<br><br>Write "N/A" if the submission does not require an ethics statement.<br><br>General guidance is provided below. Consult the submission guidelines for detailed instructions. **Make sure that all information entered here is included in the Methods section of the manuscript.**	All specimens were collected in dead condition, no live specimens were obtained. Therefore, no approval from any institutional animal ethics committee was required. The samples collection and transport were in accordance with the regulation of Ministry of Marine Affairs and Fisheries of the Republic of Indonesia Number 5/PERMEN-KP/2018 on the prohibition of cowboy shark and hammerhead shark export from Indonesia. This study was also approved under permission number 276/BPSPL.03/PRL/X/2018 and 319/PNK/BPSPL.03/PK.230/REKOM/X/2018 by Ministry of Marine Affairs and Fisheries of the Republic of Indonesia.

Powered by Editorial Manager® and ProduXion Manager® from Aries Systems Corporation

**Format for specific study types**<br><br>**Human Subject Research (involving human participants and/or tissue)**<br>• Give the name of the institutional review board or ethics committee that approved the study<br>• Include the approval number and/or a statement indicating approval of this research<br>• Indicate the form of consent obtained (written/oral) or the reason that consent was not obtained (e.g. the data were analyzed anonymously)<br><br>**Animal Research (involving vertebrate animals, embryos or tissues)**<br>• Provide the name of the Institutional Animal Care and Use Committee (IACUC) or other relevant ethics board that reviewed the study protocol, and indicate whether they approved this research or granted a formal waiver of ethical approval<br>• Include an approval number if one was obtained<br>• If the study involved *non-human primates*, add *additional details* about animal welfare and steps taken to ameliorate suffering<br>• If anesthesia, euthanasia, or any kind of animal sacrifice is part of the study, include briefly which substances and/or methods were applied<br><br>**Field Research**<br><br>Include the following details if this study involves the collection of plant, animal, or other materials from a natural setting:<br>• Field permit number<br>• Name of the institution or relevant body that granted permission	
**Data Availability**<br><br>Authors are required to make all data underlying the findings described fully available, without restriction, and from the time of publication. PLOS allows rare exceptions to address legal and ethical concerns. See the PLOS Data Policy and FAQ for detailed information.	Yes - all data are fully available without restriction

Powered by Editorial Manager® and ProduXion Manager® from Aries Systems Corporation

<table>
<tr><td>A Data Availability Statement describing where the data can be found is required at submission. Your answers to this question constitute the Data Availability Statement and will be published in the article, if accepted.

Important: Stating ‘data available on request from the author’ is not sufficient. If your data are only available upon request, select ‘No’ for the first question and explain your exceptional situation in the text box.

Do the authors confirm that all data underlying the findings described in their manuscript are fully available without restriction?</td><td></td></tr>
<tr><td>Describe where the data may be found in full sentences. If you are copying our sample text, replace any instances of XXX with the appropriate details.

• If the data are held or will be held in a public repository, include URLs, accession numbers or DOIs. If this information will only be available after acceptance, indicate this by ticking the box below. For example: All XXX files are available from the XXX database (accession number(s) XXX, XXX.).
• If the data are all contained within the manuscript and/or Supporting Information files, enter the following: All relevant data are within the manuscript and its Supporting Information files.
• If neither of these applies but you are able to provide details of access elsewhere, with or without limitations, please do so. For example:

Data cannot be shared publicly because of [XXX]. Data are available from the XXX Institutional Data Access / Ethics Committee (contact via XXX) for researchers who meet the criteria for access to confidential data.

The data underlying the results presented in the study are available from (include the name of the third party</td><td>All relevant data are within the manuscript and its Supporting Information files.</td></tr>
</table>

and contact information or URL).<br>• This text is appropriate if the data are owned by a third party and authors do not have permission to share the data.<br><br>* typeset	
Additional data availability information:	Tick here if the URLs/accession numbers/DOIs will be available only after acceptance of the manuscript for publication so that we can ensure their inclusion before publication.

Powered by Editorial Manager® and ProduXion Manager® from Aries Systems Corporation

COVER LETTER FOR SUBMISSION OF NEW MANUSCRIPTS

To: Joerg Heber
Editor-in-Chief
PLOS ONE Journal

Subject: **SUBMISSION OF NEW MANUSCRIPT FOR EVALUATION**

I am enclosing herewith a manuscript entitled **"Genetic connectivity and diversity of endangered species the scalloped hammerhead shark *Sphyrna lewini* (Griffith & Smith 1834) population in Indonesia and Western Indian Ocean"** submitted to **"PLOS ONE Journal"** for possible evaluation.

With the submission of this manuscript I would like to undertake that the above-mentioned manuscript has not been published elsewhere, accepted for publication elsewhere or under editorial review for publication elsewhere; and that my institution's **IPB University** representative is fully aware of this submission.

I would also like to share the following information with Editor-in-Chief

Study's contribution to the scientific literature:
This study contributes to the scientific literature particularly in *S. lewini* research and management conducted in Indonesia and the Western Indian Ocean region, where study literatures are still limited.

Relate the study to previously published work:
This study complements to previous study regarding population genetic and connectivity of *S. lewini* in regional and global scale which provides important finding from region (Indonesia and Western Indian Ocean) that poorly evaluated before.

Specify the type of article: **Research article**

Suggest appropriate Academic Editors to handle your manuscript: Prof. Dr. Susana Caballero

Corresponding Author

Dr. Hawis Madduppa

Manuscript
Click here to download Manuscript Hadi et al_SphyrnaLewini.docx

Genetic connectivity and diversity of endangered species the scalloped hammerhead shark *Sphyrna lewini* (Griffith & Smith 1834) population in Indonesia and Western Indian Ocean

Sutanto Hadi[1], Noviar Andayani[2,3], Effin Muttaqin[3], Benaya M Simeon[3], Muhammad Ichsan[3], Beginer Subhan[1] and Hawis Madduppa[1]*

[1] Department of Marine Science and Technology, Faculty of Fisheries and Marine Sciences, IPB University, Indonesia

[2] Department of Biology, Faculty of Mathematic and Natural Science, University of Indonesia, Depok, Indonesia

[3] Wildlife Conservation Society Indonesia, Bogor, Indonesia

*Corresponding author

Email: hawis@apps.ipb.ac.id

Abstract

The scalloped hammerhead shark *Sphyrna lewini* is an endangered species which expected to population declined worldwide including in Indonesia due to overexploited. However, there is a lack of information regarding recent population structure to promote proper management and conservation status in Indonesia. This study aimed to investigate the genetic diversity, population structure and connectivity of *S. lewini* population in Indonesia from three major sharks landing sites in Aceh (n= 41), Balikpapan (n= 30), Lombok (n= 29), and additional sequences retrieved from West Papua (n= 14) and Western Indian Ocean population (n= 65). Analyses of mitochondrial CO1 gene

successfully identified a total of 179 sequences of *S. lewini* with an average 594 bp nucleotide with 40 polymorphic loci in 4 haplotypes for Indonesian population and 8 haplotypes for Western Indian Ocean. The overall values of genetic diversity in Indonesia was high (Hd= 0.7171; π= 0.0126), with the highest was in Aceh (Hd= 0.6683; π= 0.0198), and the lowest was in Papua (Hd= 0.1429; π= 0.0005), while in Western Indian Ocean the overall value was fairly low (Hd= 0.2322; π= 0.0010). The AMOVA and F_{ST} revealed three significant population subdivisions in Indonesia (F_{ST}= 0.4415; $p < 0.001$) with separated population for Aceh and West Papua, and a mixing population between Balikpapan and Lombok (F_{ST}= 0.044; p = 0.089), whereas relatively no significant differentiation within population in Western Indian Ocean (F_{ST}= -0.0131; p = 0.6011), and significant different level showed by Indonesian population compared with Western Indian Ocean population (F_{ST}= 0.7403; $p < 0.001$). The construction of haplotype network exhibited evidence of gene flow and haplotype sharing between populations. This result indicated a complex and limited connectivity population of *S. lewini* in Indonesia, and between Western Indian Ocean in regional scale which need co-management action across region.

Introduction

The scalloped hammerhead shark, *Sphyrna lewini* (*S. lewini*) is semi-oceanic species which distributed circumglobally along tropical waters, abundant both around continental margins and close to the coastal region and islands [1-2]. This species has a unique modification of laterally expanded head which enhanced them with the ability to navigate and follow geomagnetic orientation across the ocean [3-5]. *S. lewini* has a capacity to move in high rates of dispersal. Female *S. lewini* exhibits site fidelity to single nursery

area and indicate no evidence of continuing inter oceanic migration, otherwise male *S. lewini* spread in a long distance spatial across ocean waters with clear evidence in cross reproduction and gametes transmission [6].

S. lewini is one of the threatened species of sharks which estimated one to three million were killed annually worldwide due to fishing and shark fins trade. This species was considered to be an underexploited species in 1999, however, currently listed to be endangered species in the IUCN red list [7] and Appendix II of The Convention on International Trade in Endangered Species (CITES). High exploitation of the population of *S. lewini* may has an impact on the population structure, reducing the fecundity of species and genetic diversity of populations [8]. Furthermore, this species has low resilience to exploitation since late sexual maturity and a long lifetime in nature [9-10].

The study of population genetic have become an important tool for understanding population connectivity, defining fisheries management and conservation strategies [11]. Genetic of population structure of *S. lewini* which important to fisheries stock management has been widely investigated in different coastal area and ocean basin on a global and regional scale [6, 12]. Duncan *et al*. [13] revealed a global phylogeographic study of *S. lewini* which indicated that Indo-West Pacific was a centre of diversity for tropical sharks like *S. lewini* with a high-unique genetic diversity, however, no samples from Indonesia were included, also the study by Ovenden *et al.* [14] which conducted with limited samples from Indonesia.

The aim of this study was to investigate genetic diversity, population structure, and connectivity of *S. lewini* among population in Indonesia where the population are affected by fishing activities, observe connectivity in regional scale in Western Indian Ocean, and also suggest the implication for the species management and conservation.

Material and Methods

Ethics Statement

All specimens were collected in dead condition, no live specimens were obtained. Therefore, no approval from any institutional animal ethics committee was required. The samples collection and transport were in accordance with the regulation of Ministry of Marine Affairs and Fisheries of the Republic of Indonesia Number 5/PERMEN-KP/2018 on the prohibition of cowboy shark and hammerhead shark export from Indonesia. This study was also approved under permission number 276/BPSPL.03/PRL/X/2018 and 319/PNK/BPSPL.03/PK.230/REKOM/X/2018 by Ministry of Marine Affairs and Fisheries of the Republic of Indonesia.

Tissue samples collection

A total of 100 tissue samples of *S. lewini* were collected from October 2017 to November 2018, consisting of 41 samples from Meulaboh and Aceh Jaya fishing port (Aceh), 30 samples from local shark landing in Balikpapan, and 29 samples from Tanjung Luar fishing port (Lombok). The samples were dissected from flesh or fin tissues approximately 0.5 cm^3 preserved in sample bottle contained 96% ethanol.

DNA extraction, amplification and sequencing

Extraction of DNA conducted in Biodiversity and Biosystematics Laboratory, IPB University following the protocol of the gSYNC DNA extraction kit product. A fragment of mitochondrial Cytochrome Oxidase subunit-I gene (COI) was amplified using forward primer fish-BCL 5' TCA ACY AAT CAY AAA GAT ATY GGC AC '3 and reverse primer fish-BCH 5'ACT TCY GGG TGR CCR AAR AAT CA '3 [15-16] (Baldwin *et al.*

2009; Madduppa *et al.* 2016) in a 24 µL reaction mixture consisted of 3 µL DNA template, 12.5 µL MyTaq HS Red Mix, 9 µL ddH_2O, 1.25 µL forward and reverse primer respectively. Mixture product was processed in a polymerase chain reaction (PCR) thermocycler following a modified cycle [17-18] (Prehadi *et al.* 2015; Hadi *et al.* 2019). The step was initiated with pre denaturation at 94 °C for 15 s, followed by 38 cycles of denaturation at 94 °C for 30 sec, annealing at 50 °C for 30 sec, extension at 72 °C for 45 sec, and finished with a final extension at 72 °C for 10 min. The amplicons were visualized by gel electrophoresis agarose 1.5 % smeared with ethidium bromide at 100 V for 20 min. The gel was observed under UV light to determine a band indicating the presence of DNA fragments. Sequence reaction was performed using the BigDye Terminator v3.1 cycle sequencing kit with an optimized protocol of Applied Biosystems 3730 genetic analyser at the 1st BASE, Malaysia.

Data Analysis

Genetic diversity

Sequence of 100 mitochondrial DNA CO1 were edited and aligned using the CrustalW algorithm implemented in MEGA 6.06 [19]. Genetic diversity parameters including the number of haplotypes, haplotype diversity (Hd), and nucleotide diversity (π) were calculated using the DNASp v6 [20] and Arlequin v.3.5 program [21]. We also reanalysed additional COI sequences data of *S. lewini* retrieved from GenBank (Table 1) from West Papua (n= 14) obtained by Sembiring *et al.* [22] and sequences from previous study in India (n= 6) [23], United Arab Emirates (n= 30) [24], and Madagascar (n= 29) [25] to show genetic diversity in Indonesian and Western Indian Ocean.

Table 1. Localities, total number (n), and accession number of COI gene sequences of *S. lewini* in Western Papua (Indonesia), India, United Arab Emirates and Madagascar (Western Indian Ocean) retrieved from GenBank

Localities	N	Accession number
**Indonesia**		
West Papua	14	KF590254-55, KF590271-76, KF793729, KF793738-42
**Western Indian Ocean**		
India	6	KF899746-51
United Arab Emirates	30	KP177238-41, KP177241, KP177254, KP177262, KP177272, KP177285-99, KP177300-07
Madagascar	29	HQ171735-47, HQ171761-76

Population structure

An analysis of molecular variance (AMOVA) and Wright's fixation index (F_{ST}) [26] were conducted for three major group: 1) within and among four population in Indonesia, 2) within and among three population in Western Indian Ocean, and 3) comparation between population from Indonesia and Western Indian Ocean using the Arlequin v.3.5 program (setting up 1000 permutations, α= 0.05 for significance level threshold) to estimate overall extent of genetic variation and differentiation level in Indonesia and Western Indian Ocean. Population differentiation and its significance between sampling sites were also calculated with pairwise estimates. The value of F_{ST} is a range of zero to one, low level of differentiation was implied from the closer of F_{ST} value to zero, and otherwise the high level will be closer to one [27-28].

Genetic connectivity

A haplotype network was constructed with a median-joining method in the Network v5.1.1.0 program [29] for all haplotypes found in Indonesia and Western Indian Ocean to obtain

haplotype connectivity in broader spatial connection in the regional area in the Indian Ocean. Distribution of haplotypes for each location was also provided in a proper map to show clear distribution of haplotypes and genetic connectivity among populations.

Results

Genetic Diversity

A total of 179 sequences of the 594 bp mitochondria CO1 gene of *S. lewini* obtained from three sampling sites (Aceh, Balikpapan, Lombok), and additional sequences of West Papua and West Indian Ocean region were generated a total of 11 haplotypes variation with 40 polymorphic loci (Table 2). All the sequences of *S. lewini* obtained from this study were deposited in BOLD System with Barcode Index Number (BIN) registry of BOLD:AAA2403.

Comparison of genetic diversity of *S. lewini* based on haplotype and nucleotide diversity was varied in value (Table 3). The haplotype diversity (Hd) among localities in Indonesia was ranged from 0.1429-0.6683 and nucleotide diversity (π) was ranged from 0.005-0.0198. The highest value of genetic diversity in Indonesia was showed by population in Aceh (Hd= 0.6683; π= 0.0198), followed by Balikpapan population which lower in haplotype and nucleotide diversity (Hd= 0.6460; π= 0.0020), whereas the lowest was West Papua (Hd= 0.1429, π= 0.0005). Population of *S. lewini* in Lombok also had a fairly low genetic diversity value (Hd= 0.3621; π= 0.0010). However, overall genetic diversity in Indonesia relatively high in value (Hd= 0.7171; π= 0.0126). On the other hand, the value of genetic diversity of Western Indian Ocean was low in average with ranged of 0.000-0.4667. The overall diversity in Western Ocean also low in value (Hd= 0.2322; π= 0.0010).

Table 2. Forty polymorphic loci of 11 haplotypes from 179 sequences of the mitochondrial COI gene of *S. lewini* from four localities in Indonesia and three localities in Western Indian Ocean

Haplotypes	Locus Position																																						
	**1**	**15**	**24**	**27**	**33**	**45**	**48**	**63**	**69**	**105**	**120**	**138**	**141**	**156**	**165**	**174**	**186**	**198**	**229**	**279**	**289**	**303**	**306**	**325**	**339**	**342**	**346**	**366**	**396**	**420**	**453**	**456**	**459**	**483**	**495**	**498**	**522**	**531**	**534**	**543**
H1	A	T	A	C	T	T	G	T	T	A	T	C	G	T	T	C	C	T	T	C	G	T	T	C	C	A	C	T	T	C	C	T	T	C	T	C	C	T	C	T
H2	.	.	.	A	.	.	.	.	.	G	.	A	T	.	C	T	T	C	C	T	.	C	C	T	T	C	.	C	C	T	.	.	.	A	.	T	.	C	.	C
H3	.	.	.	A	.	.	.	.	C	G	.	A	T	.	C	T	T	C	C	T	.	C	.	T	T	C	.	C	C	T	.	.	.	A	.	T	.	C	.	.
H4	.	.	.	A	.	.	.	.	.	G	.	A	T	.	C	T	T	C	C	T	.	C	C	T	T	C	.	C	C	T	.	.	.	A	.	T	.	C	.	.
H5	.	.	.	.	.	.	.	.	.	.	.	.	.	.	.	.	.	.	.	.	.	.	.	.	.	.	.	.	.	.	.	.	G	.	G	.	.	.	.	.
H6	.	.	.	.	.	C	.	.	.	.	.	.	.	.	.	.	.	.	.	.	.	.	.	.	.	.	.	.	.	.	.	.	.	.	.	.	.	.	.	.
H7	.	.	C	.	.	.	.	.	.	.	.	.	.	G	.	.	.	.	.	.	.	.	.	.	.	.	.	.	.	.	.	.	.	.	.	.	.	.	.	.
H8	.	C	.	.	C	.	.	.	.	.	.	.	.	.	.	.	.	.	.	.	.	.	.	.	.	.	.	.	.	.	.	.	.	.	.	.	.	.	.	.
H9	.	.	.	.	.	.	.	.	.	.	G	.	.	.	.	.	.	G	.		T	.	.	.	.	.	.	.	.	.	.	.	.	.	.	.	.	.	G	.
H10	G	.	.	.	.	.	.	G	.	.	.	.	.	G	.	.	.	.	.	.	.	.	.	.	.	.	.	.	.	.	.	.	.	.	.	.	.	.	.	.
H11	.	.	.	.	.	.	.	.	.	.	.	.	.	.	.	.	.	.	.	.	.	.	.	.	.	.	T	.	.	.	T	.	.	.	C	.	T	.	.	.

*Nucleobase at each position is given for H1 and only nucleobase different from H1 are written for all other haplotypes. Identical nucleobase to H1 are indicated with periods (.)

Table 3. Genetic diversity of *S. lewini* based on sample size (n), haplotype number (Hn), haplotype diversity (Hd), and nucleotide diversity (π) from each population sites in Indonesia and Western Indian Ocean region

Population	n	Genetic Diversity		
		**Hn**	**Hd**	**Π**
**Indonesia**				
Aceh (ACH)	41	4	0.6683	0.0198
Balikpapan (BPN)	30	3	0.6460	0.0020
Lombok (LOM)	29	3	0.3621	0.0010
West Papua (WEP)	14	2	0.1429	0.0005
Overall Indonesia	114	4	0.7171	0.0126
**Western Indian Ocean**				
India (IND)	6	1	0.0000	0.0000
United Arab Emirates (UAE)	30	8	0.4667	0.0022
Madagascar (MDG)	29	1	0.0000	0.0000
Overall Western Indian Ocean	65	8	0.2322	0.0010

Population structure

The analysis of fixation index (F_{ST}) and significant P-value between and within four *S. lewini* populations (ACH, BPN, LOM, and WEP) in Indonesia and three population (IND, UAE, and MDG) were showed in Table 4. The overall value of F_{ST} in Indonesia was high and significant (F_{ST}= 0.442; P-value= 0.000) which indicated that there were multiple subdivition in Indonesia. Low F_{ST} value and no significant different was exhibited by population from Western Indian Ocean (F_{ST}= -0.0131; P-value= 0.6011), while comparative population structure between Indonesia and Western Indian Ocean showed significant differentiation (F_{ST}= 0.7403; P-value= 0.0000). The pairwise F_{ST} between population in four location in Indonesia and comparison with Western Indian Ocean population was showed in Table 5. The overall pairwise analysis by distance method indicated significant differentiation among four population in Indonesia, except between BPN and LOM which showed fairly low F_{ST} value and no significant P-value.

Furthermore, among population in Indonesia ACH population was tent to closer to WIO population with lower F_{ST} value rather than F_{ST} range value of other localities in Indonesia to Western Indian Ocean.

Table 4. Analysis of molecular variance (AMOVA) for percentage of variation (%), F_{ST} value and significant level (P-value) of *S. lewini* for Indonesian population, Western Indian Ocean population and between Indonesian and Western Indian Ocean population

Source of variation	df	Percentage of variation (%)	F_{ST} value	P-value
**Indonesia**				
Among Population	3	44.15	0.4415	0.000
Within Population	110	55.85		
Total	113			
**Western Indian Ocean**				
Among Population	2	-1.31	-0.0131	0.6011
Within Population	62	101.31		
Total	64			
**Indonesia vs Western Indian Ocean**				
Among Population	1	74.04	0.7403	0.0000
Within Population	177	25.96		
Total	178			

Table 5. Pairwise F_{ST} value (below diagonal) and P-value (above diagonal) between *S. lewini* population in Aceh (ACH), Balikpapan (BPN), Lombok (LOM), West Papua (WEP), and Western Indian Ocean (WIO)

Sample Sites	ACH	BPN	LOM	WEP	WIO
**ACH**	-	0.0000	0.0000	0.0000	0.0000
**BPN**	0.4271	-	0.0898	0.0010	0.0000
**LOM**	0.4377	0.0435	-	0.0000	0.0000
**WEP**	0.3981	0.4945	0.7355	-	0.0000
**WIO**	0.5094	0.9647	0.9731	0.9750	-

Genetic Connectivity

There were two main group of haplotypes that show by haplotype network analysis (Fig 1), hereafter referred to as Clade A and Clade B. Clade A consisted of H1 found in multiple region in Aceh, India, United Arab Emirates, and Madagascar, while H5, H6, H7, H8, H9, H10, H11 were originated only from United Arab Emirates. Clade B consisted of three haplotypes (H2, H3, H4) which spread evenly in Indonesia, though there were predominant haplotype found in every single region. Aceh, Balikpapan, Lombok, and West Papua was predominated by H1, H2, H4, and H3 respectively (Fig 2).

Fig 1. Haplotype network of *S. lewini* (n= 179) population in Indonesia and Western Indian Ocean region, constructed with Median Joining method

Fig 2. Distribution of 11 haplotypes of *S. lewini* population from Indonesia and Western Indian Ocean in regional scale

Discussion

Genetic diversity

The overall genetic diversity both at the haplotype and nucleotide level of *S. lewini* was relatively high for population in Indonesia. This finding is consistent with the result of Ovenden *et al.* [14] with mitochondria control region of *S. lewini* from three localities in Indonesia including Lombok and high genetic diversity of *S. lewini* in global scale [13]. The scalloped hammerhead sharks are high migratory species with a wide distribution in tropical and warm-temperate waters and possibly move across oceanic waters up to 1500 km far [9]. In principle, with migration capability and broad ecological niches, this species tends to have higher genetic diversity than narrow ecological niches species [30].

High genetic diversity values are commonly related to large population size [31], also promotes by some factors such local population sizes, fast generations times [32], high nucleotide substitution rate [33], and the high gene flow between geographical distant populations.

The finding of relatively high genetic diversity of *S. lewini* in Indonesia appear inconsistent with general assumption that overexploitation as both target fishing and bycatch have declined the scalloped hammerhead shark worldwide population [34], however, this could be relevant with the fact occurred in Lombok. Lower genetic diversity evidence occurred in Lombok probably driven by continuous fishing pressure. *S. lewini* were the top three targeted sharks landed in Tanjung Luar fishing port, Lombok and have faced high fishing pressure more than 40 years that lead to overfishing with exploitation rate (E) reached 0.59 recently [35-36].

Furthermore, according to fisheries global information system statistic of FAO [37] the hammerhead sharks (Sphyrnidae) including *S. lewini* are one of the important sharks which highly exploited in the last two decades which estimated rapidly increase in global capture since 220 ton per year in 1985 reaching approximately 10,362 tons per year in 2016. In the same period the capture level in Indonesia also significantly increase reaching approximately 1,492 tons in 2016 with the fact that Indonesia was in the highest volume of shark and rays caught on the global catches reported in 2000-2011 [38].

Clarke *et al*. [39] was reported similar finding regarding global mitochondrial DNA of silky shark, *Carcharhinus falciformis* with high genetic diversity under circumstance of overexploitation. Elasmobranch possible to have adaptability with environmental and anthropogenic stress which causes genetic bottlenecks because of their particular life histories [40]. Moreover, population decline caused by fishery activities recently might

be insufficient in time to reduce genetic diversity particularly for long life species (13–20 years) including S. lewini [41-42].

However, indeed declining large population continuously, as was expected occur with *S. lewini* population in Lombok, had positive correlation with loss of genetic diversity and driven a population bottleneck [42-43] as revealed by Pinsky and Palumbi [44] in some marine fish meta-analysis.

Population structure

Our finding indicated homogeneity among *S. lewini* population from Balikpapan and Lombok. Pairwise F_{ST} analysis detected no significant genetic differentiation in these two contiguous spatial distant. This finding complements with previous studies conducted in Indo-Australia waters. Ovenden *et al.* [14] reported similar evidence for mitochondrial control region on population structure with no differentiation for two population of *S. lewini* between Indonesian population including Lombok and northern Australian. This pattern of single stock population seems to suggest that these localities are migration zone of *S. lewini* and reproductive movement might occur in coastal area of these locations though there are strong Indonesian throughflow current between Kalimantan and Sulawesi island. Adults *S. lewini* are highly migratory with large body size that supports of high dispersal capacity of this species in hence possibly to control that geographical barrier. Otherwise, we observed different result with comparison among these two population in central Indonesia with population in Aceh (western Indonesia) and West Papua (eastern Indonesia) which exhibited strong genetic differentiation. These regions were spatially separated by long distance with the possibility of more complex barriers

both inter island barrier and anthropogenic factors of high commercial and artisanal fishing pressure such along southern and northern Java.

Genetic connectivity

We found an interesting finding regarding haplotype sharing between population in Indonesia and Indian Ocean population. H1 was the unique haplotype found in Aceh, Indonesia and this haplotype was correlated with the most common haplotype in three locations of western Indian Ocean region from India, United Arab Emirates, and Madagascar which could indicated that there was connectivity between population from Aceh and western Indian Ocean. Schott and McCreary [45] described Indian Ocean seasonal current carried water from north Sumatera through Arabian Sea to Madagascar which was possible as connectivity channel of two regions with thus allowing *S. lewini* to migrate and lead to cross breeding. Similar predominant haplotype (H1) of *S. lewini* from Aceh with population from India, United Arab Emirates, and Madagascar were evidence of genetic sharing process between population in Indian Ocean region. However, the *S. lewini* population from Balikpapan, Lombok and West Papua was indicated isolation from population in western Indian Ocean and shared haplotype network only in eastern Indonesia water.

Conservation implication

The overall high value of genetic diversity of *S. lewini* population in Indonesia suggests this species has not experienced a genetic loss and bottleneck to face of exploitation pressure. However, lower genetic diversity of *S. lewini* in Lombok and West Papua indicated a higher risk of genetic diversity loss that probably as the result of high fisheries

pressure. Our genetic assessment of *S. lewini* from four localities which revealed a single stock exist between Lombok and Balikpapan, and separated stock for Aceh and West Papua indicating that management of this species should occur on stock-based approach at least on three mitochondrial-stock conservation management unit. A complex and limited connectivity population of *S. lewini* that existed in population in Indonesia, and between Western Indian Ocean indicating the importance to promote collaborative management strategic between Indonesian management agencies, and also with West Indian Ocean agencies in regional scales.

Conclusion

This study has shown important findings regarding population structure of *S. lewini* in Indonesia with high genetic diversity and three significant subdivision that indicated capability of population to adapt to rapid environmental change and pressure including fishing activities. However, lower genetic value in Lombok and West Papua should still be considered. Genetic sharing which detected between population in Indonesia and Western Indian Ocean proved connectivity between population in Indonesia and also connecting with Western Indian Ocean population. Collaborative action across region is needed to promote sustainable management and conservation purpose both in Indonesia and Western Indian Ocean in regional scale.

Acknowledgement

This research was supported by Wildlife Conservation Society (WCS)-Indonesia, in collaboration with Faculty of Fisheries and Marine Sciences IPB University. Sutanto Hadi received a scholarship from Indonesia Endowment Fund for Education (LPDP). We also

appreciated to BPSPL Satker Balikpapan, Head of PPI Manggar Balikpapan, and also Marine Biodiversity and Biosystematic Lab (BIODIVSI) fellows for their help during research, and to reviewers who have provided highly constructive suggestions for better writing of the paper.

References

1. Klimley AP, Butler SB. Immigration and emigration of a pelagic fish assemblage to seamounts in the Gulf of California related to water mass movements using satellite imagery. Mar Ecol Prog Ser. 1988;49: 11-20.
2. Compagno LJV. FAO Species Catalogue: Sharks of the world. Rome: Food And Agriculture Organization;1984.
3. Montgomery JC, Walker MM. Orientation and navigation in elasmobranchs: which way forward? Environ Biol Fishes. 2001;60: 109-116.
4. Kajiura SM, Holland KN. Electroreception in juvenile scalloped hammerhead and sandbar sharks. J Exp Biol. 2002;205: 3609-3621.
5. Meyer CL, Holland KN, Papastamatiou YP. Sharks can detect changes in the geomagnetic field. J R Soc Interface. 2005;2: 129-130.
6. Daly-Engel TS, Seraphin KD, Holland KN, Coffey JP, Nance HA, Toonen RJ, et al. Global Phylogeography with Mixed-Marker Analysis Reveals Male-Mediated Dispersal in the Endangered Scalloped Hammerhead Shark (*Sphyrna lewini*). PLoS ONE. 2012;7(1): e29986. doi: 10.1371/journal.pone.0029986.
7. Castillo-olguin E, Aribe-Alcocer M, Diaz-Jaimes P. Assessment of the population genetic structure of Sphyrna lewini to identify conservation units in the Mexican Pacific. Ciencias Marinas. 2012;38(4): 635-652. doi: 10.7773/cm.v38i4.2110.

8. Ward RD. Genetics in fisheries management. Hidrobiologia. 2000;420: 191–201.
9. Kohler NE, Turner PA. Shark tagging: a review of conventional methods and studies. Environ Biol Fishes. 2001;60: 191-223.
10. Bessudo S, Soler GA, Kimley PA, Ketchum J, Arauz R, Hearn A, et al. Vertical and horizontal movements of scalloped hamemerhead shark (*Sphyrna lewini*) around Malpelo and Cocos islands (Tropical Eastern Pacific) using satellite telemetry. Boletín de Investigaciones Marinas y Costeras. 2011;40: 91-106.
11. Cardeñosa D, Hyde J, Caballero S. Genetic Diversity and Population Structure of the Pelagic Thresher Shark (*Alopias pelagicus*) in the Pacific Ocean: Evidence for Two Evolutionarily Significant Units. PLoS ONE. 2014;9(10): e110193. doi: 10.1371/journal.pone.0110193.
12. Quintanilla S, Gómez A, Mariño-Ramírez C, Sorzano C, Bessudo S, Soler G, et al. Conservation Genetics of the Scalloped Hammerhead Shark in the Pacific Coast of Colombia. J Hered. 2015;106: 448-458. doi:10.1093/jhered/esv050.
13. Duncan KM, Martin AP, Bowen BW, De Coute HG. Global phylogeography of the scalloped hammerhead shark (Sphyrna lewini). Mol Ecol. 2006;15:2239-51.
14. Ovenden JR, Kashiwagi T, Broderick D, Giles J, Salini J. The extent of population genetic subdivision differs among four co-distributed shark species in the Indo-Australian archipelago. BMC Evol Biol. 2009;9: 40. doi:10.1186/1471-2148-9-40.
15. Baldwin CC, Mounts JH, Smith DG, Weigt LA. Genetic identification and color descriptions of early life-history stages of Belizean *Phaeoptyx* and *Astrapogon* (Teleostei: Apogonidae) with comments on identification of adult *Phaeoptyx*. Zootaxa. 2009;2008: 1-22.

16. Madduppa H, Ayuningtyas RU, Subhan B, Arafat D. Exploited but unevaluated: DNA barcoding reveals skates and stingrays (Chordata, Chondrichthyes) species landed in the Indonesian fish market. IJMS. 2016;21: 77-84.
17. Prehadi, Sembiring A, Kurniasih EM, Rahmad, Arafat D, Subhan B et al. DNA barcoding and phylogenetic reconstruction of shark species landed in Muncar fisheries landing site in comparison with Southern Java fishing port. Biodiversitas. 2015;16: 55-61.
18. Hadi S, Anggraini NP, Muttaqin E, Simeon BM, Subhan B, Madduppa H. Genetic diversity of the endangered species Sphyrna lewini (Griffith and Smith 1834) in Lombok based on mitochondrial DNA. IOP Conf. Ser.: Earth Environ. Sci. 2019;236: 012024.
19. Tamura K, Stecher G, Peterson D, Filipski A, Kumar S. Molecular Evolutionary Genetics Analysis Version 6.0. Mol Biol Evol. 2013;30(12): 2725-2729. doi: 10.1093/molbev/mst197.
20. Rozas J, Ferrer-Mata A, Sánchez-DelBarriol JC, Guirao-Rico S, LibradoP, Ramos-Onsins SE. DnaSP v6: DNA Sequence Polymorphism Analysis of Large Datasets. Mol Biol Evol. 2017;34: 3299-3302.
21. Excoffier L, H.E. L. Lischer. Arlequin suite ver 3.5: A new series of programs to perform population genetics analyses under Linux and Windows. Mol Ecol Resour. 2010;10: 564-567.
22. Sembiring A, Pertiwi NPD, Mahardini A, Wulandari R, Kurniasih EM, Kuncoro AW, et al. DNA barcoding reveals targeted fisheries for endangered sharks in Indonesia. Fisheries Research. 2015;164: 130-134. doi: 10.1016/j.fishres.2014.11.003.

23. Bineesh KK, Gopalakrishnan A, Akhilesh KV, Sajeela KA, Abdussamad EM, Pillai NGK, et al. DNA barcoding reveals species composition of sharks and rays in the Indian commercial fishery. Mitochondrial DNA. 2016. doi: 10.3109/19401736.2015.1137900.
24. Jabado RW, Ghais SMA, Hamza W, Henderson AC, Spaet JLY, Shivji MS, et al. The trade in sharks and their products in the United Arab Emirates. Biol Conserv. 2015;181: 190-198. doi: 10.1016/j.biocon.2014.10.032.
25. Doukakis P, Hanner R, Shivji M, Bartholomew C, Chapman D, Wong E, et al. Applying Genetic Techniques to Study Remote Shark Fisheries in Northeastern Madagascar. Mitochondrial DNA. 2011;22(S1): 15-20.
26. Wright S. The genetical structure of populations. Annals of Eugenics. 1951;15: 323-354.
27. Wright S. Evolution and the Genetics of Populations. 4th vol. Variability within and among Natural Populations. Chicago: University of Chicago Press; 1978.
28. Hartl DL, Clark AG. Principles of Population Genetics. 3nd ed. Sunderland: Sinauer Associates Inc; 1997.
29. Bandelt H-J, Forster P, Röhl A. Median-joining networks for inferring intraspecific phylogenies. Mol Biol Evol. 1999;16: 37-48.
30. Habel JC, Schmitt T. Vanishing of the common species: Empty habitats and the role of genetic diversity. Biol. Conserv. 1996;218: 211-216. doi: 10.1016/j.biocon.2017.12.018.
31. Frankham R. Relationship of Genetic Variation to Population Size in Wildlife. Conserv. Biol. 1996;10(6): 1500-1508. doi:10.1046/j.1523-1739.1996.10061500.x.

32. Hague MTJ, Routman EJ. Does population size affect genetic diversity? A test with sympatric lizard species. Heredity. 2016;116: 92-98. doi: 10.1038/hdy.2015.76.
33. Martin AP, Naylor GJP, Palumbi SR. Rates of mitochondrial DNA evolution in sharks are slow compared with mammals. Nature. 1992;357: 153-157. doi: 10.1038/357153a0.
34. Compagno LJV, Dando M, Fowler SL. Sharks of the World. Princeton: Princeton University Press; 2005.
35. Hordyk A, Ono K, Valencia S, Loneragan N, Prince J. A novel length-based empirical estimation method of spawning potential ratio (SPR), and tests of its performance, for small-scale, data-poor fisheries. ICES J Mar Sci. 2015;2-15. doi: 10.1093/icesjms/fsu004.
36. Simeon BM, Agustina S, Muttaqin E, Yulianto I, Ichsan M, Muhsin M. Laporan teknis: Pemanfaatan Hasil Tangkap Hiu dan Pari di Tanjung Luar, Provinsi Nusa Tenggara Barat. Bogor: Wildlife Conservation Society-Indonesia Program; 2017.
37. Food and Agriculture Organization (FAO). Fisheries and Aquaculture Information and Statistics Service: Global Capture Production 1950-2017; 2017 [cited 2020 Mar 5]. Database: Indonesian hammerhead shark capture [Internet]. http://www.fao.org/fishery/statistics/global-capture-production/query/es.
38. Dent F, Clarke. State of the Global Market for Shark Products. Rome: Food And Agriculture Organization; 2015.
39. Clarke CR, Karl SA, Horn RL, Bernard AM, Lea JS, Hazin FH et al. Global mitochondrial DNA phylogeography and population structure of the silky shark, *Carcharhinus falciformis.* Mar Biol. 2015;162(5): 945-955. doi: 10.1007/s00227-015-2636-6

40. Chapman DD, Simpfendorfer CA, Wiley TR, Poulakis GR, Curtis C, Tringali M et al. Genetic diversity despite population collapse in a critically endangered marine fish: the smalltooth sawfish (*Pristis pectinata*). J Hered. 2011;102: 643-652.
41. Sánchez-de Ita JA, Quiñónez-Velázquez C, Galván-Magaña F, Bocanegra-Castillo N, Félix-Uraga R. Age and growth of the silky shark *Carcharhinus falciformis* from the west coast of Baja California Sur, Mexico. J App Ichthyol. 2011;27: 20-24.
42. Nei M, Maruyama T, Chakraborty R. The Bottleneck Effect and Genetic Variability in Populations. Evolution. 1975;29(1): 1. doi:10.2307/2407137.
43. Beebee T, Rowe G. An Introduction to Molecular Ecology. 2nd ed. New York: Oxford University Press Inc; 2008.
44. Pinsky ML, Palumbi SR. Meta-analysis reveals lower genetic diversity in overfished populations. Mol Ecol. 2013;23(1): 29-39. doi:10.1111/mec.12509.
45. Schott FA, McCreary JP. The monsoon circulation of the Indian Ocean. Prog Oceanogr. 2001;51: 1-123. doi: 10.1016/S0079-6611(01)00083-0.

Figure 1 Click here to download Figure Fig1_Hadi et al.tif

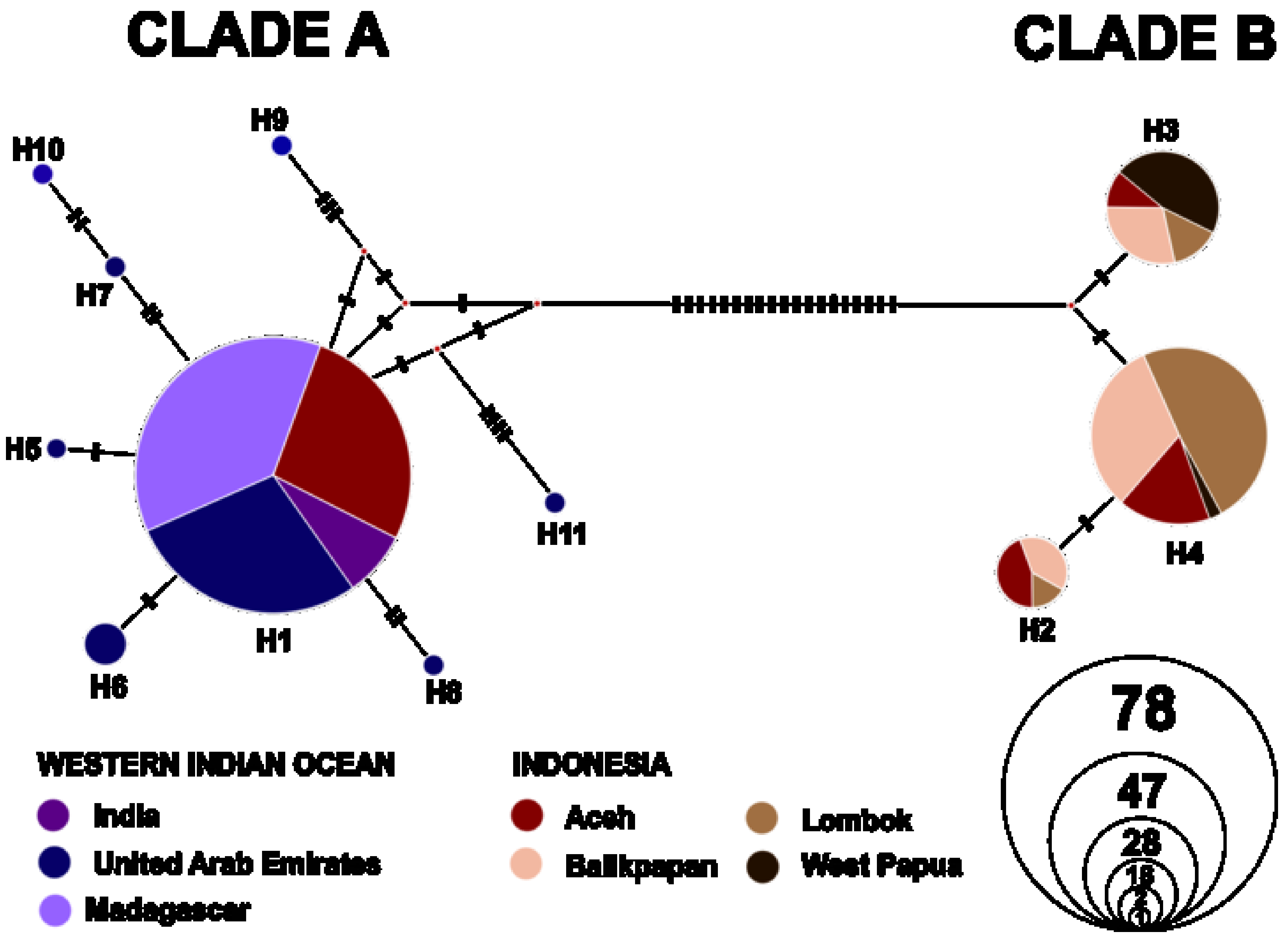

Figure 2

Click here to download Figure Fig2_Hadi et al.tif

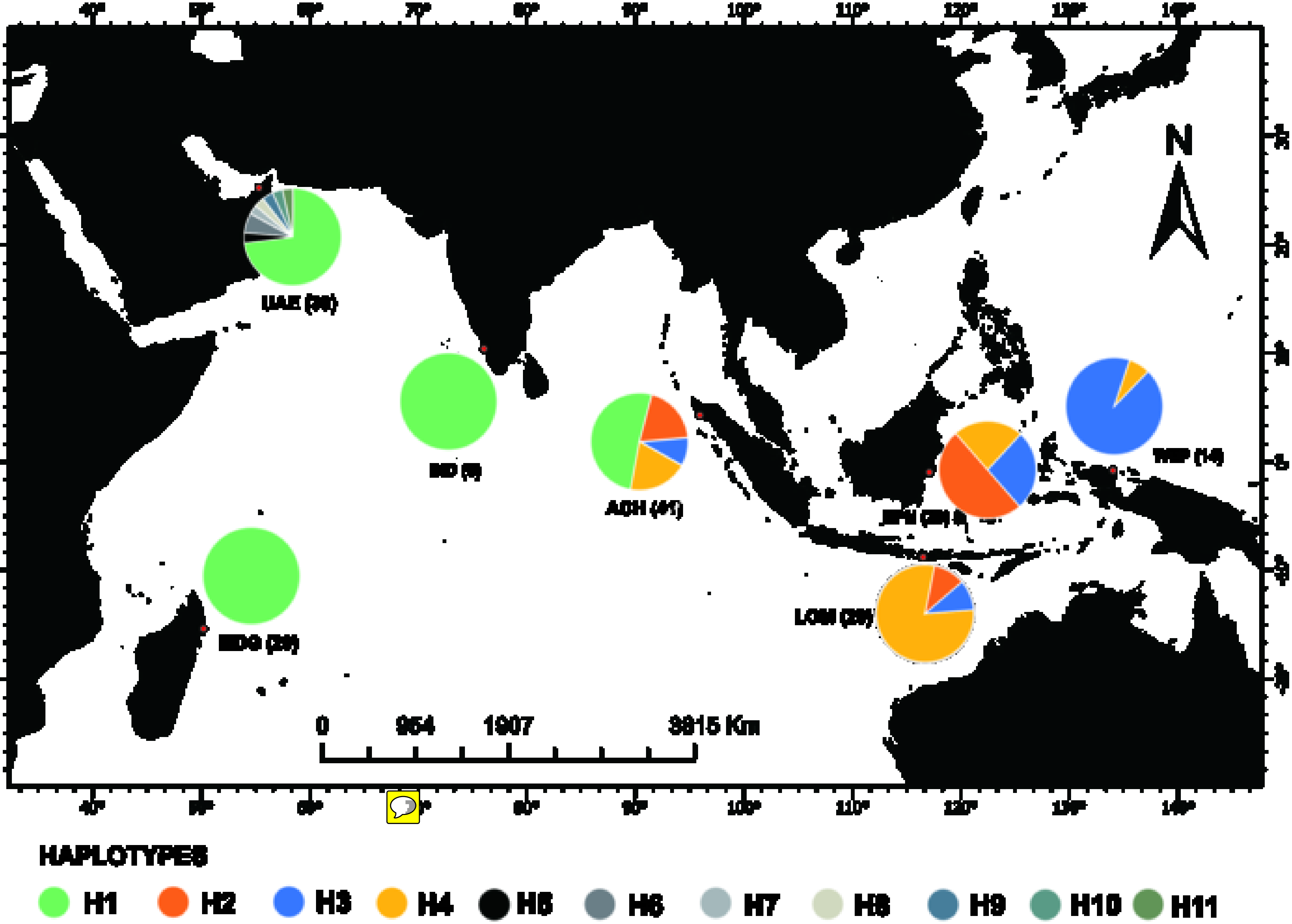

Supplement: Attachment — Submitted filename: PONE-D-20-06557REVed.pdf [file pone.0230763.s001.pdf]
