# Supplementary material for: Genetic connectivity of the scalloped hammerhead shark Sphyrna lewini across Indonesia and the Western Indian Ocean
Source: PLoS One. 2020 Oct 1;15(10):e0230763. doi: 10.1371/journal.pone.0230763 (PMC7529310; doi:10.1371/journal.pone.0230763)
Supplement: Attachment — Submitted filename: PONE-D-20-06557_R2SCcomm.pdf [file pone.0230763.s004.pdf]

PLOS ONE

Genetic connectivity of the scalloped hammerhead shark Sphyrna lewini (Griffith & Smith 1834) population across Indonesia and the Western Indian Ocean

--Manuscript Draft--

**Manuscript Number:**	PONE-D-20-06557R2
**Article Type:**	Research Article
**Full Title:**	Genetic connectivity of the scalloped hammerhead shark Sphyrna lewini (Griffith & Smith 1834) population across Indonesia and the Western Indian Ocean
**Short Title:**	Genetic connectivity of the scalloped hammerhead shark population
**Corresponding Author:**	Hawis Madduppa<br>Institut Pertanian Bogor<br>Bogor, INDONESIA
**Keywords:**	shark fisheries; population genetics; conservation genetics; coral triangle; genetic connectivity
**Abstract:**	Sphyrna lewini is an endangered species and its populations have been declining worldwide, including in Indonesia, because of overexploitation. However, there is a lack of recent information regarding recent the population structure in this species, to promote its proper management and conservation status in Indonesia. This study aimed to investigate the genetic diversity, population structure, and connectivity of the S. lewini population in Indonesia, i.e., in three major shark landing sites: Aceh (n = 41), Balikpapan (n = 30), and Lombok (n = 29); moreover, additional sequences were retrieved from West Papua (n = 14) and Western Indian Ocean (n = 65) populations. Analyses of the mitochondrial CO1 gene successfully identified a total of 179 sequences of S. lewini, with an average size of 594 bp, and 40 polymorphic loci in four haplotypes for the Indonesian population and in eight haplotypes for the Western Indian Ocean population. The overall values of genetic diversity in Indonesia were high (h = 0.717; π = 0.013), with the highest values recorded in Aceh (h = 0.668; π = 0.002) and the lowest detected in Papua (h = 0.143; π = 0.000), whereas the overall value was fairly low in the Western Indian Ocean (h = 0.232; π = 0.001). AMOVA and FST revealed three significant population subdivisions in Indonesia (FST = 0.442; P < 0.001), with separated populations for Aceh and West Papua, and a mixed population between Balikpapan and Lombok (FST = 0.044; P = 0.089); in contrast, a relatively insignificant differentiation was observed within the population of the Western Indian Ocean (FST = –0.013; P = 0.601) and a significant different was detected between the Indonesian and the Western Indian Ocean populations (FST = 0.740; IP < 0.001). The construction of A haplotype network provided evidence of gene flow and haplotype sharing between populations. This study indicated the presence of a complex population of S. lewini with limited connectivity in Indonesia, and in the Western Indian Ocean (at the regional scale), which will require co-management actions across regions.
**Order of Authors:**	Hawis Madduppa, Dr.
	Sutanto Hadi
	Noviar Andayani
	Effin Muttaqin
	Benaya Simeon
	Muhammad Ichsan
	Beginer Subhan
**Opposed Reviewers:**	
**Response to Reviewers:**	Dear Susana Caballero, PhD<br>Academic Editor<br>PLOS ONE

Powered by Editorial Manager® and ProduXion Manager® from Aries Systems Corporation

Thank you for reviewing and suggesting our manuscript:

“Genetic connectivity and diversity of endangered species the scalloped hammerhead shark Sphyrna lewini (Griffith & Smith 1834) population across Indonesia and the Western Indian Ocean”

I would like to response your comments:

Additional Editor Comments (if provided):

This is a very interesting manuscript and it present very important information that will help understanding the genetic diversity of scalloped hammerhead in this region and this information will be likely used for future assignment comparisons for example for market surveys etc. However, the English NEEDS to be improved. I really hope the authors can find the help of a native English speaker that can help them with this. I wrote some things in the PDF I am attaching but it definitely needs an overall improvement of the English
.
Thank you for your interest and suggestion. We have improved overall of the English for better manuscript after proof read by professional native English speaker.

The resolution of the figures needs to be improved because they look grainy.

Thank you for your suggestion. We have improved the resolution of figures and generated in PACE to meet PLOS ONE requirement.

We would appreciate receiving your revised manuscript by 20th of April. When you are ready to submit your revision, log on to https://www.editorialmanager.com/pone/ and select the 'Submissions Needing Revision' folder to locate your manuscript file.
If you would like to make changes to your financial disclosure, please include your updated statement in your cover letter.

To enhance the reproducibility of your results, we recommend that if applicable you deposit your laboratory protocols in protocols.io, where a protocol can be assigned its own identifier (DOI) such that it can be cited independently in the future. For instructions see: ttp://journals.plos.org/plosone/s/submission-guidelines#loc laboratory-protocols

Please include the following items when submitting your revised manuscript:
•A rebuttal letter that responds to each point raised by the academic editor and reviewer(s). This letter should be uploaded as separate file and labelled 'Response to Reviewers'.
•A marked-up copy of your manuscript that highlights changes made to the original version. This file should be uploaded as separate file and labelled 'Revised Manuscript with Track Changes'.
•An unmarked version of your revised paper without tracked changes. This file should be uploaded as separate file and labelled 'Manuscript'.

Journal Requirements:

When submitting your revision, we need you to address these additional requirements:
1.Please ensure that your manuscript meets PLOS ONE's style requirements, including those for file naming. The PLOS ONE style templates can be found at http://www.plosone.org/attachments/PLOSOne_formatting_sample_main_body.pdf and http://www.plosone.org/attachments/PLOSOne_formatting_sample_title_authors_affiliations.pdf

Thank you for your suggestion. We have read and followed the format as The PLOS ONE style templates.

2.In your Methods section, please provide additional location information of the sampling collection sites, including geographic coordinates for the data set if available.

Thank you for your suggestion. We have provided additional location information including geographic coordinates in a table in manuscript

3.We suggest you thoroughly copyedit your manuscript for language usage, spelling, and grammar. If you do not know anyone who can help you do this, you may wish to consider employing a professional scientific editing service.

Thank you for your suggestion. We have sent and proof read our manuscript to a professional scientific editor to check and enhance our manuscript grammar, spelling and language use.

Upon resubmission, please provide the following:
•The name of the colleague or the details of the professional service that edited your manuscript
•A copy of your manuscript showing your changes by either highlighting them or using track changes (uploaded as a *supporting information* file)
•A clean copy of the edited manuscript (uploaded as the new *manuscript* file)

4.Thank you for stating the following in the Acknowledgments Section of your manuscript:
"This research was supported by Wildlife Conservation Society (WCS)-Indonesia, in collaboration with Faculty of Fisheries and Marine Sciences IPB University. Sutanto Hadi received a scholarship from Indonesia Endowment Fund for Education (LPDP)."

We note that you have provided funding information that is not currently declared in your Funding Statement. However, funding information should not appear in the Acknowledgments section or other areas of your manuscript. We will only publish funding information present in the Funding Statement section of the online submission form.

Please remove any funding-related text from the manuscript and let us know how you would like to update your Funding Statement. Currently, your Funding Statement reads as follows:
"NO - The funders had no role in study design, data collection and analysis, decision to publish, or preparation of the manuscript."

Thank you for your suggestion. We have removed any funding-related statement from the
manuscript.

5.PLOS requires an ORCID iD for the corresponding author in Editorial Manager on papers submitted after December 6th, 2016. Please ensure that you have an ORCID iD and that it is validated in Editorial Manager. To do this, go to ‘Update my Information’ (in the upper left-hand corner of the main menu), and click on the Fetch/Validate link next to the ORCID field. This will take you to the ORCID site and allow you to create a new iD or authenticate a pre-existing iD in Editorial Manager. Please see the following video for instructions on linking an ORCID iD to your Editorial Manager account:
https://www.youtube.com/watch?v=_xcclfuvtxQ
[Note: HTML markup is below. Please do not edit.]

Reviewers' comments:

[NOTE: If reviewer comments were submitted as an attachment file, they will be attached to this email and accessible via the submission site. Please log into your account, locate the manuscript record, and check for the action link "View Attachments". If this link does not appear, there are no attachment files to be viewed.]

	While revising your submission, please upload your figure files to the Preflight Analysis and Conversion Engine (PACE) digital diagnostic tool, https://pacev2.apexcovantage.com/. PACE helps ensure that figures meet PLOS requirements. To use PACE, you must first register as a user. Registration is free. Then, login and navigate to the UPLOAD tab, where you will find detailed instructions on how to use the tool. If you encounter any issues or have any questions when using PACE, please email us at figures@plos.org. Please note that Supporting Information files do not need this step.<br><br>Thank you for your suggestion. We have uploaded and adjusted the pictures to PACE to meet PLOS ONE figures requirement. We attached the picture in submission.<br><br>Please send them (sequence data) to GenBank as well.<br>Thank you for your suggestion. We have deposited our all sequences data to Genbank as well with accession number MT324149 to MT324248.
**Additional Information:**	
**Question**	**Response**
**Financial Disclosure**<br><br>Enter a financial disclosure statement that describes the sources of funding for the work included in this submission. Review the submission guidelines for detailed requirements. View published research articles from *PLOS ONE* for specific examples.<br><br>This statement is required for submission and **will appear in the published article** if the submission is accepted. Please make sure it is accurate.<br><br>**Unfunded studies**<br>Enter: *The author(s) received no specific funding for this work.*<br><br>**Funded studies**<br>Enter a statement with the following details:<br>• Initials of the authors who received each award<br>• Grant numbers awarded to each author<br>• The full name of each funder<br>• URL of each funder website<br>• Did the sponsors or funders play any role in the study design, data collection and analysis, decision to publish, or preparation of the manuscript?<br>• **NO** - Include this sentence at the end of your statement: *The funders had no role in study design, data collection and analysis, decision to publish, or preparation of the manuscript.*<br>• **YES** - Specify the role(s) played.	This research was supported by Wildlife Conservation Society (WCS)-Indonesia, in collaboration with Faculty of Fisheries and Marine Sciences IPB University. SH received a scholarship from Indonesia Endowment Fund for Education (LPDP). The funders had no role in study design, data collection and analysis, decision to publish, or preparation of the manuscript.

Powered by Editorial Manager® and ProduXion Manager® from Aries Systems Corporation

* typeset	
**Competing Interests**<br><br>Use the instructions below to enter a competing interest statement for this submission. On behalf of all authors, disclose any competing interests that could be perceived to bias this work—acknowledging all financial support and any other relevant financial or non-financial competing interests.<br><br>This statement **will appear in the published article** if the submission is accepted. Please make sure it is accurate. View published research articles from *PLOS ONE* for specific examples.<br><br>**NO authors have competing interests**<br><br>Enter: *The authors have declared that no competing interests exist.*<br><br>**Authors with competing interests**<br><br>Enter competing interest details beginning with this statement:<br><br>*I have read the journal's policy and the authors of this manuscript have the following competing interests: [insert competing interests here]*<br><br>* typeset	The authors have declared that no competing interests exist.
**Ethics Statement**<br><br>Enter an ethics statement for this submission. This statement is required if the study involved:<br><br>• Human participants<br>• Human specimens or tissue<br>• Vertebrate animals or cephalopods<br>• Vertebrate embryos or tissues<br>• Field research<br><br>Write "N/A" if the submission does not require an ethics statement.	All specimens were collected in dead condition, no live specimens were obtained. Therefore, no approval from any institutional animal ethics committee was required. The samples collection and transport were in accordance with the regulation of Ministry of Marine Affairs and Fisheries of the Republic of Indonesia Number 5/PERMEN-KP/2018 on the prohibition of cowboy shark and hammerhead shark export from Indonesia. This study was also approved under permission number 276/BPSPL.03/PRL/X/2018 and 319/PNK/BPSPL.03/PK.230/REKOM/X/2018 by Ministry of Marine Affairs and Fisheries of the Republic of Indonesia.

Powered by Editorial Manager® and ProduXion Manager® from Aries Systems Corporation

General guidance is provided below. Consult the submission guidelines for detailed instructions. **Make sure that all information entered here is included in the Methods section of the manuscript.**<br><br>**Format for specific study types**<br><br>**Human Subject Research (involving human participants and/or tissue)**<br>• Give the name of the institutional review board or ethics committee that approved the study<br>• Include the approval number and/or a statement indicating approval of this research<br>• Indicate the form of consent obtained (written/oral) or the reason that consent was not obtained (e.g. the data were analyzed anonymously)<br><br>**Animal Research (involving vertebrate animals, embryos or tissues)**<br>• Provide the name of the Institutional Animal Care and Use Committee (IACUC) or other relevant ethics board that reviewed the study protocol, and indicate whether they approved this research or granted a formal waiver of ethical approval<br>• Include an approval number if one was obtained<br>• If the study involved *non-human primates*, add *additional details* about animal welfare and steps taken to ameliorate suffering<br>• If anesthesia, euthanasia, or any kind of animal sacrifice is part of the study, include briefly which substances and/or methods were applied<br><br>**Field Research**<br><br>Include the following details if this study involves the collection of plant, animal, or other materials from a natural setting:<br>• Field permit number<br>• Name of the institution or relevant body that granted permission	
**Data Availability**<br><br>Authors are required to make all data	Yes - all data are fully available without restriction

Powered by Editorial Manager® and ProduXion Manager® from Aries Systems Corporation

underlying the findings described fully available, without restriction, and from the time of publication. PLOS allows rare exceptions to address legal and ethical concerns. See the PLOS Data Policy and FAQ for detailed information.<br><br>A Data Availability Statement describing where the data can be found is required at submission. Your answers to this question constitute the Data Availability Statement and **will be published in the article**, if accepted.<br><br>**Important:** Stating 'data available on request from the author' is not sufficient. If your data are only available upon request, select 'No' for the first question and explain your exceptional situation in the text box.<br><br>Do the authors confirm that all data underlying the findings described in their manuscript are fully available without restriction?	
**Describe where the data may be found in full sentences. If you are copying our sample text, replace any instances of XXX with the appropriate details.**<br><br>• If the data are **held or will be held in a public repository**, include URLs, accession numbers or DOIs. If this information will only be available after acceptance, indicate this by ticking the box below. For example: *All XXX files are available from the XXX database (accession number(s) XXX, XXX.)*.<br>• If the data are all contained **within the manuscript and/or Supporting Information files**, enter the following: *All relevant data are within the manuscript and its Supporting Information files.*<br>• If neither of these applies but you are able to provide **details of access elsewhere**, with or without limitations, please do so. For example:<br><br>*Data cannot be shared publicly because of [XXX]. Data are available from the*	All relevant data are within the manuscript. All sequences data have been submitted to BOLD and can be access publicly.

XXX Institutional Data Access / Ethics Committee (contact via XXX) for researchers who meet the criteria for access to confidential data.<br><br>*The data underlying the results presented in the study are available from (include the name of the third party and contact information or URL).*<br>• This text is appropriate if the data are owned by a third party and authors do not have permission to share the data.<br><br>* typeset	
Additional data availability information:	Tick here if the URLs/accession numbers/DOIs will be available only after acceptance of the manuscript for publication so that we can ensure their inclusion before publication.

Powered by Editorial Manager® and ProduXion Manager® from Aries Systems Corporation

To:
Reviewer
PLOS ONE Journal

Dear Reviewer,

I am enclosing herewith our revised manuscript entitled **"Genetic connectivity of the scalloped hammerhead shark *Sphyrna lewini* (Griffith & Smith 1834) population across Indonesia and Western Indian Ocean"** re-submitted to **"PLOS ONE Journal"** for further evaluation.

Indonesia is one of the main shark fin exporters make this manuscript is very important information that will help understanding the genetic diversity in particular of scalloped hammerhead which evaluated as critical endangered species.

This information will be likely used for future assignment comparisons for example for market surveys, planning shark management and conservation area etc. We believe this manuscript makes an important contribution to the science of assessing and sustainably managing small- cale and data poor fisheries such shark fisheries, globally.

In a separate document we through point by point detailing how we have almost entirely accepted and incorporated their suggestions, and enhancing our manuscript in better.

Corresponding Author

Dr. Hawis Madduppa

Manuscript

Click here to access/download;Manuscript;3. Revised Manuscript_ CLEAN_SK1_BOAGUW-102 (2)_OK_HM.docx

Genetic connectivity of the scalloped hammerhead shark *Sphyrna lewini* (Griffith & Smith 1834) population across Indonesia and the Western Indian Ocean

Sutanto Hadi[1], Noviar Andayani[2,3], Effin Muttaqin[3], Benaya M Simeon[3], Muhammad Ichsan[3], Beginer Subhan[1] and Hawis Madduppa[1]*

[1] Department of Marine Science and Technology, Faculty of Fisheries and Marine Sciences, IPB University, Indonesia

[2] Department of Biology, Faculty of Mathematic and Natural Science, University of Indonesia, Depok, Indonesia

[3] Wildlife Conservation Society Indonesia, Bogor, Indonesia

*Corresponding author

Email: hawis@apps.ipb.ac.id

Abstract

Sphyrna lewini is an endangered species and its populations have been declining worldwide, including in Indonesia, because of overexploitation. However, there is a lack of recent information regarding ~~recent~~ the population structure in this species, to promote its proper management and conservation status in Indonesia. This study aimed to investigate the genetic diversity, population structure, and connectivity of the *S. lewini* population in Indonesia, i.e., in three major shark landing sites: Aceh (n = 41), Balikpapan (n = 30), and Lombok (n = 29); moreover, additional sequences were retrieved from West Papua (n = 14) and Western Indian Ocean (n = 65) populations. Analyses of the

mitochondrial *CO1* gene successfully identified a total of 179 sequences of *S. lewini*, with an average size of 594 bp, and 40 polymorphic loci in four haplotypes for the Indonesian population and in eight haplotypes for the Western Indian Ocean population. The overall values of genetic diversity in Indonesia were high (h = 0.717; π = 0.013), with the highest values recorded in Aceh (h = 0.668; π = 0.002) and the lowest detected in Papua (h = 0.143; π = 0.000), whereas the overall value was fairly low in the Western Indian Ocean (h = 0.232; π = 0.001). AMOVA and F_{ST} revealed three significant population subdivisions in Indonesia (F_{ST} = 0.442; P < 0.001), with separated populations for Aceh and West Papua, and a mixed population between Balikpapan and Lombok (F_{ST} = 0.044; P = 0.089); in contrast, a relatively insignificant differentiation was observed within the population of the Western Indian Ocean (F_{ST} = –0.013; P = 0.601) and a significant different was detected between the Indonesian and the Western Indian Ocean populations (F_{ST} = 0.740; IP < 0.001). The construction of A haplotype network provided evidence of gene flow and haplotype sharing between populations. This study indicated the presence of a complex population of *S. lewini* with limited connectivity in Indonesia, and in the Western Indian Ocean (at the regional scale), which will require co-management actions across regions.

Introduction

The scalloped hammerhead shark, *Sphyrna lewini* is considered a coastal species because of its need for nursery areas; its distribution circumglobally in tropical waters; and its abundance, both around continental margins and close to the coastal region and islands [1, 2]. This species has the unique modification of a laterally expanded head, which enhances the ability to navigate and follow geomagnetic orientations across the ocean [3–

5]. *S. lewini* has the capacity to move in high rates of dispersal. Female *S. lewini* specimens exhibit site fidelity to single nursery areas and exhibit no evidence of continued inter-oceanic migration. Male *S. lewini* species are spread over a large area across ocean waters, with clear evidence of cross-reproduction and gamete transmission [6].

S. lewini is ~~one~~ a threatened shark species, It has been estimated that one to three million specimens are killed annually worldwide because of fishing and the shark fin trade. This species was considered to be underexploited in 1999; however, it is currently listed as an endangered species in the IUCN red list [7] and in the Appendix II of The Convention on International Trade in Endangered Species (CITES). High exploitation of the population of *S. lewini* may have an impact on population structure, reducing the fecundity of the species and the genetic diversity of its populations [8]. Furthermore, this species has a low resilience to exploitation because of its late sexual maturity and a long lifetime in nature [9, 10].

The study of population genetics has become an important tool for understanding population connectivity, defining fisheries management, and conservation strategies [11]. The genetics of the population structure of *S. lewini*, which is important for fisheries stock management, has been widely investigated in different coastal areas and ocean basins on a global and regional scale [6, 12]. Duncan *et al*. [13] reported a global phylogeographic study of *S. lewini* that indicated that the Indo-West Pacific region is the center of diversity for tropical sharks, such as *S. lewini*, with a high and unique genetic diversity; however, no samples from Indonesia were included in that study, or in th[illegible]tudy reported by Ovenden *et al.* [14], which included limited samples from Indonesia.

The aim of this study was to investigate the genetic diversity, population structure, and connectivity of *S. lewini* among populations in Indonesia, where the populations of this

species are affected by fishing activities, and to observe the connectivity of this species at a regional scale in the Western Indian Ocean. Finally, we examined the implications of these results for species management and conservation.

Material and Methods

Ethics Statement

All specimens were already dead when collected, and no live specimens were obtained. Therefore, no approval from any institutional animal ethics committee was required. The sample collection and transportation were in accordance with the regulation of the Ministry of Marine Affairs and Fisheries of the Republic of Indonesia (Number 5/PERMEN-KP/2018) on the prohibition of cowboy shark and hammerhead shark export from Indonesia. This study was also approved by Ministry of Marine Affairs and Fisheries of the Republic of Indonesia, under permission numbers 276/BPSPL.03/PRL/X/2018 and 319/PNK/BPSPL.03/PK.230/REKOM/X/2018.

Tissue Sample Collection

A total of 100 tissue samples of *S. lewini* were collected from October 2017 to November 2018, including 41 samples from the Meulaboh and Aceh Jaya fishing ports (Aceh), 30 samples from a local shark landing in Manggar Baru, Balikpapan, and 29 samples from the Tanjung Luar fishing port (Lombok) (Table 1). The samples (~0.5 cm3) were dissected from flesh or fin tissues and preserved in sample bottles containing 96% ethanol.

Table 1. Sampling collection sites at major shark landing sites in Indonesia

Site	Geographic coordinate	Number of samples
**Aceh (ACH)**		
Meulaboh	N 04° 08’ 29” E 96° 07’ 55”	33
Aceh Jaya	N 04° 38’ 34” E 95° 34’ 58”	8
**Balikpapan (BPN)**		
Manggar Baru	S 01° 12’ 53” E 116° 58’ 24”	30
**Lombok (LOM)**		
Tanjung Luar	S 08° 46’ 39” E 116° 31’ 01”	29
**Total**		**100**

DNA Extraction, Amplification, and Sequencing

Extraction ~~of DNA~~ was performed at the Biodiversity and Biosystematics Laboratory, IPB University, according to the protocol of the gSYNC DNA extraction kit product. A fragment of the mitochondrial cytochrome oxidase subunit 1 (*CO1*) gene was amplified using the forward primer fish-BCL (5'–TCA ACY AAT CAY AAA GAT ATY GGC AC–3′) and the reverse primer fish-BCH (5'–ACT TCY GGG TGR CCR AAR AAT CA–3′) [15, 16] (Baldwin *et al.* 2009; Madduppa *et al.* 2016) in a 24 µL reaction mixture consisting of 3 µL of DNA template, 12.5 µL of MyTaq HS Red Mix, 9 µL of ddH_2O, 1.25 µL each of forward and reverse primers. The reaction mixture was processed in a polymerase chain reaction (PCR) on a thermocycler using modified cycling conditions [17, 18] (Prehadi *et al.* 2015; Hadi *et al.* 2019): pre-denaturation at 94°C for 15 s; followed by 38 cycles of denaturation at 94°C for 30 s, annealing at 50°C for 30 s, and extension at 72°C for 45 s; and a final extension at 72°C for 10 min. The amplicons were visualized by 1.5% agarose gel electrophoresis smeared with ethidium bromide at 100 V for 20 min. The gel was observed under UV light to identify bands indicating the presence of DNA fragments. Sequencing was performed using the BigDye Terminator v3.1 cycle

sequencing kit with an optimized protocol of the Applied Biosystems 3730 genetic analyzer at the 1st BASE, Malaysia.

Data Analysis

Genetic Diversity

The sequences of 179 mitochondrial *CO1* DNA sequences with an average length of 594 bp were edited and aligned using the ClustalW algorithm implemented in MEGA 6.06 [19]. Genetic diversity parameters, including the number of haplotypes, haplotype diversity (h), and nucleotide diversity (π), were calculated using the DNASp v6 [20] and Arlequin v.3.5 program [21]. We also reanalyzed additional *CO1* sequencing data of *S. lewini* from West Papua retrieved from GenBank (Table 2) (n = 14), which were obtained by Sembiring *et al.* [22], and sequences from previous studies performed in India (n = 6) [23], the United Arab Emirates (n = 30) [24], and Madagascar (n = 29) [25], to assess the genetic diversity in Indonesian and Western Indian Ocean populations.

Table 2. Localities, total number (n), and accession number of *CO1* gene sequences of *S. lewini* from Aceh, Balikpapan, Lombok, and Western Papua (Indonesia) and India, the United Arab Emirates, and Madagascar (Western Indian Ocean)

Locality	n	Accession number	Author
**Indonesia**			
Aceh	41	MT324187-219, MT324149-156	This study
Balikpapan	30	MT324157-186	This study
Lombok	29	MT324220-248	This study
West Papua	14	KF590254-55, KF590271-76, KF793729, KF793738-42	[22]
**Western Indian Ocean**			
India	6	KF899746-51	[23]
United Arab Emirates	30	KP177238-41, KP177241, KP177254, KP177262, KP177272, KP177285-99, KP177300-07	[24]
Madagascar	29	HQ171735-47, HQ171761-76	[25]

Population Structure

An analysis of molecular variance (AMOVA) and Wright's fixation index (F_{ST}) analysis [26] were performed for three major group: 1) within and among the four populations from Indonesia, 2) within and among the three populations from the Western Indian Ocean, and 3) comparison between populations from Indonesia and Western Indian Ocean using the Arlequin v.3.5 program (set up, 1000 permutations; significance level threshold, $\alpha = 0.05$), to estimate the overall extent of the genetic variation and differentiation level in Indonesia and the Western Indian Ocean. Population differentiation and its significance between sampling sites were also calculated with pairwise estimates. The value of F_{ST} ranges from zero to one; a low level of differentiation

was implied when the F_{ST} value was closer to zero; otherwise, a high level of differentiation was assumed when the value was closer to one [27, 28].

Genetic Connectivity

A haplotype network was constructed with a median-joining method in the Network v5.1.1.0 program [29] for all haplotypes detected in Indonesia and the Western Indian Ocean, to obtain haplotype connectivity in a broader spatial connection in the regional area of the Indian Ocean. The distribution of haplotypes for each location was also provided in a proper map, to show the clear distributions of haplotypes and genetic connectivity among the populations under study.

Results

Genetic Diversity

All sequences of *S. lewini* obtained in this study were deposited in the BOLD System with the Barcode Index Number (BIN) registry of BOLD:AAA2403 and database of GenBank with accession numbers MT324149-248 (Table 2). A total of 179 sequences of 594 bp of the mitochondrial *CO1* gene of *S. lewini* obtained from three sampling sites (Aceh, Balikpapan, and Lombok) and additional sequences of samples from West Papua and the West Indian Ocean region were used to generate a total of 11 haplotype variations with 40 polymorphic loci (Table 3).

Table 3. Forty polymorphic loci of 11 haplotypes from 179 sequences of the mitochondrial *CO1* gene of *S. lewini* samples from four localities in Indonesia and three localities in the Western Indian Ocean region

Haplotypes	Locus Position																																							
	**1**	**15**	**24**	**27**	**33**	**45**	**48**	**63**	**69**	**105**	**120**	**138**	**141**	**156**	**165**	**174**	**186**	**198**	**229**	**279**	**289**	**303**	**306**	**325**	**339**	**342**	**346**	**366**	**396**	**420**	**453**	**456**	**459**	**483**	**495**	**498**	**522**	**531**	**534**	**543**
H1*	A	T	A	C	T	T	G	T	T	A	T	C	G	T	T	C	C	T	T	C	G	T	T	C	C	A	C	T	T	C	C	T	T	C	T	C	C	T	C	T
H2*	.	.	.	A	.	.	.	.	.	G	.	A	T	.	C	T	T	C	C	T	.	C	C	T	T	C	.	C	C	T	.	.	.	A	.	T	.	C	.	C
H3	.	.	.	A	.	.	.	.	C	G	.	A	T	.	C	T	T	C	C	T	.	C	.	T	T	C	.	C	C	T	.	.	.	A	.	T	.	C	.	.
H4*	.	.	.	A	.	.	.	.	.	G	.	A	T	.	C	T	T	C	C	T	.	C	C	T	T	C	.	C	C	T	.	.	.	A	.	T	.	C	.	.
H5	.	.	.	.	.	.	.	.	.	.	.	.	.	.	.	.	.	.	.	.	.	.	.	.	.	.	.	.	.	.	.	.	G	.	G	.	.	.	.	.
H6	.	.	.	.	.	C	.	.	.	.	.	.	.	.	.	.	.	.	.	.	.	.	.	.	.	.	.	.	.	.	.	.	.	.	.	.	.	.	.	.
H7	.	.	C	.	.	.	.	.	.	.	.	.	.	G	.	.	.	.	.	.	.	.	.	.	.	.	.	.	.	.	.	.	.	.	.	.	.	.	.	.
H8	.	C	.	.	C	.	.	.	.	.	.	.	.	.	.	.	.	.	.	.	.	.	.	.	.	.	.	.	.	.	.	.	.	.	.	.	.	.	.	.
H9	.	.	.	.	.	.	.	.	.	.	G	.	.	.	.	.	.	G	.		T	.	.	.	.	.	.	.	.	.	.	.	.	.	.	.	.	.	G	.
H10	G	.	.	.	.	.	.	G	.	.	.	.	.	G	.	.	.	.	.	.	.	.	.	.	.	.	.	.	.	.	.	.	.	.	.	.	.	.	.	.
H11	.	.	.	.	.	.	.	.	.	.	.	.	.	.	.	.	.	.	.	.	.	.	.	.	.	.	T	.	.	.	T	.	.	.	C	.	T	.	.	.

Notes:

Nucleobase at each position is given for H1 and only nucleobases that were different from H1 are written for all other haplotypes. Nucleobases identical to H1 are indicated with dots (**.**)

*Three original haplotypes of the *S. lewini* populations from the three areas of study (Aceh, Balikpapan, and Lombok) in Indonesia. The remaining haplotypes were reanalyzed from previous studies.

The comparison of the genetic diversity of *S. lewini* based on haplotype and nucleotide diversity revealed the presence of variation (Table 4). The haplotype diversity (h) among the localities in Indonesia ranged from 0.143 to 0.668 and the nucleotide diversity (π) ranged from 0.000 to 0.020. The highest genetic diversity in Indonesia was observed for the population from Aceh (h = 0.668; π = 0.020), followed by the Balikpapan population, which exhibited a lower haplotype and nucleotide diversity (h = 0.646; π = 0.002), and the lowest genetic diversity was detected in West Papua (h = 0.143, π = 0.000). The *S. lewini* population from Lombok also exhibited a fairly low genetic diversity (h = 0.362; π = 0.001). However, the overall genetic diversity in Indonesia was relatively high (h = 0.717; π = 0.013). Conversely, the average genetic diversity of the Western Indian Ocean region was low and ranged from 0.000 to 0.467. The overall diversity in the Western Indian Ocean region was also low (h = 0.232; π = 0.001).

Table 4. Genetic diversity of *S. lewini* based on sample size (n), haplotype number (Hn), haplotype diversity (h), and nucleotide diversity (π) in samples from each site in Indonesia and the Western Indian Ocean region

Population	n	Genetic Diversity		
		Hn	h	π
**Indonesia**				
Aceh (ACH)	41	4	0.668	0.020
Balikpapan (BPN)	30	3	0.646	0.002
Lombok (LOM)	29	3	0.362	0.001
West Papua (WEP)	14	2	0.143	0.000
Overall Indonesia	114	4	0.717	0.013
**Western Indian Ocean**				
India (IND)	6	1	0.000	0.000
United Arab Emirates (UAE)	30	8	0.467	0.002
Madagascar (MDG)	29	1	0.000	0.000
Overall Western Indian Ocean	65	8	0.232	0.001

Population Structure

The analysis of the fixation index (F_{ST}) and the corresponding P-values between and within the four *S. lewini* populations (ACH, BPN, LOM, and WEP) from Indonesia and the three populations (IND, UAE, and MDG) from the Western Indian Ocean region are shown in Table 5. The overall F_{ST} value in Indonesia was significantly higher than that observed in other regions (F_{ST} = 0.442; P < 0.001), which indicated the presence of multiple subdivisions in Indonesia.

A low F_{ST} value with no significant differences was observed in the population from the Western Indian Ocean region (F_{ST} = –0.013; P = 0.601), while a comparison of the population structure between Indonesia and the Western Indian Ocean region yielded significant differences (F_{ST} = 0.740; P < 0.001). The pairwise F_{ST} values between the populations from the four locations in Indonesia and a comparison with those from Western Indian Ocean population are shown in Table 6. The overall pairwise analysis via the distance method indicated the presence of significant differences among the four populations from Indonesia, with the exception of BPN and LOM, which showed fairly low F_{ST} values and no significant p values. Furthermore, among the populations from Indonesia, the ACH population tended to be closer to the Western Indian Ocean population, as it exhibited a lower F_{ST} value (rather than F_{ST} range) compared with other localities from Indonesia to the Western Indian Ocean.

Table 5. Analysis of molecular variance (AMOVA) for percentage of variation (%), F_{ST} value, and significance level (P-value) in *S. lewini* samples from Indonesian populations, Western Indian Ocean populations, and between Indonesian and Western Indian Ocean populations

**Source of variation**	**df**	**Percentage of variation (%)**	**F_{ST} value**	***P* value**
**Indonesia**				
Among Populations	3	44.15	0.442	0.000
Within Populations	110	55.85		
Total	113			
**Western Indian Ocean**				
Among Populations	2	−1.31	−0.013	0.601
Within Populations	62	101.31		
Total	64			
**Indonesia vs. Western Indian Ocean**				

Among Populations	1	74.04	0.740	0.000
Within Populations	177	25.96		
**Total**	**178**			

Table 6. Pairwise F_{ST} values (below the diagonal) and *P*-values (above the diagonal) between the *S. lewini* populations from Aceh (ACH), Balikpapan (BPN), Lombok (LOM), West Papua (WEP), and Western Indian Ocean (WIO)

**Sample Sites**	**ACH**	**BPN**	**LOM**	**WEP**	**WIO**
**ACH**	-	0.000	0.000	0.000	0.000
**BPN**	0.427	-	0.091	0.001	0.000
**LOM**	0.438	0.044	-	0.000	0.000
**WEP**	0.398	0.495	0.736	-	0.000
**WIO**	0.509	0.965	0.973	0.975	-

Genetic Connectivity

A haplotype network analysis identified two main groups of haplotypes (Fig. 1), referred to as Clade A and Clade B henceforth. Clade A consisted of haplotype H1, which was observed in several regions, i.e., Aceh, India, United Arab Emirates, and Madagascar, whereas H5, H6, H7, H8, H9, H10, and H11 were present only in the samples from the United Arab Emirates. Clade B consisted of three haplotypes (H2, H3, and H4), which were spread evenly in Indonesia, although each region exhibited predominant haplotypes. Haplotypes H1, H2, H4, and H3 were predominant in Aceh, Balikpapan, Lombok, and West Papua, respectively (Fig. 2).

Fig 1. Haplotype network of the *S. lewini* (n = 179) population from Indonesia and the Western Indian Ocean region, which was constructed using the Median Joining method

Fig 2. Distribution of the 11 haplotypes of the *S. lewini* population from Indonesia and Western Indian Ocean at the regional scale

Discussion

Genetic Diversity

The overall genetic diversity of *S. lewini* at the haplotype and nucleotide levels was relatively high for populations in Indonesia. These findings are consistent with the results reported by Ovenden *et al.* [14], pertaining to the mitochondria control region of *S. lewini* from three localities in Indonesia, including Lombok, and the high genetic diversity of *S. lewini* observed at the global scale [13]. The scalloped hammerhead sharks ~~are~~ a highly migratory species with a wide distribution in tropical and warm-temperate waters and possibly move across oceanic waters to a distance of up to 1500 km [9]. In principle, because of migration capability and broad ecological niches, this species tends to have a higher genetic diversity than do species from narrow ecological niches [30]. High genetic diversity values are commonly related to large population sizes [31] and are promoted by several factors, such as local population sizes, fast generation times [32], high nucleotide substitution rates [33], and high gene flow between geographically distant populations.

The finding of a relatively high genetic diversity for *S. lewini* in Indonesia appears to be inconsistent with the general assumption that overexploitation of this species as both a target of fishing and bycatch has led to the decline of its populations worldwide [34]. However, the results obtained here for the population from Lombok may be relevant in this context; i.e., the lower genetic diversity detected at that location was probably driven by continuous fishing pressure. *S. lewini* were the top three targeted sharks at the Tanjung Luar fishing port in Lombok and have faced high fishing pressure over more than 40

years, which has led to its overfishing, with recent exploitation rates (E) reaching 0.59 [35, 36].

Furthermore, according to the global information system statistic of the FAO on fisheries [37], the hammerhead sharks (Sphyrnidae), including *S. lewini*, are important sharks that were highly exploited in the last two decades, with an estimated rapid increase in global capture, from 220 ton per year in 1985 up to approximately 10,362 tons per year in 2016. In the same period, the capture level in Indonesia also increased significantly, reaching approximately 1,492 tons in 2016. Moreover, Indonesia exhibited one of the highest volumes of sharks and rays caught on the global catches reported in the period of 2000–2011 [38].

Clarke *et al*. [39] reported similar findings regarding the mitochondrial DNA of the silky shark, *Carcharhinus falciformis*, which exhibited a high genetic diversity under circumstances of overexploitation. Elasmobranchs exhibit adaptability to environmental and anthropogenic stresses, which causes genetic bottlenecks because of their particular life histories [40]. Moreover, the population decline caused by recent fishery activities might be insufficient to reduce genetic diversity, particularly for species with a long life span (13–20 years), including *S. lewini* [41, 42].

However, the continuous decline in these populations, as was expected to occur for the *S. lewini* population from Lombok, was positively correlated with the loss of genetic diversity and created a population bottleneck [42, 43], as reported by Pinsky and Palumbi [44] in a meta-analysis of several marine fish.

Population Structure

Our findings indicated the presence of homogeneity among the *S. lewini* populations from Balikpapan and Lombok. The pairwise F_{ST} analysis detected no significant genetic differentiation in these two populations with lowest value of F_{ST}. These finding complements those of previous studies conducted in Indo-Australian waters. Ovenden *et al.* [14] reported similar evidence for the mitochondrial control region regarding population structure, with no differentiation between two populations of *S. lewini* (Lombok and northern Australia). This pattern of single-stock population seems to suggest that these localities are a migration zone of *S. lewini* and that a reproductive movement might occur in the coastal areas of these locations, although there are strong Indonesian throughflow currents between Kalimantan and Sulawesi Island. Adult *S. lewini* specimens are highly migratory, with a large body size that supports the high dispersal capacity of this species; hence, they exhibit the possibility of overcoming that geographical barrier. In contrast, we obtained different results from the comparison among these two populations from central Indonesia and one population from Aceh (western Indonesia) and one population from West Papua (eastern Indonesia), which exhibited strong genetic differentiation. These regions were spatially separated by a long distance, with the possibility of the existence of more complex barriers, both inter-island barriers and anthropogenic factors pertaining to a high commercial and artisanal fishing pressure, such as those found along the southern and northern coasts of Java.

Genetic Connectivity

We obtained an interesting result regarding haplotype sharing between the populations from Indonesia and the Indian Ocean population. H1 was a unique haplotype found in Aceh, Indonesia, and was correlated with the most common haplotype observed in

samples from the three locations of the Western Indian Ocean region (India, United Arab Emirates, and Madagascar), which indicates the presence of connectivity between the populations from Aceh and those from the Western Indian Ocean region. Schott and McCreary [45] reported that the Indian Ocean seasonal current carried water from north Sumatra through the Arabian Sea to Madagascar, which represents a possible connectivity channel between the two regions, thus allowing *S. lewini* to migrate and leading to ~~cross-~~breeding. The similarity between the predominant haplotype (H1) of *S. lewini* from Aceh with that of the populations from India, United Arab Emirates, and Madagascar evidenced a genetic sharing process among populations in the Indian Ocean region. However, the *S. lewini* populations from Balikpapan, Lombok, and West Papua seemed to be isolated from the population from the Western Indian Ocean, and shared a haplotype network exclusively in eastern Indonesian waters.

Conservation Implications

The overall high genetic diversity of the *S. lewini* populations in Indonesia suggests that this species has not yet experienced a genetic loss because of exploitation pressure. However, the lower genetic diversity of *S. lewini* from Lombok and West Papua indicated a higher risk of loss of genetic diversity, which probably was the result of high fisheries pressure. Our genetic assessment of *S. lewini* samples from four localities revealed that a single stock exist between Lombok and Balikpapan, while a separate stock was observed for Aceh and West Papua, indicating that the management of this species should occur on a stock-based approach at least on three mitochondrial-stock conservation management units. The complex population of *S. lewini* with limited connectivity observed in Indonesia and the Western Indian Ocean region indicated the importance of promoting

collaborative management strategies among Indonesian management agencies and in conjunction with Western Indian Ocean agencies at the regional scale.

Conclusion

This study provided important findings regarding the population structure of *S. lewini* in Indonesia, with a high genetic diversity and three significant subdivisions that indicated the capability of the population to adapt to rapid environmental changes and pressure, including fishing activities. However, the lower genetic diversity detected in Lombok and West Papua should be considered. The genetic sharing detected among the populations from Indonesia and also those from the Western Indian Ocean proved the connectivity among these populations. A collaborative action across regions is needed to promote sustainable management and conservation purposes, both in Indonesia and at the regional scale in the Western Indian Ocean area.

Acknowledgement

We would like to thank to the institutions and individuals that have made our study possible: colleagues from Marine Biodiversity and Biosystematics Lab (BIODIVSI), Faculty of Marine Science and Technology, IPB University for their help during research. We also appreciated to BPSPL Satker Balikpapan, Listian Nova and Mr. Hery, Head of TPI Manggar Balikpapan for their help during collecting samples, and to reviewers and proof readers who have provided highly constructive suggestions for better writing of the paper.

References

1. Klimley AP, Butler SB. Immigration and emigration of a pelagic fish assemblage to seamounts in the Gulf of California related to water mass movements using satellite imagery. Mar Ecol Prog Ser. 1988;49: 11-20.
2. Compagno LJV. FAO Species Catalogue: Sharks of the world. Rome: Food And Agriculture Organization;1984.
3. Montgomery JC, Walker MM. Orientation and navigation in elasmobranchs: which way forward? Environ Biol Fishes. 2001;60: 109-116.
4. Kajiura SM, Holland KN. Electroreception in juvenile scalloped hammerhead and sandbar sharks. J Exp Biol. 2002;205: 3609-3621.
5. Meyer CL, Holland KN, Papastamatiou YP. Sharks can detect changes in the geomagnetic field. J R Soc Interface. 2005;2: 129-130.
6. Daly-Engel TS, Seraphin KD, Holland KN, Coffey JP, Nance HA, Toonen RJ, et al. Global phylogeography with mixed-marker analysis reveals male-mediated dispersal in the endangered scalloped hammerhead shark (*Sphyrna lewini*). PLoS ONE. 2012;7(1): e29986. doi: 10.1371/journal.pone.0029986.
7. Castillo-olguin E, Aribe-Alcocer M, Diaz-Jaimes P. Assessment of the population genetic structure of *Sphyrna lewini* to identify conservation units in the Mexican Pacific. Ciencias Marinas. 2012;38(4): 635-652. doi: 10.7773/cm.v38i4.2110.
8. Ward RD. Genetics in fisheries management. Hidrobiologia. 2000;420: 191–201.
9. Kohler NE, Turner PA. Shark tagging: a review of conventional methods and studies. Environ Biol Fishes. 2001;60: 191-223.
10. Bessudo S, Soler GA, Kimley PA, Ketchum J, Arauz R, Hearn A, et al. Vertical and horizontal movements of scalloped hamemerhead shark (*Sphyrna lewini*) around

Malpelo and Cocos islands (Tropical Eastern Pacific) using satellite telemetry. Boletín de Investigaciones Marinas y Costeras. 2011;40: 91-106.

11. Cardeñosa D, Hyde J, Caballero S. Genetic diversity and population structure of the pelagic thresher shark (*Alopias pelagicus*) in the Pacific Ocean: evidence for two evolutionarily significant units. PLoS ONE. 2014;9(10): e110193. doi: 10.1371/journal.pone.0110193.
12. Quintanilla S, Gómez A, Mariño-Ramírez C, Sorzano C, Bessudo S, Soler G, et al. Conservation genetics of the scalloped hammerhead shark in the Pacific Coast of Colombia. J Hered. 2015;106: 448-458. doi:10.1093/jhered/esv050.
13. Duncan KM, Martin AP, Bowen BW, De Coute HG. Global phylogeography of the scalloped hammerhead shark (Sphyrna lewini). Mol Ecol. 2006;15:2239-51.
14. Ovenden JR, Kashiwagi T, Broderick D, Giles J, Salini J. The extent of population genetic subdivision differs among four co-distributed shark species in the Indo-Australian archipelago. BMC Evol Biol. 2009;9: 40. doi:10.1186/1471-2148-9-40.
15. Baldwin CC, Mounts JH, Smith DG, Weigt LA. Genetic identification and color descriptions of early life-history stages of Belizean *Phaeoptyx* and *Astrapogon* (Teleostei: Apogonidae) with comments on identification of adult *Phaeoptyx*. Zootaxa. 2009;2008: 1-22.
16. Madduppa H, Ayuningtyas RU, Subhan B, Arafat D. Exploited but unevaluated: DNA barcoding reveals skates and stingrays (Chordata, Chondrichthyes) species landed in the Indonesian fish market. IJMS. 2016;21: 77-84.
17. Prehadi, Sembiring A, Kurniasih EM, Rahmad, Arafat D, Subhan B et al. DNA barcoding and phylogenetic reconstruction of shark species landed in Muncar

fisheries landing site in comparison with Southern Java fishing port. Biodiversitas. 2015;16: 55-61.

18. Hadi S, Anggraini NP, Muttaqin E, Simeon BM, Subhan B, Madduppa H. Genetic diversity of the endangered species Sphyrna lewini (Griffith and Smith 1834) in Lombok based on mitochondrial DNA. IOP Conf. Ser.: Earth Environ. Sci. 2019;236: 012024.

19. Tamura K, Stecher G, Peterson D, Filipski A, Kumar S. Molecular Evolutionary Genetics Analysis Version 6.0. Mol Biol Evol. 2013;30(12): 2725-2729. doi: 10.1093/molbev/mst197.

20. Rozas J, Ferrer-Mata A, Sánchez-DelBarriol JC, Guirao-Rico S, Librado P, Ramos-Onsins SE. DnaSP v6: DNA Sequence polymorphism analysis of large datasets. Mol Biol Evol. 2017;34: 3299-3302.

21. Excoffier L, H.E. L. Lischer. Arlequin suite ver 3.5: A new series of programs to perform population genetics analyses under Linux and Windows. Mol Ecol Resour. 2010;10: 564-567.

22. Sembiring A, Pertiwi NPD, Mahardini A, Wulandari R, Kurniasih EM, Kuncoro AW, et al. DNA barcoding reveals targeted fisheries for endangered sharks in Indonesia. Fisheries Research. 2015;164: 130-134. doi: 10.1016/j.fishres.2014.11.003.

23. Bineesh KK, Gopalakrishnan A, Akhilesh KV, Sajeela KA, Abdussamad EM, Pillai NGK, et al. DNA barcoding reveals species composition of sharks and rays in the Indian commercial fishery. Mitochondrial DNA. 2016. doi: 10.3109/19401736.2015.1137900.

24. Jabado RW, Ghais SMA, Hamza W, Henderson AC, Spaet JLY, Shivji MS, et al. The trade in sharks and their products in the United Arab Emirates. Biol Conserv. 2015;181: 190-198. doi: 10.1016/j.biocon.2014.10.032.
25. Doukakis P, Hanner R, Shivji M, Bartholomew C, Chapman D, Wong E, et al. Applying Genetic Techniques to Study Remote Shark Fisheries in Northeastern Madagascar. Mitochondrial DNA. 2011;22(S1): 15-20.
26. Wright S. The genetical structure of populations. Annals of Eugenics. 1951;15: 323-354.
27. Wright S. Evolution and the Genetics of Populations. 4th vol. Variability within and among Natural Populations. Chicago: University of Chicago Press; 1978.
28. Hartl DL, Clark AG. Principles of Population Genetics. 3nd ed. Sunderland: Sinauer Associates Inc; 1997.
29. Bandelt H-J, Forster P, Röhl A. Median-joining networks for inferring intraspecific phylogenies. Mol Biol Evol. 1999;16: 37-48.
30. Habel JC, Schmitt T. Vanishing of the common species: Empty habitats and the role of genetic diversity. Biol. Conserv. 1996;218: 211-216. doi: 10.1016/j.biocon.2017.12.018.
31. Frankham R. Relationship of Genetic Variation to Population Size in Wildlife. Conserv. Biol. 1996;10(6): 1500-1508. doi:10.1046/j.1523-1739.1996.10061500.x.
32. Hague MTJ, Routman EJ. Does population size affect genetic diversity? A test with sympatric lizard species. Heredity. 2016;116: 92-98. doi: 10.1038/hdy.2015.76.
33. Martin AP, Naylor GJP, Palumbi SR. Rates of mitochondrial DNA evolution in sharks are slow compared with mammals. Nature. 1992;357: 153-157. doi: 10.1038/357153a0.

34. Compagno LJV, Dando M, Fowler SL. Sharks of the World. Princeton: Princeton University Press; 2005.
35. Hordyk A, Ono K, Valencia S, Loneragan N, Prince J. A novel length-based empirical estimation method of spawning potential ratio (SPR), and tests of its performance, for small-scale, data-poor fisheries. ICES J Mar Sci. 2015;2-15. doi: 10.1093/icesjms/fsu004.
36. Simeon BM, Agustina S, Muttaqin E, Yulianto I, Ichsan M, Muhsin M. Laporan teknis: Pemanfaatan Hasil Tangkap Hiu dan Pari di Tanjung Luar, Provinsi Nusa Tenggara Barat. Bogor: Wildlife Conservation Society-Indonesia Program; 2017.
37. Food and Agriculture Organization (FAO). Fisheries and Aquaculture Information and Statistics Service: Global Capture Production 1950-2017; 2017 [cited 2020 Mar 5]. Database: Indonesian hammerhead shark capture [Internet]. http://www.fao.org/fishery/statistics/global-capture-production/query/es.
38. Dent F, Clarke. State of the Global Market for Shark Products. Rome: Food And Agriculture Organization; 2015.
39. Clarke CR, Karl SA, Horn RL, Bernard AM, Lea JS, Hazin FH et al. Global mitochondrial DNA phylogeography and population structure of the silky shark, *Carcharhinus falciformis.* Mar Biol. 2015;162(5): 945-955. doi: 10.1007/s00227-015-2636-6
40. Chapman DD, Simpfendorfer CA, Wiley TR, Poulakis GR, Curtis C, Tringali M et al. Genetic diversity despite population collapse in a critically endangered marine fish: the smalltooth sawfish (*Pristis pectinata*). J Hered. 2011;102: 643-652.

41. Sánchez-de Ita JA, Quiñónez-Velázquez C, Galván-Magaña F, Bocanegra-Castillo N, Félix-Uraga R. Age and growth of the silky shark *Carcharhinus falciformis* from the west coast of Baja California Sur, Mexico. J App Ichthyol. 2011;27: 20-24.
42. Nei M, Maruyama T, Chakraborty R. The bottleneck effect and genetic variability in populations. Evolution. 1975;29(1): 1. doi:10.2307/2407137.
43. Beebee T, Rowe G. An Introduction to Molecular Ecology. 2nd ed. New York: Oxford University Press Inc; 2008.
44. Pinsky ML, Palumbi SR. Meta-analysis reveals lower genetic diversity in overfished populations. Mol Ecol. 2013;23(1): 29-39. doi:10.1111/mec.12509.
45. Schott FA, McCreary JP. The monsoon circulation of the Indian Ocean. Prog Oceanogr. 2001;51: 1-123. doi: 10.1016/S0079-6611(01)00083-0.

Figure 1

Click here to access/download;Figure;FIG 1 (1).tif

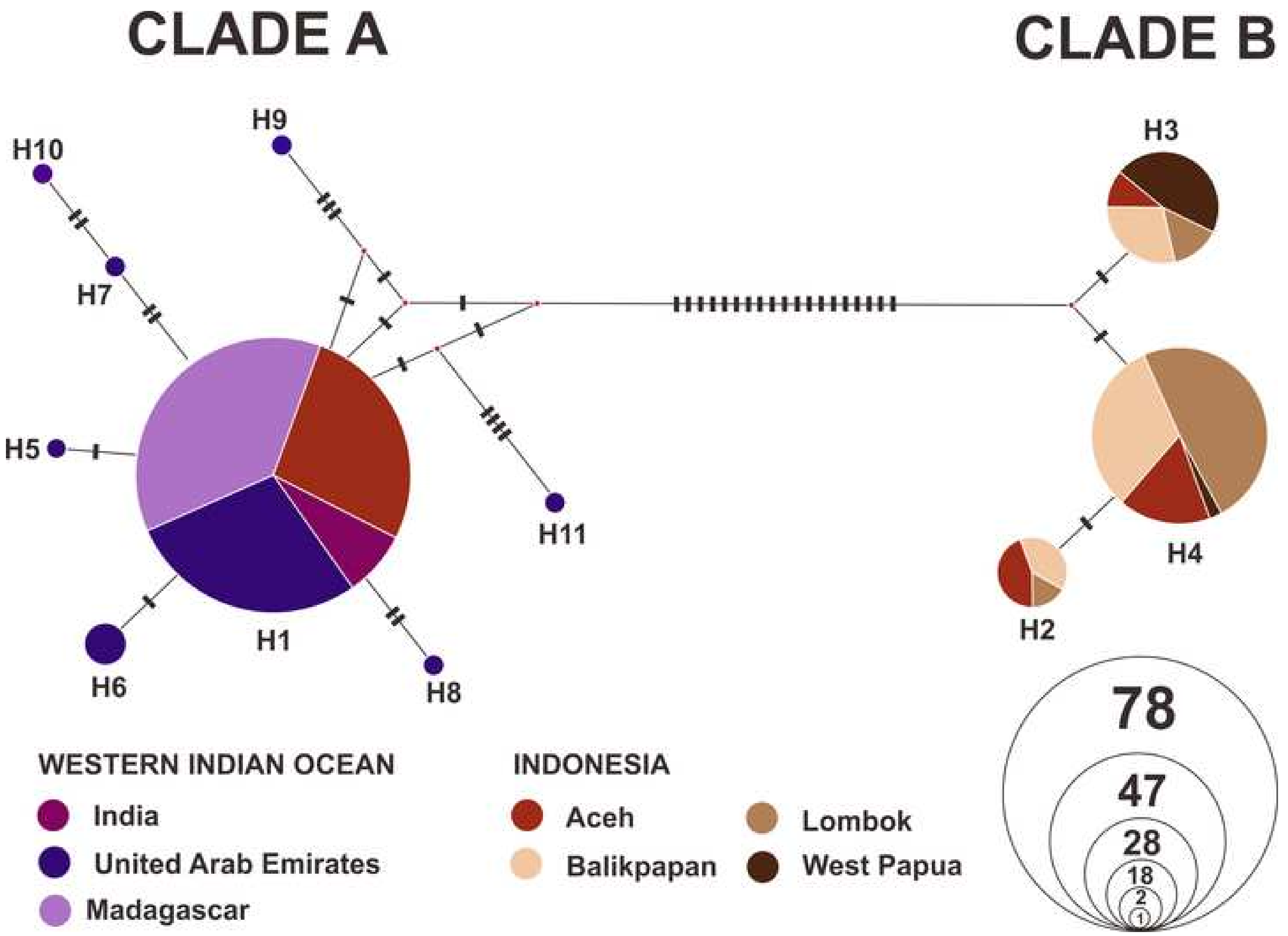

Figure 2

Click here to access/download;Figure;FIG 2 (1).tif

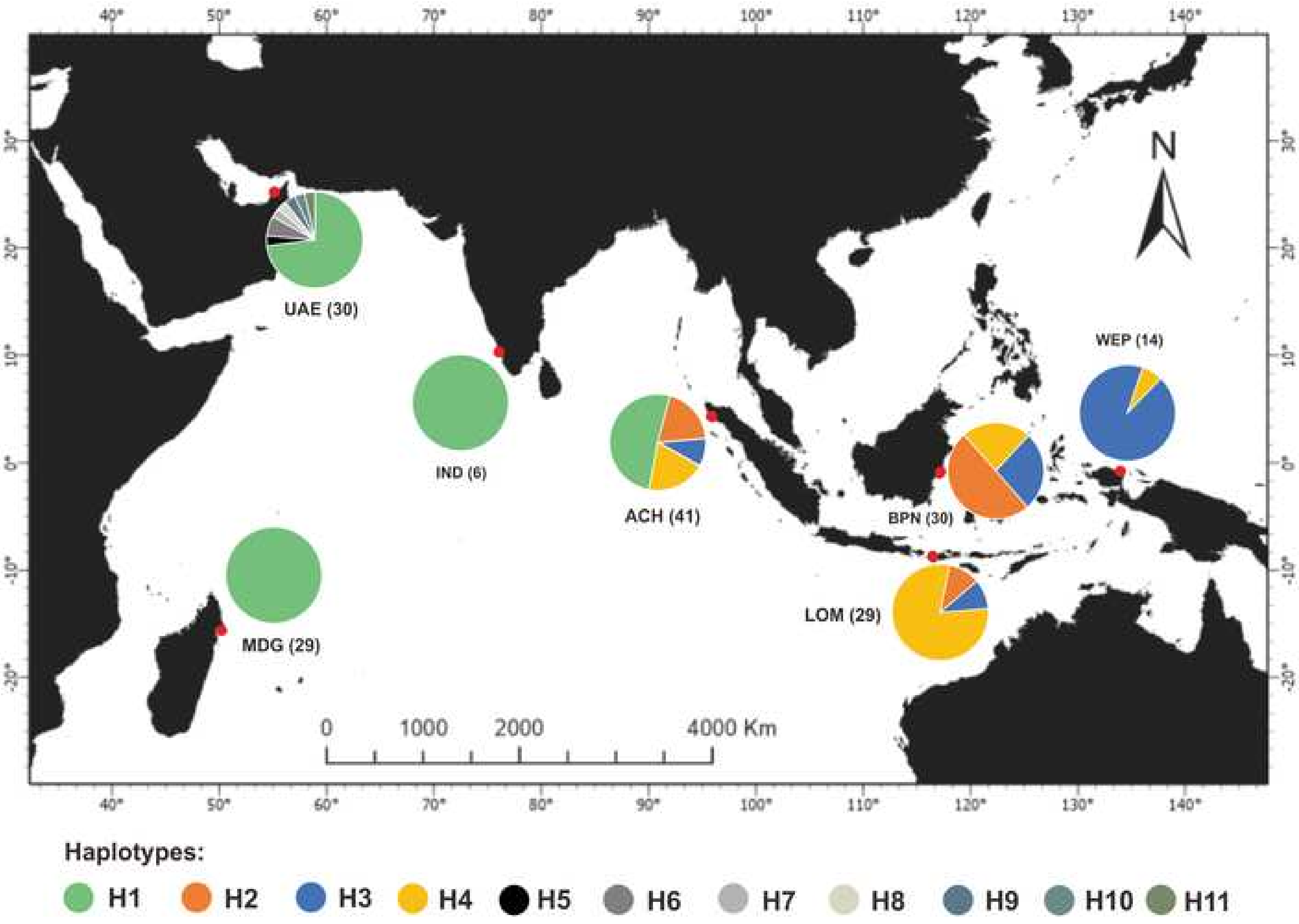

Genetic connectivity of the scalloped hammerhead shark *Sphyrna lewini* (Griffith & Smith 1834) population across Indonesia and the Western Indian Ocean

Sutanto Hadi[1], Noviar Andayani[2,3], Effin Muttaqin[3], Benaya M Simeon[3], Muhammad Ichsan[3], Beginer Subhan[1] and Hawis Madduppa[1]*

[1] Department of Marine Science and Technology, Faculty of Fisheries and Marine Sciences, IPB University, Indonesia

[2] Department of Biology, Faculty of Mathematic and Natural Science, University of Indonesia, Depok, Indonesia

[3] Wildlife Conservation Society Indonesia, Bogor, Indonesia

*Corresponding author

Email: hawis@apps.ipb.ac.id

Abstract

Sphyrna lewini is ~~the~~ an endangered species ~~which~~ and its populations have been declining worldwide, including in Indonesia, ~~due to~~because of overexploitation. However, there is a lack of recent information regarding recent the population structure in this species, to promote its proper management and conservation status in Indonesia. This study aimed to investigate the genetic diversity, population structure, and connectivity of the *S. lewini* population in Indonesia, i.e., ~~from~~ in three major shark landing sites: ~~in~~ Aceh (n = 41), Balikpapan (n = 30), and Lombok (n = 29);~~,~~ ~~and~~ moreover, additional sequences were retrieved from West Papua (n = 14) and Western Indian Ocean

Commented [Editor1]: **Tip: Flow and transition:** To create an easy flow of ideas, transition words such as however, therefore, moreover, etc. can be used. This usage enhances coherence of ideas in the paragraph and the manuscript on the whole.

Commented [SH2R1]: Thank you for your useful tips.

~~population~~ (n = 65) populations. Analyses of the mitochondrial *CO1* gene successfully identified a total of 179 sequences of *S. lewini*, with an average size of 594 bp~~, nucleotide with~~ and 40 polymorphic loci in four haplotypes for the Indonesian population and in eight haplotypes for the Western Indian Ocean population. The overall values of genetic diversity in Indonesia ~~was~~ were high (h = 0.717; π = 0.013), with the highest ~~was~~ values recorded in Aceh (h = 0.668; π = 0.002)~~,~~ and the lowest detected~~was~~ in Papua (h = 0.143; π = 0.000~~1~~), ~~while~~ whereas ~~in Western Indian Ocean~~ the overall value was fairly low in the Western Indian Ocean (h = 0.232; π = 0.001). ~~The~~ AMOVA and F_{ST} revealed three significant population subdivisions in Indonesia (F_{ST} = 0.442; ~~*p*~~ *P* < 0.001), with separated populations for Aceh and West Papua, and a ~~mixing~~ mixed population between Balikpapan and Lombok (F_{ST} = 0.044; ~~*p*~~ *P* = 0.089);~~,~~ ~~whereas~~ in contrast, a relatively ~~no~~ insignificant differentiation was observed within the population ~~in~~ of the Western Indian Ocean (F_{ST} = –0.013; ~~*p*~~ *P* = 0.601)~~,~~ and a significant different ~~level showed by~~was detected between the Indonesian ~~population compared with~~and the Western Indian Ocean populations (F_{ST} = 0.740; ~~p~~ IP < 0.001). The construction of A haplotype network ~~exhibited~~ provided evidence of gene flow and haplotype sharing between populations. This ~~result~~ study indicated the presence of a complex ~~and limited connectivity~~ population of *S. lewini* with limited connectivity in Indonesia, and ~~between~~ in the Western Indian Ocean (at the~~in~~ regional scale), which will ~~make~~ require co-management actions ~~necessary~~ across regions.

Introduction

The scalloped hammerhead shark, *Sphyrna lewini* ~~(*S. lewini*)~~ is considered a coastal species ~~due to~~because of ~~their~~ its need for nursery areas;~~,~~ ~~distributed~~ its distribution

circumglobally ~~along~~ in tropical waters;~~,~~ ~~abundant~~ and its abundance, both around continental margins and close to the coastal region and islands [1, ~~-~~2]. This species has ~~a~~ the unique modification of a laterally expanded head, which ~~enhanced~~ enhances the~~m~~ ~~with the~~ ability to navigate and follow geomagnetic orientations across the ocean [3–~~-~~5]. *S. lewini* has ~~a~~ the capacity to move in high rates of dispersal. Female *S. lewini* specimens exhibit~~s~~ site fidelity to single nursery areas and ~~indicate~~ exhibit no evidence of ~~continuing~~ continued ~~inter~~ inter-oceanic migration. Male *S. lewini* species are spread ~~in~~ over a ~~long~~ large area~~distance spatial~~ across ocean waters, with clear evidence ~~in~~ of ~~cross~~ cross-reproduction and gamete~~s~~ transmission [6]. *S. lewini* ~~-~~is one ~~of the~~a threatened shark species, ~~of sharks which~~It has been estimated that one to three million specimens ~~were~~ are killed annually worldwide ~~due to~~because of fishing and the shark fin~~s~~ trade. This species was considered to be ~~an~~ underexploited ~~species~~ in 1999;~~,~~ however, it is currently listed ~~to be~~as an endangered species in the IUCN red list [7] and in the Appendix II of The Convention on International Trade in Endangered Species (CITES). High exploitation of the population of ~~-~~*S. lewini* ~~-~~may ~~has~~ have an impact on ~~the~~ population structure, reducing the fecundity of the species and the genetic diversity of its populations [8]. Furthermore, this species has a low resilience to exploitation ~~since~~ because of its late sexual maturity and a long lifetime in nature [9, ~~-~~10]. The study of population genetics ~~have~~ has become an important tool for understanding population connectivity, defining fisheries management, and conservation strategies [11]. ~~Genetic~~ The genetics of the population structure of *S. lewini*, which is important ~~to~~ for fisheries stock management, has been widely investigated in different coastal areas and ocean basins on a global and regional scale [6, 12]. Duncan *et al.* [13] ~~revealed~~ reported a global phylogeographic study of ~~-~~*S. lewini* ~~which~~ that indicated that the Indo-West

Pacific region is~~was a~~ the ~~centre~~center of diversity for tropical sharks, ~~like~~ such as *S. lewini*, with a high and unique genetic diversity;~~,~~ however, no samples from Indonesia were included in that study, ~~also~~ or in the study reported by Ovenden *et al.* [14], which ~~were conducted with~~included limited samples from Indonesia.

The aim of this study was to investigate the genetic diversity, population structure, and connectivity of *S. lewini* among populations in Indonesia, where the populations of this species are affected by fishing activities,~~,~~ and to observe the connectivity of this species at ~~in~~ a regional scale in the Western Indian Ocean.~~,~~ ~~and~~Finally, ~~also suggest~~ we examined the implications of these results for ~~the~~ species management and conservation.

Material and Methods

Ethics Statement

All specimens were already dead when collected, and no live specimens were obtained. Therefore, no approval from any institutional animal ethics committee was required. The sample collection and transportation were in accordance with the regulation of the Ministry of Marine Affairs and Fisheries of the Republic of Indonesia (Number 5/PERMEN-KP/2018) on the prohibition of cowboy shark and hammerhead shark export from Indonesia. This study was also approved by Ministry of Marine Affairs and Fisheries of the Republic of Indonesia, under permission numbers 276/BPSPL.03/PRL/X/2018 and 319/PNK/BPSPL.03/PK.230/REKOM/X/2018 ~~by Ministry of Marine Affairs and Fisheries of the Republic of Indonesia~~.

Tissue Sample Collection

A total of 100 tissue samples of *S. lewini* were collected from October 2017 to November 2018, ~~consisting of~~including 41 samples from the Meulaboh and Aceh Jaya fishing ports (Aceh), 30 samples from a local shark landing in Manggar Baru, Balikpapan, and 29 samples from the Tanjung Luar fishing port (Lombok) (Table 1). The samples (~0.5 cm3) were dissected from flesh or fin tissues ~~approximately 0.5 cm^3~~ and preserved in sample bottles ~~contained~~ containing 96% ethanol.

Table 1. Sampling collection sites at ~~from~~ major shark landing sites in Indonesia

Site	Geographic coordinate	Number of samples
**Aceh (ACH)**		
Meulaboh	N 04° 08’ 29” E 96° 07’ 55”	33
Aceh Jaya	N 04° 38’ 34” E 95° 34’ 58”	8
**Balikpapan (BPN)**		
Manggar Baru	S 01° 12’ 53” E 116° 58’ 24”	30
**Lombok (LOM)**		
Tanjung Luar	S 08° 46’ 39” E 116° 31’ 01”	29
**Total**		**100**

DNA Extraction, Amplification, and Sequencing

Extraction of DNA was ~~conducted~~ performed ~~in~~ at the Biodiversity and Biosystematics Laboratory, IPB University, ~~following~~ according to the protocol of the gSYNC DNA extraction kit product. A fragment of the mitochondrial cytochrome oxidase subunit 1 ~~I gene~~ (~~*COI*~~*CO1*) gene was amplified using the forward primer fish-BCL (5'–TCA ACY AAT CAY AAA GAT ATY GGC AC–'3') and the reverse primer fish-BCH (5'–ACT TCY GGG TGR CCR AAR AAT CA–'3') [15, -16] (Baldwin *et al.* 2009; Madduppa *et al.* 2016) in a 24 µL reaction mixture ~~consisted~~ consisting of 3 µL of DNA template, 12.5 µL of MyTaq HS Red Mix, 9 µL of ddH$_2$O, 1.25 µL each of forward and reverse primers ~~respectively~~. ~~Mixture~~ The reaction mixture~~product~~ was processed in a polymerase chain

reaction (PCR) on a thermocycler following usinga modified cycle cycling conditions [17, -18] (Prehadi *et al.* 2015; Hadi *et al.* 2019):. The step was initiated with pre pre-denaturation at 94-°C for 15 s;, followed by 38 cycles of denaturation at 94-°C for 30 sec, annealing at 50-°C for 30 sec, and extension at 72-°C for 45 sec;, and finished with a final extension at 72–°C for 10 min. The amplicons were visualized by 1.5% agarose gel electrophoresis agarose 1.5 % smeared with ethidium bromide at 100 V for 20 min. The gel was observed under UV light to determine identifya bands indicating the presence of DNA fragments. Sequence Sequencingreaction was performed using the BigDye Terminator v3.1 cycle sequencing kit with an optimized protocol of the Applied Biosystems 3730 genetic analyser analyzer at the 1st BASE, Malaysia.

Data Analysis

Genetic Diversity

Sequences The sequences of 179 mitochondrial DNA *CO1* DNA sequences with an average length of 594 bp in length were edited and aligned using the CrustalW ClustalW algorithm implemented in MEGA 6.06 [19]. Genetic diversity parameters, including the number of haplotypes, haplotype diversity (h), and nucleotide diversity (π), were calculated using the DNASp v6 [20] and Arlequin v.3.5 program [21]. We also reanalysed reanalyzed additional *COI1* sequences sequencing data of *S. lewini* from West Papua retrieved from GenBank (Table 2) from West Papua (n = 14), which were obtained by Sembiring *et al.* [22], and sequences from previous studies performed in India (n = 6) [23], the United Arab Emirates (n = 30) [24], and Madagascar (n = 29) [25], to show assess the genetic diversity in Indonesian and Western Indian Ocean populations.

Table 2. Localities, total number (n), and accession number of ~~*COI*~~ *CO1* gene sequences of *S. lewini* ~~(retrieved from GenBank)~~ ~~in~~ from Aceh, Balikpapan, Lombok, and Western Papua (Indonesia) and~~,~~ India, the United Arab Emirates, and Madagascar (Western Indian Ocean) ~~retrieved from GenBank~~

~~**Localities**~~**Locality**	**n**	**Accession number**	**Author**
**Indonesia**			
Aceh	41	MT324187-219, MT324149-156	This study
Balikpapan	30	MT324157-186	This study
Lombok	29	MT324220-248	This study
West Papua	14	KF590254-55, KF590271-76, KF793729, KF793738-42	[22]
**Western Indian Ocean**			
India	6	KF899746-51	[23]
United Arab Emirates	30	KP177238-41, KP177241, KP177254, KP177262, KP177272, KP177285-99, KP177300-07	[24]
Madagascar	29	HQ171735-47, HQ171761-76	[25]

Formatted Table

Formatted: Font: 11 pt

Formatted: Font: 11 pt

Formatted: Indent: Left: 0"

Formatted: Left

Formatted: Font: 11 pt

Formatted: Indent: Left: 0"

Formatted: Left

Formatted: Left

Population Structure

An analysis of molecular variance (AMOVA) and Wright's fixation index (F_{ST}) analysis [26] were ~~conducted~~ performed for three major group: 1) within and among the four populations ~~in~~ from Indonesia, 2) within and among the three populations ~~in~~ from the Western Indian Ocean, and 3) comparison between populations from Indonesia and Western Indian Ocean using the Arlequin v.3.5 program (set~~ting~~ up, 1000 permutations;~~,~~ significance level threshold, α = 0.05 ~~for significance level threshold~~), to estimate the overall extent of the genetic variation and differentiation level in Indonesia and the Western Indian Ocean. Population differentiation and its significance between sampling sites were also calculated with pairwise estimates. The value of F_{ST} ~~is a~~ ranges ~~of~~ from zero to one;~~,~~ a low level of differentiation was implied ~~from the closer of~~when the F_{ST} value was closer to zero;~~,~~ otherwise, ~~the~~ a high level of differentiation was assumed when the value was~~will be~~ closer to one [27, ~~-~~28].

Genetic Connectivity

A haplotype network was constructed with a median-joining method in the Network v5.1.1.0 program [29] for all haplotypes ~~found~~ detected in Indonesia and the Western Indian Ocean, to obtain haplotype connectivity in a broader spatial connection in the regional area ~~in~~ of the Indian Ocean. ~~Distribution~~ The distribution of haplotypes for each location was also provided in a proper map, to show the clear distributions of haplotypes and genetic connectivity among the populations under study.

Results

Genetic Diversity

All sequences of *S. lewini* obtained in this study were deposited in the BOLD System with the Barcode Index Number (BIN) registry of BOLD:AAA2403 and database of GenBank with accession numbers MT324149-248 (Table 2). A total of 179 sequences of the 594 bp of the mitochondrial *CO1* gene of *S. lewini* obtained from three sampling sites (Aceh, Balikpapan, and Lombok), and additional sequences ofof samples from West Papua and the West Indian Ocean region have were used to generated a total of 11 haplotype variations with 40 polymorphic loci (Table 3). All the sequences of *S. lewini* obtained from in this study were deposited in the BOLD System with the Barcode Index Number (BIN) registry of BOLD:AAA2403.

Formatted: Font: Times New Roman, 12 pt, English (United States)

Table 3. Forty polymorphic loci of 11 haplotypes from 179 sequences of the mitochondrial ~~*COI*~~ *CO1* gene of *S. lewini* samples from four localities in Indonesia and three localities in the Western Indian Ocean region

Haplotypes	Locus Position																																							
	**1**	**15**	**24**	**27**	**33**	**45**	**48**	**63**	**69**	**105**	**120**	**138**	**141**	**156**	**165**	**174**	**186**	**198**	**229**	**279**	**289**	**303**	**306**	**325**	**339**	**342**	**346**	**366**	**396**	**420**	**453**	**456**	**459**	**483**	**495**	**498**	**522**	**531**	**534**	**543**
H1*	A	T	A	C	T	T	G	T	T	A	T	C	G	T	T	C	C	T	T	C	G	T	T	C	C	A	C	T	T	C	C	T	T	C	T	C	C	T	C	T
H2*	.	.	.	A	.	.	.	.	.	G	.	A	T	.	C	T	T	C	C	T	.	C	C	T	T	C	.	C	C	T	.	.	.	A	.	T	.	C	.	C
H3	.	.	.	A	.	.	.	.	C	G	.	A	T	.	C	T	T	C	C	T	.	C	.	T	T	C	.	C	C	T	.	.	.	A	.	T	.	C	.	.
H4*	.	.	.	A	.	.	.	.	.	G	.	A	T	.	C	T	T	C	C	T	.	C	C	T	T	C	.	C	C	T	.	.	.	A	.	T	.	C	.	.
H5	.	.	.	.	.	.	.	.	.	.	.	.	.	.	.	.	.	.	.	.	.	.	.	.	.	.	.	.	.	.	.	.	G	.	G	.	.	.	.	.
H6	.	.	.	.	.	C	.	.	.	.	.	.	.	.	.	.	.	.	.	.	.	.	.	.	.	.	.	.	.	.	.	.	.	.	.	.	.	.	.	.
H7	.	.	C	.	.	.	.	.	.	.	.	.	.	G	.	.	.	.	.	.	.	.	.	.	.	.	.	.	.	.	.	.	.	.	.	.	.	.	.	.
H8	.	C	.	.	C	.	.	.	.	.	.	.	.	.	.	.	.	.	.	.	.	.	.	.	.	.	.	.	.	.	.	.	.	.	.	.	.	.	.	.
H9	.	.	.	.	.	.	.	.	.	.	G	.	.	.	.	.	.	G	.		T	.	.	.	.	.	.	.	.	.	.	.	.	.	.	.	.	.	G	.
H10	G	.	.	.	.	.	.	G	.	.	.	.	.	G	.	.	.	.	.	.	.	.	.	.	.	.	.	.	.	.	.	.	.	.	.	.	.	.	.	.
H11	.	.	.	.	.	.	.	.	.	.	.	.	.	.	.	.	.	.	.	.	.	.	.	.	.	.	T	.	.	.	T	.	.	.	C	.	T	.	.	.

Notes:

Nucleobase at each position is given for H1 and only nucleobases that were different from H1 are written for all other haplotypes. ~~Identical nucleobase~~ Nucleobases identical to H1 are indicated with ~~periods~~ dots (.)

*Three original haplotypes of the *S. lewini* populations from the ~~3~~ three areas of study (Aceh, Balikpapan, and Lombok) in Indonesia. ~~Other~~ The remaining haplotypes were reanalyzed from previous studies.

Commented [Editor3]: Remark: We have moved table 3 here before table 4.

Commented [SH4R3]: Thank you for your correction.

Comparison The comparison of the genetic diversity of *S. lewini* based on haplotype and nucleotide diversity revealed the presence of varied variationin value (Table 4). The haplotype diversity (h) among the localities in Indonesia ranged from 0.143 to -0.668 and the nucleotide diversity (π) ranged from 0.0001 to -0.020. The highest value of genetic diversity in Indonesia was shown byobserved for the population in from Aceh (h = 0.668; π = 0.020), followed by the Balikpapan population, which was exhibited a lower in haplotype and nucleotide diversity (h = 0.646; π = 0.002), whereas and the lowest genetic diversity was found detected in West Papua (h = 0.143, π = 0.0001). Population ofThe *S. lewini* population fromin Lombok also had exhibited a fairly low genetic diversity value (h = 0.362; π = 0.001). However, the overall genetic diversity in Indonesia was relatively high in value (h = 0.717; π = 0.013). On the other handConversely, the averagevalue of genetic diversity of the Western Indian Ocean region was low in average and ranged between from 0.000 to -0.467. The overall diversity in the Western Indian Ocean region was also low in value (h = 0.232; π = 0.001).

Table 4. Genetic diversity of *S. lewini* based on sample size (n), haplotype number (Hn), haplotype diversity (h), and nucleotide diversity (π) in samples from each ~~population~~ site in Indonesia and the Western Indian Ocean region

Population	n	Genetic Diversity		
		**Hn**	**h**	**π**
**Indonesia**				
Aceh (ACH)	41	4	0.668	0.020
Balikpapan (BPN)	30	3	0.646	0.002
Lombok (LOM)	29	3	0.362	0.001
West Papua (WEP)	14	2	0.143	0.000~~1~~
Overall Indonesia	114	4	0.717	0.013
**Western Indian Ocean**				
India (IND)	6	1	0.000	0.000
United Arab Emirates (UAE)	30	8	0.467	0.002
Madagascar (MDG)	29	1	0.000	0.000
Overall Western Indian Ocean	65	8	0.232	0.001

Population Structure

The analysis of the fixation index (F_{ST}) and the corresponding ~~significant~~ *P*-values between and within the four *S. lewini* populations (ACH, BPN, LOM, and WEP) ~~in~~ from Indonesia and the three populations (IND, UAE, and MDG) from the Western Indian Ocean region are~~were~~ shown in Table 5. The overall ~~value of~~ F_{ST} value in Indonesia was significantly higher ~~and significant~~than that observed in other regions (F_{ST} = 0.442; ~~*p*~~ *P* < 0.001), which indicated ~~that there were~~the presence of multiple subdivisions in Indonesia.

~~Low~~ A low F_{ST} value ~~and~~ with no significant ~~different~~ differences was ~~exhibited~~ observed in the~~by~~ population from the Western Indian Ocean region (F_{ST} = ~~–~~–0.013; ~~*p*~~ *P* = 0.601), while a ~~comparative~~ comparison of the population structure between Indonesia and the Western Indian Ocean region ~~showed~~ yielded significant ~~differentiation~~ differences (F_{ST} = 0.740; ~~*p*~~ *P* < 0.001). The pairwise F_{ST} values between the populations ~~in~~ from the four locations in Indonesia and a comparison with those from Western Indian Ocean population ~~was~~ are shown in Table 6. The overall pairwise analysis ~~by~~ via the distance method indicated the presence of significant ~~differentiation~~ differences among the four populations ~~in~~ from Indonesia, with the exception of ~~between~~ BPN and LOM, which showed fairly low F_{ST} values and no significant p values. Furthermore, among the populations ~~in~~ from Indonesia, the ACH population ~~was tent~~ tended to be closer to the Western Indian Ocean~~WIO~~ population, as it exhibited a ~~with~~ lower F_{ST} value (rather than F_{ST} range) ~~value~~ ~~of~~ compared with other localities ~~in~~ from Indonesia to the Western Indian Ocean.

Table 5. Analysis of molecular variance (AMOVA) for percentage of variation (%), F_{ST} value, and ~~significant~~ significance level (~~*p*~~*P*-value) ~~of~~ in *S. lewini* samples ~~for~~ from Indonesian populations, Western Indian Ocean populations, and between Indonesian and Western Indian Ocean populations

Source of variation	df	Percentage of variation (%)	F_{ST} value	~~*p*~~ *P* value
**Indonesia**				
Among Populations	3	44.15	0.442	0.000
Within Populations	110	55.85		
Total	113			
**Western Indian Ocean**				
Among Populations	2	~~-~~–1.31	~~-~~–0.013	0.601

Within Populations	62	101.31		
Total	64			
**Indonesia vs. Western Indian Ocean**				
Among Populations	1	74.04	0.740	0.000
Within Populations	177	25.96		
**Total**	**178**			

Table 6. Pairwise F_{ST} values (below the diagonal) and *P*-values (above the diagonal) between the *S. lewini* populations from ~~in~~ Aceh (ACH), Balikpapan (BPN), Lombok (LOM), West Papua (WEP), and Western Indian Ocean (WIO)

Sample Sites	ACH	BPN	LOM	WEP	WIO
**ACH**	-	0.000	0.000	0.000	0.000
**BPN**	0.427	-	0.091~~0~~	0.001	0.000
**LOM**	0.438	0.044	-	0.000	0.000
**WEP**	0.398	0.495	0.736	-	0.000
**WIO**	0.509	0.965	0.973	0.975	-

Genetic Connectivity

~~There were~~A haplotype network analysis identified two main groups of haplotypes ~~that were shown by haplotype network analysis~~ (Fig. 1), ~~hereafter~~ referred to as Clade A and Clade B henceforth. Clade A consisted of haplotype H1, ~~found~~ which was observed in ~~multiple~~ several regions, i.e., ~~in~~ Aceh, India, United Arab Emirates, and Madagascar, ~~while~~ whereas H5, H6, H7, H8, H9, H10, and H11 were ~~originated~~ present only in the samples from the United Arab Emirates. Clade B consisted of three haplotypes (H2, H3, and H4), which were spread evenly in Indonesia, although each region exhibited~~there were~~ predominant haplotypes ~~found in every single region~~. Haplotypes H1, H2, H4, and H3 were predominant in Aceh, Balikpapan, Lombok, and West Papua, ~~was predominated by H1, H2, H4, and H3~~ respectively (Fig. 2).

Fig 1. Haplotype network of the *S. lewini* (n = 179) population ~~in~~ from Indonesia and the Western Indian Ocean region, which was constructed ~~with~~ using the Median Joining method

Fig 2. Distribution of the 11 haplotypes of the *S. lewini* population from Indonesia and Western Indian Ocean ~~in~~ at the regional scale

Discussion

Genetic Diversity

The overall genetic diversity ~~both~~ of *S. lewini* at the haplotype and nucleotide levels ~~of *S. lewini*~~ was relatively high for populations in Indonesia. These findings are consistent with the results reported by ~~of~~ Ovenden *et al.* [14], ~~with~~ pertaining to the mitochondria control region of *S. lewini* from three localities in Indonesia, including Lombok, and the high genetic diversity of *S. lewini* observed at their~~in~~ global scale [13]. The scalloped hammerhead sharks are a highly migratory species with a wide distribution in tropical and warm-temperate waters and possibly move across oceanic waters to a distance of up to 1500 km ~~far~~ [9]. In principle, ~~with~~ because of migration capability and broad ecological niches, this species tends to have a higher genetic diversity than do species from narrow ecological niches ~~species~~ [30]. High genetic diversity values are commonly related to large population sizes [31]~~,~~ and ~~also~~ are promoted by ~~some~~ several factors, such as local population sizes, fast generation~~s~~ times [32], high nucleotide substitution rates [33], and ~~the~~ high gene flow between geographically distant populations.

The finding of a relatively high genetic diversity ~~of~~ for *S. lewini* in Indonesia appears to be inconsistent with the general assumption that overexploitation of this species as both

Commented [Editor5]: Remark: Confirm with the target journal whether the figure legends have to be placed here.

Commented [SH6R5]: Thank you for your remark. I have rechecked the format and those were in accordance with targeted journal guidelines.

a target of fishing and bycatch ~~have~~ has led to the decline~~d the scalloped hammerhead shark~~ of its populations worldwide [34].~~,~~ ~~however~~However, the results obtained here for the population from Lombok~~this could~~ may be relevant in this context~~;with the fact occurred in Lombok.~~ i.e., the ~~Lower~~ lower genetic diversity ~~evidence occurred in Lombok~~detected at that location was probably driven by continuous fishing pressure. *S. lewini* were the top three targeted sharks ~~landed in~~at the Tanjung Luar fishing port in~~,~~ Lombok and have faced high fishing pressure over more than 40 years, ~~that~~ which has ~~lead~~ led to its overfishing, with recent exploitation rates (E) ~~that reached~~ reaching 0.59~~, recently~~ [35, ~~-~~36].

Furthermore, according to ~~fisheries~~ the global information system statistic of the FAO on fisheries [37], the hammerhead sharks (Sphyrnidae), including *S. lewini*, are ~~one of the~~ important sharks ~~which~~ that were highly exploited in the last two decades, ~~which~~ with an estimated rapid~~ly~~ increase in global capture, ~~since~~ from 220 ton per year in 1985~~, reaching~~ up to approximately 10,362 tons per year in 2016. In the same period, the capture level in Indonesia also increased significantly~~ increase~~, reaching approximately 1,492 tons in 2016.~~,~~ ~~with the fact that~~Moreover, Indonesia ~~was in~~exhibited one of the highest volumes of sharks and rays caught on the global catches reported in the period of 2000~~-~~–2011 [38]. Clarke *et al.* [39] reported similar findings regarding ~~global~~ the mitochondrial DNA of the silky shark, *Carcharhinus falciformis*, ~~with~~ which exhibited a high genetic diversity under circumstances of overexploitation. Elasmobranchs ~~have the~~exhibit adaptability ~~with~~ to environmental and anthropogenic stresses, which causes genetic bottlenecks because of their particular life histories [40]. Moreover, the population decline caused by recent fishery activities ~~recently~~ might be insufficient ~~in time~~ to reduce genetic diversity,

particularly for species with a long life ~~species~~ span (13–20 years), including *S. lewini* [41, ~~–~~42].

However, ~~indeed~~ the continuous ~~declining~~ decline in~~large~~ these populations~~ continuously~~, as was expected to occur ~~with~~ for the *S. lewini* population ~~in~~ from Lombok, ~~had~~ was positively ~~correlation~~ correlated with the loss of genetic diversity and ~~driven~~ created a population bottleneck [42, ~~–~~43], as ~~revealed~~ reported by Pinsky and Palumbi [44] in a meta-analysis of several ~~some~~ marine fish ~~meta-analysis~~.

Population Structure

Our findings indicated the presence of homogeneity among the *S. lewini* populations from Balikpapan and Lombok. ~~Pairwise~~ The pairwise F_{ST} analysis detected no significant genetic differentiation in these two populations with lowest value of F_{ST}~~contiguous spatial distant~~. ~~This finding complements~~These finding complements ~~with~~ those of previous studies conducted in Indo-Australian waters. Ovenden *et al.* [14] reported similar evidence for the mitochondrial control region ~~on~~ regarding population structure, with no differentiation ~~for~~ between two populations of *S. lewini* ~~between Indonesian population including~~ (Lombok and northern Australia). This pattern of ~~single-~~single-stock population seems to suggest that these localities are a migration zone of *S. lewini* and that a reproductive movement might occur in the coastal areas of these locations, although there are strong Indonesian throughflow currents between Kalimantan and Sulawesi island. Adult~~s~~ *S. lewini* specimens are highly migratory, with a large body size that supports ~~of~~ the high dispersal capacity of this species; ~~in~~ hence, they exhibit the ~~possibly~~ possibility of~~to~~ ~~control~~ overcoming that geographical barrier. ~~Otherwise~~In contrast, we

Commented [Editor7]: Remark: This is unclear. Kindly consider rewriting this phrase for clarity.

Commented [SH8R7]: Thank you for your correction. I have tried to rewrite the phrase.

~~observed~~ obtained different results ~~with~~ from the comparison among these two populations ~~in~~ from central Indonesia~~,~~ ~~with~~ and one population ~~in~~ from Aceh (western Indonesia) and one population from West Papua (eastern Indonesia), which exhibited strong genetic differentiation. These regions were spatially separated by a long distance, with the possibility of the existence of more complex barriers, both ~~inter~~ inter-island barriers and anthropogenic factors ~~of~~ pertaining to a high commercial and artisanal fishing pressure, such as those found along the southern and northern coasts of Java.

Genetic Connectivity

We ~~found~~ obtained an interesting result regarding haplotype sharing between the populations ~~in~~ from Indonesia and the Indian Ocean population. H1 was ~~the~~ a unique haplotype found in Aceh, Indonesia, and ~~this haplotype~~ was correlated with the most common haplotype ~~in~~ observed in samples from the three locations of the ~~western~~ Western Indian Ocean region ~~from~~ (India, United Arab Emirates, and Madagascar), which ~~could indicated~~ indicates ~~that there was~~the presence of connectivity between the populations from Aceh and those from the ~~western~~ Western Indian Ocean region. Schott and McCreary [45] ~~described~~ reported that the Indian Ocean seasonal current carried water from north ~~Sumatera~~ Sumatra through the Arabian Sea to Madagascar, which ~~was~~ represents a possible ~~as~~ connectivity channel ~~of~~ between the two regions, ~~with~~ thus allowing *S. lewini* to migrate and leading to ~~cross~~ cross-breeding. ~~Similar~~ The similarity between the predominant haplotype (H1) of *S. lewini* from Aceh with that of the populations from India, United Arab Emirates, and Madagascar ~~were~~ evidenced a ~~of~~ genetic sharing process ~~between~~ among populations in the Indian Ocean region. However, the *S. lewini* populations from Balikpapan, Lombok, and West Papua ~~was~~

seemed to be~~indicated~~ ~~isolation~~ isolated from the population ~~in~~ from the ~~western~~ Western Indian Ocean, and shared a haplotype network ~~only~~ exclusively in eastern Indonesian waters.

Conservation Implications

The overall high ~~value of~~ genetic diversity of the *S. lewini* populations in Indonesia suggests that this species has not yet experienced a genetic loss ~~due to~~because of exploitation pressure. However, the lower genetic diversity of *S. lewini* ~~in~~ from Lombok and West Papua indicated a higher risk of loss of genetic diversity, ~~loss that~~ which probably was the result of high fisheries pressure. Our genetic assessment of *S. lewini* samples from four localities revealed that a single stock exist between Lombok and Balikpapan, ~~and~~ while a separate~~d~~ stock was observed for Aceh and West Papua, indicating that the management of this species should occur on a stock-based approach at least on three mitochondrial-stock conservation management units. ~~A~~ The complex ~~and limited connectivity~~ population of *S. lewini* with limited connectivity ~~that existed~~observed in ~~population in~~ Indonesia~~,~~ and ~~between~~ the Western Indian Ocean region ~~indicating~~ indicated the importance ~~to~~ of ~~promote~~ promoting collaborative management ~~strategic~~ strategies ~~between~~ among Indonesian management agencies~~,~~ and ~~also~~ in conjunction with Western Indian Ocean agencies ~~in~~ at the regional scale~~s~~.

Conclusion

This study ~~has shown~~provided important findings regarding the population structure of *S. lewini* in Indonesia, with a high genetic diversity and three significant subdivisions that indicated the capability of the population to adapt to rapid environmental changes and

pressure, including fishing activities. However, the lower genetic ~~values~~ diversity detected in Lombok and West Papua should ~~still~~ be considered. ~~Genetic~~ The genetic sharing ~~which were~~ detected ~~between~~ among the populations ~~in~~ from Indonesia and also those from the Western Indian Ocean~~,~~ proved the connectivity ~~between~~ among these populations ~~in Indonesia and these are also connected with the Western Indian Ocean population~~. ~~Collaborative~~ A collaborative action across regions is needed to promote sustainable management and conservation purposes, both in Indonesia and at the regional scale in the Western Indian Ocean area~~, in regional scale~~.

Acknowledgement

We would like to thank to the institutions and individuals that have made our study possible: colleagues from Marine Biodiversity and Biosystematics Lab (BIODIVSI), Faculty of Marine Science and Technology, IPB University for their help during research. We also appreciated to BPSPL Satker Balikpapan, Listian Nova and Mr. Hery, Head of ~~PPI~~ TPI Manggar Balikpapan for their help during collecting samples, and to reviewers and proof readers who have provided highly constructive suggestions for better writing of the paper.

References

1. Klimley AP, Butler SB. Immigration and emigration of a pelagic fish assemblage to seamounts in the Gulf of California related to water mass movements using satellite imagery. Mar Ecol Prog Ser. 1988;49: 11-20.

2. Compagno LJV. FAO Species Catalogue: Sharks of the world. Rome: Food And Agriculture Organization;1984.
3. Montgomery JC, Walker MM. Orientation and navigation in elasmobranchs: which way forward? Environ Biol Fishes. 2001;60: 109-116.
4. Kajiura SM, Holland KN. Electroreception in juvenile scalloped hammerhead and sandbar sharks. J Exp Biol. 2002;205: 3609-3621.
5. Meyer CL, Holland KN, Papastamatiou YP. Sharks can detect changes in the geomagnetic field. J R Soc Interface. 2005;2: 129-130.
6. Daly-Engel TS, Seraphin KD, Holland KN, Coffey JP, Nance HA, Toonen RJ, et al. Global phylogeography with mixed-marker analysis reveals male-mediated dispersal in the endangered scalloped hammerhead shark (*Sphyrna lewini*). PLoS ONE. 2012;7(1): e29986. doi: 10.1371/journal.pone.0029986.
7. Castillo-olguin E, Aribe-Alcocer M, Diaz-Jaimes P. Assessment of the population genetic structure of *Sphyrna lewini* to identify conservation units in the Mexican Pacific. Ciencias Marinas. 2012;38(4): 635-652. doi: 10.7773/cm.v38i4.2110.
8. Ward RD. Genetics in fisheries management. Hidrobiologia. 2000;420: 191–201.
9. Kohler NE, Turner PA. Shark tagging: a review of conventional methods and studies. Environ Biol Fishes. 2001;60: 191-223.
10. Bessudo S, Soler GA, Kimley PA, Ketchum J, Arauz R, Hearn A, et al. Vertical and horizontal movements of scalloped hamemerhead shark (*Sphyrna lewini*) around Malpelo and Cocos islands (Tropical Eastern Pacific) using satellite telemetry. Boletín de Investigaciones Marinas y Costeras. 2011;40: 91-106.
11. Cardeñosa D, Hyde J, Caballero S. Genetic diversity and population structure of the pelagic thresher shark (*Alopias pelagicus*) in the Pacific Ocean: evidence for two

evolutionarily significant units. PLoS ONE. 2014;9(10): e110193. doi: 10.1371/journal.pone.0110193.

12. Quintanilla S, Gómez A, Mariño-Ramírez C, Sorzano C, Bessudo S, Soler G, et al. Conservation genetics of the scalloped hammerhead shark in the Pacific Coast of Colombia. J Hered. 2015;106: 448-458. doi:10.1093/jhered/esv050.
13. Duncan KM, Martin AP, Bowen BW, De Coute HG. Global phylogeography of the scalloped hammerhead shark (Sphyrna lewini). Mol Ecol. 2006;15:2239-51.
14. Ovenden JR, Kashiwagi T, Broderick D, Giles J, Salini J. The extent of population genetic subdivision differs among four co-distributed shark species in the Indo-Australian archipelago. BMC Evol Biol. 2009;9: 40. doi:10.1186/1471-2148-9-40.
15. Baldwin CC, Mounts JH, Smith DG, Weigt LA. Genetic identification and color descriptions of early life-history stages of Belizean *Phaeoptyx* and *Astrapogon* (Teleostei: Apogonidae) with comments on identification of adult *Phaeoptyx*. Zootaxa. 2009;2008: 1-22.
16. Madduppa H, Ayuningtyas RU, Subhan B, Arafat D. Exploited but unevaluated: DNA barcoding reveals skates and stingrays (Chordata, Chondrichthyes) species landed in the Indonesian fish market. IJMS. 2016;21: 77-84.
17. Prehadi, Sembiring A, Kurniasih EM, Rahmad, Arafat D, Subhan B et al. DNA barcoding and phylogenetic reconstruction of shark species landed in Muncar fisheries landing site in comparison with Southern Java fishing port. Biodiversitas. 2015;16: 55-61.
18. Hadi S, Anggraini NP, Muttaqin E, Simeon BM, Subhan B, Madduppa H. Genetic diversity of the endangered species Sphyrna lewini (Griffith and Smith 1834) in

Lombok based on mitochondrial DNA. IOP Conf. Ser.: Earth Environ. Sci. 2019;236: 012024.

19. Tamura K, Stecher G, Peterson D, Filipski A, Kumar S. Molecular Evolutionary Genetics Analysis Version 6.0. Mol Biol Evol. 2013;30(12): 2725-2729. doi: 10.1093/molbev/mst197.

20. Rozas J, Ferrer-Mata A, Sánchez-DelBarriol JC, Guirao-Rico S, Librado P, Ramos-Onsins SE. DnaSP v6: DNA Sequence polymorphism analysis of large datasets. Mol Biol Evol. 2017;34: 3299-3302.

21. Excoffier L, H.E. L. Lischer. Arlequin suite ver 3.5: A new series of programs to perform population genetics analyses under Linux and Windows. Mol Ecol Resour. 2010;10: 564-567.

22. Sembiring A, Pertiwi NPD, Mahardini A, Wulandari R, Kurniasih EM, Kuncoro AW, et al. DNA barcoding reveals targeted fisheries for endangered sharks in Indonesia. Fisheries Research. 2015;164: 130-134. doi: 10.1016/j.fishres.2014.11.003.

23. Bineesh KK, Gopalakrishnan A, Akhilesh KV, Sajeela KA, Abdussamad EM, Pillai NGK, et al. DNA barcoding reveals species composition of sharks and rays in the Indian commercial fishery. Mitochondrial DNA. 2016. doi: 10.3109/19401736.2015.1137900.

24. Jabado RW, Ghais SMA, Hamza W, Henderson AC, Spaet JLY, Shivji MS, et al. The trade in sharks and their products in the United Arab Emirates. Biol Conserv. 2015;181: 190-198. doi: 10.1016/j.biocon.2014.10.032.

25. Doukakis P, Hanner R, Shivji M, Bartholomew C, Chapman D, Wong E, et al. Applying Genetic Techniques to Study Remote Shark Fisheries in Northeastern Madagascar. Mitochondrial DNA. 2011;22(S1): 15-20.
26. Wright S. The genetical structure of populations. Annals of Eugenics. 1951;15: 323-354.
27. Wright S. Evolution and the Genetics of Populations. 4th vol. Variability within and among Natural Populations. Chicago: University of Chicago Press; 1978.
28. Hartl DL, Clark AG. Principles of Population Genetics. 3nd ed. Sunderland: Sinauer Associates Inc; 1997.
29. Bandelt H-J, Forster P, Röhl A. Median-joining networks for inferring intraspecific phylogenies. Mol Biol Evol. 1999;16: 37-48.
30. Habel JC, Schmitt T. Vanishing of the common species: Empty habitats and the role of genetic diversity. Biol. Conserv. 1996;218: 211-216. doi: 10.1016/j.biocon.2017.12.018.
31. Frankham R. Relationship of Genetic Variation to Population Size in Wildlife. Conserv. Biol. 1996;10(6): 1500-1508. doi:10.1046/j.1523-1739.1996.10061500.x.
32. Hague MTJ, Routman EJ. Does population size affect genetic diversity? A test with sympatric lizard species. Heredity. 2016;116: 92-98. doi: 10.1038/hdy.2015.76.
33. Martin AP, Naylor GJP, Palumbi SR. Rates of mitochondrial DNA evolution in sharks are slow compared with mammals. Nature. 1992;357: 153-157. doi: 10.1038/357153a0.
34. Compagno LJV, Dando M, Fowler SL. Sharks of the World. Princeton: Princeton University Press; 2005.

35. Hordyk A, Ono K, Valencia S, Loneragan N, Prince J. A novel length-based empirical estimation method of spawning potential ratio (SPR), and tests of its performance, for small-scale, data-poor fisheries. ICES J Mar Sci. 2015;2-15. doi: 10.1093/icesjms/fsu004.
36. Simeon BM, Agustina S, Muttaqin E, Yulianto I, Ichsan M, Muhsin M. Laporan teknis: Pemanfaatan Hasil Tangkap Hiu dan Pari di Tanjung Luar, Provinsi Nusa Tenggara Barat. Bogor: Wildlife Conservation Society-Indonesia Program; 2017.
37. Food and Agriculture Organization (FAO). Fisheries and Aquaculture Information and Statistics Service: Global Capture Production 1950-2017; 2017 [cited 2020 Mar 5]. Database: Indonesian hammerhead shark capture [Internet]. http://www.fao.org/fishery/statistics/global-capture-production/query/es.
38. Dent F, Clarke. State of the Global Market for Shark Products. Rome: Food And Agriculture Organization; 2015.
39. Clarke CR, Karl SA, Horn RL, Bernard AM, Lea JS, Hazin FH et al. Global mitochondrial DNA phylogeography and population structure of the silky shark, *Carcharhinus falciformis.* Mar Biol. 2015;162(5): 945-955. doi: 10.1007/s00227-015-2636-6
40. Chapman DD, Simpfendorfer CA, Wiley TR, Poulakis GR, Curtis C, Tringali M et al. Genetic diversity despite population collapse in a critically endangered marine fish: the smalltooth sawfish (*Pristis pectinata*). J Hered. 2011;102: 643-652.
41. Sánchez-de Ita JA, Quiñónez-Velázquez C, Galván-Magaña F, Bocanegra-Castillo N, Félix-Uraga R. Age and growth of the silky shark *Carcharhinus falciformis* from the west coast of Baja California Sur, Mexico. J App Ichthyol. 2011;27: 20-24.

42. Nei M, Maruyama T, Chakraborty R. The bottleneck effect and genetic variability in populations. Evolution. 1975;29(1): 1. doi:10.2307/2407137.

43. Beebee T, Rowe G. An Introduction to Molecular Ecology. 2nd ed. New York: Oxford University Press Inc; 2008.

44. Pinsky ML, Palumbi SR. Meta-analysis reveals lower genetic diversity in overfished populations. Mol Ecol. 2013;23(1): 29-39. doi:10.1111/mec.12509.

45. Schott FA, McCreary JP. The monsoon circulation of the Indian Ocean. Prog Oceanogr. 2001;51: 1-123. doi: 10.1016/S0079-6611(01)00083-0.

Dear Susana Caballero, PhD
Academic Editor
PLOS ONE

Thank you for reviewing and suggesting our manuscript:

"Genetic connectivity and diversity of endangered species the scalloped hammerhead shark Sphyrna lewini (Griffith & Smith 1834) population across Indonesia and the Western Indian Ocean"

I would like to response your comments:

Additional Editor Comments (if provided):

This is a very interesting manuscript and it present very important information that will help understanding the genetic diversity of scalloped hammerhead in this region and this information will be likely used for future assignment comparisons for example for market surveys etc. However, the English NEEDS to be improved. I really hope the authors can find the help of a native English speaker that can help them with this. I wrote some things in the PDF I am attaching but it definitely needs an overall improvement of the English
.

Thank you for your interest and suggestion. We have improved overall of the English for better manuscript after proof read by professional native English speaker.

The resolution of the figures needs to be improved because they look grainy.

Thank you for your suggestion. We have improved the resolution of figures and generated in PACE to meet PLOS ONE requirement.

We would appreciate receiving your revised manuscript by 20th of April. When you are ready to submit your revision, log on to https://www.editorialmanager.com/pone/ and select the 'Submissions Needing Revision' folder to locate your manuscript file.
If you would like to make changes to your financial disclosure, please include your updated statement in your cover letter.

To enhance the reproducibility of your results, we recommend that if applicable you deposit your laboratory protocols in protocols.io, where a protocol can be assigned its own identifier (DOI) such that it can be cited independently in the future. For instructions see: ttp://journals.plos.org/plosone/s/submission-guidelines#loc laboratory-protocols

Please include the following items when submitting your revised manuscript:

- A rebuttal letter that responds to each point raised by the academic editor and reviewer(s). This letter should be uploaded as separate file and labelled **'Response to Reviewers'**.
- A marked-up copy of your manuscript that highlights changes made to the original version. This file should be uploaded as separate file and labelled **'Revised Manuscript with Track Changes'**.
  •An unmarked version of your revised paper without tracked changes. This file should be uploaded as separate file and labelled **'Manuscript'**.

Journal Requirements:

When submitting your revision, we need you to address these additional requirements:

1. Please ensure that your manuscript meets PLOS ONE's style requirements, including those for file naming. The PLOS ONE style templates can be found at http://www.plosone.org/attachments/PLOSOne_formatting_sample_main_body.pdf and http://www.plosone.org/attachments/PLOSOne_formatting_sample_title_authors_affiliations.pdf

   Thank you for your suggestion. We have read and followed the format as The PLOS ONE style templates.

2. In your Methods section, please provide additional location information of the sampling collection sites, including geographic coordinates for the data set if available.

   Thank you for your suggestion. We have provided additional location information including geographic coordinates in a table in manuscript

3. We suggest you thoroughly copyedit your manuscript for language usage, spelling, and grammar. If you do not know anyone who can help you do this, you may wish to consider employing a professional scientific editing service.

   Thank you for your suggestion. We have sent and proof read our manuscript to a professional scientific editor to check and enhance our manuscript grammar, spelling and language use.

Upon resubmission, please provide the following:

- The name of the colleague or the details of the professional service that edited your manuscript
- A copy of your manuscript showing your changes by either highlighting them or using track changes (uploaded as a *supporting information* file)
- A clean copy of the edited manuscript (uploaded as the new *manuscript* file)

4. Thank you for stating the following in the Acknowledgments Section of your manuscript:
   "This research was supported by Wildlife Conservation Society (WCS)-Indonesia, in collaboration with Faculty of Fisheries and Marine Sciences IPB University. Sutanto Hadi received a scholarship from Indonesia Endowment Fund for Education (LPDP)."

   We note that you have provided funding information that is not currently declared in your Funding Statement. However, funding information should not appear in the Acknowledgments section or other areas of your manuscript. We will only publish funding information present in the Funding Statement section of the online submission form.

   Please remove any funding-related text from the manuscript and let us know how you would like to update your Funding Statement. Currently, your Funding Statement reads as follows:
   "NO - The funders had no role in study design, data collection and analysis, decision to publish, or preparation of the manuscript."

   Thank you for your suggestion. We have removed any funding-related statement from the manuscript.

5. PLOS requires an ORCID iD for the corresponding author in Editorial Manager on papers submitted after December 6th, 2016. Please ensure that you have an ORCID iD and that it is validated in Editorial Manager. To do this, go to 'Update my Information' (in the upper left-hand corner of the main menu), and click on the Fetch/Validate link next to the ORCID field. This will take you to the ORCID site and allow you to create a new iD or authenticate a pre-existing iD in Editorial Manager.
   Please see the following video for instructions on linking an ORCID iD to your Editorial Manager account:
   https://www.youtube.com/watch?v=_xcclfuvtxQ
   [Note: HTML markup is below. Please do not edit.]

Reviewers' comments:

[NOTE: If reviewer comments were submitted as an attachment file, they will be attached to this email and accessible via the submission site. Please log into your account, locate the manuscript record, and check for the action link "View Attachments". If this link does not appear, there are no attachment files to be viewed.]

While revising your submission, please upload your figure files to the Preflight Analysis and Conversion Engine (PACE) digital diagnostic tool, https://pacev2.apexcovantage.com/. PACE helps ensure that figures meet PLOS requirements. To use PACE, you must first register as a user. Registration is free. Then, login and navigate to the UPLOAD tab, where you will find detailed instructions on how to use the tool. If you encounter any issues or have any questions when using PACE, please email us at figures@plos.org. Please note that Supporting Information files do not need this step.

Thank you for your suggestion. We have uploaded and adjusted the pictures to PACE to meet PLOS ONE figures requirement. We attached the picture in submission.

Please send them (sequence data) to GenBank as well.

Thank you for your suggestion. We have deposited our all sequences data to Genbank as well with accession number MT324149 to MT324248.
