# Supplementary material for: Genetic connectivity of the scalloped hammerhead shark Sphyrna lewini across Indonesia and the Western Indian Ocean
Source: PLoS One. 2020 Oct 1;15(10):e0230763. doi: 10.1371/journal.pone.0230763 (PMC7529310; doi:10.1371/journal.pone.0230763)
Supplement: Attachment — Submitted filename: PONE-D-20-06557_R3 (1)SCComm.pdf [file pone.0230763.s006.pdf]

PLOS ONE

Genetic connectivity of the scalloped hammerhead shark Sphyrna lewini (Griffith & Smith 1834) population across Indonesia and the Western Indian Ocean

--Manuscript Draft--

**Manuscript Number:**	PONE-D-20-06557R3
**Article Type:**	Research Article
**Full Title:**	Genetic connectivity of the scalloped hammerhead shark Sphyrna lewini (Griffith & Smith 1834) population across Indonesia and the Western Indian Ocean
**Short Title:**	Genetic connectivity of the scalloped hammerhead shark population
**Corresponding Author:**	Hawis Madduppa<br>Institut Pertanian Bogor<br>Bogor, INDONESIA
**Keywords:**	shark fisheries; population genetics; Conservation genetics; coral triangle; genetic connectivity
**Abstract:**	Sphyrna lewini is an endangered species and its populations have been declining worldwide, including in Indonesia, because of overexploitation. However, there is a lack of recent information regarding recent the population structure in this species, to promote its proper management and conservation status in Indonesia. This study aimed to investigate the genetic diversity, population structure, and connectivity of the S. lewini population in Indonesia, i.e., in three major shark landing sites: Aceh (n = 41), Balikpapan (n = 30), and Lombok (n = 29); moreover, additional sequences were retrieved from West Papua (n = 14) and Western Indian Ocean (n = 65) populations. Analyses of the mitochondrial CO1 gene successfully identified a total of 179 sequences of S. lewini, with an average size of 594 bp, and 40 polymorphic loci in four haplotypes for the Indonesian population and in eight haplotypes for the Western Indian Ocean population. The overall values of genetic diversity in Indonesia were high ($h = 0.717$; $\pi = 0.013$), with the highest values recorded in Aceh ($h = 0.668$; $\pi = 0.002$) and the lowest detected in Papua ($h = 0.143$; $\pi = 0.000$), whereas the overall value was fairly low in the Western Indian Ocean ($h = 0.232$; $\pi = 0.001$). AMOVA and FST revealed three significant population subdivisions in Indonesia (FST = 0.442; $P < 0.001$), with separated populations for Aceh and West Papua, and a mixed population between Balikpapan and Lombok (FST = 0.044; P = 0.089); in contrast, a relatively insignificant differentiation was observed within the population of the Western Indian Ocean (FST = –0.013; P = 0.601) and a significant different was detected between the Indonesian and the Western Indian Ocean populations (FST = 0.740; IP < 0.001). The construction of a haplotype network provided evidence of gene flow and haplotype sharing between populations. This study indicated the presence of a complex population of S. lewini with limited connectivity in Indonesia, and in the Western Indian Ocean (at the regional scale), which will require co-management actions across regions.
**Order of Authors:**	Hawis Madduppa, Dr.
	Sutanto Hadi
	Noviar Andayani
	Effin Muttaqin
	Benaya Simeon
	Muhammad Ichsan
	Beginer Subhan
**Opposed Reviewers:**	
**Response to Reviewers:**	To:<br>Susana Caballero, PhD<br>Academic Editor<br>PLOS ONE

Powered by Editorial Manager® and ProduXion Manager® from Aries Systems Corporation

Thank you for reviewing and suggesting our manuscript:

“Genetic connectivity and diversity of endangered species the scalloped hammerhead shark Sphyrna lewini (Griffith & Smith 1834) population across Indonesia and the Western Indian Ocean”

I would like to response your comments in the following systematic table format:

1st SUBMISSION RESPONSE
No.REVIEWER COMMENTAUTHOR RESPONSES
Additional Editor Comments
(if provided)
1.This is a very interesting manuscript and it present very important information that will help understanding the genetic diversity of scalloped hammerhead in this region and this information will be likely used for future assignment comparisons for example for market surveys etc. However, the English NEEDS to be improved. I really hope the authors can find the help of a native English speaker that can help them with this. I wrote some things in the PDF I am attaching but it definitely needs an overall improvement of the English
Thank you for your interest and suggestion. We have improved overall of the English for better manuscript after proof read by professional native English speaker.

2.The resolution of the figures needs to be improved because they look grainyThank you for your suggestion. We have improved the resolution of figures and generated in PACE to meet PLOS ONE requirement.
3.We would appreciate receiving your revised manuscript by 20th of April. When you are ready to submit your revision, log on to https://www.editorialmanager.com/pone/ and select the 'Submissions Needing Revision' folder to locate your manuscript file.
If you would like to make changes to your financial disclosure, please include your updated statement in your cover letter.

4.To enhance the reproducibility of your results, we recommend that if applicable you deposit your laboratory protocols in protocols.io, where a protocol can be assigned its own identifier (DOI) such that it can be cited independently in the future. For instructions see: ttp://journals.plos.org/plosone/s/submission-guidelines#loc laboratory-protocols

5.Please include the following items when submitting your revised manuscript:
•A rebuttal letter that responds to each point raised by the academic editor and reviewer(s). This letter should be uploaded as separate file and labelled 'Response to Reviewers'.
•A marked-up copy of your manuscript that highlights changes made to the original version. This file should be uploaded as separate file and labelled 'Revised Manuscript with Track Changes'.
•An unmarked version of your revised paper without tracked changes. This file should be uploaded as separate file and labelled 'Manuscript'.

No.Journal RequirementsAuthor Responses
1.When submitting your revision, we need you to address these additional requirements:
Please ensure that your manuscript meets PLOS ONE's style requirements, including those for file naming. The PLOS ONE style templates can be found at http://www.plosone.org/attachments/PLOSOne_formatting_sample_main_body.pdf and http://www.plosone.org/attachments/PLOSOne_formatting_sample_title_authors_affiliations.pdf
Thank you for your suggestion. We have read and followed the format as The PLOS ONE style templates.

2.In your Methods section, please provide additional location information of the

	sampling collection sites, including geographic coordinates for the data set if available.<br>Thank you for your suggestion. We have provided additional location information including geographic coordinates in a table in manuscript.<br><br>3.We suggest you thoroughly copyedit your manuscript for language usage, spelling, and grammar. If you do not know anyone who can help you do this, you may wish to consider employing a professional scientific editing service.<br>Thank you for your suggestion. We have sent and proof read our manuscript to a professional scientific editor to check and enhance our manuscript grammar, spelling and language use.<br><br>4.Upon resubmission, please provide the following:<br>•The name of the colleague or the details of the professional service that edited your manuscript<br>•A copy of your manuscript showing your changes by either highlighting them or using track changes (uploaded as a *supporting information* file)<br>•A clean copy of the edited manuscript (uploaded as the new *manuscript* file)<br><br>5.Thank you for stating the following in the Acknowledgments Section of your manuscript:<br>"This research was supported by Wildlife Conservation Society (WCS)-Indonesia, in collaboration with Faculty of Fisheries and Marine Sciences IPB University. Sutanto Hadi received a scholarship from Indonesia Endowment Fund for Education (LPDP)."<br>We note that you have provided funding information that is not currently declared in your Funding Statement. However, funding information should not appear in the Acknowledgments section or other areas of your manuscript. We will only publish funding information present in the Funding Statement section of the online submission form.<br>Please remove any funding-related text from the manuscript and let us know how you would like to update your Funding Statement. Currently, your Funding Statement reads as follows:<br>"NO - The funders had no role in study design, data collection and analysis, decision to publish, or preparation of the manuscript."<br>Thank you for your suggestion. We have removed any funding-related statement from the<br>manuscript.<br><br>6.PLOS requires an ORCID iD for the corresponding author in Editorial Manager on papers submitted after December 6th, 2016. Please ensure that you have an ORCID iD and that it is validated in Editorial Manager. To do this, go to ‘Update my Information’ (in the upper left-hand corner of the main menu), and click on the Fetch/Validate link next to the ORCID field. This will take you to the ORCID site and allow you to create a new iD or authenticate a pre-existing iD in Editorial Manager. Please see the following video for instructions on linking an ORCID iD to your Editorial Manager account:<br>https://www.youtube.com/watch?v=_xcclfuvtxQ<br>[Note: HTML markup is below. Please do not edit.]<br><br>2nd SUBMISSION RESPONSE<br>No.Reviewers' commentsAuthor Responses<br>1.While revising your submission, please upload your figure files to the Preflight Analysis and Conversion Engine (PACE) digital diagnostic tool, https://pacev2.apexcovantage.com/. PACE helps ensure that figures meet PLOS requirements. To use PACE, you must first register as a user. Registration is free. Then, login and navigate to the UPLOAD tab, where you will find detailed instructions on how to use the tool. If you encounter any issues or have any questions when using PACE, please email us at figures@plos.org. Please note that Supporting Information

	files do not need this step.<br>Thank you for your suggestion. We have uploaded and adjusted the pictures to PACE to meet PLOS ONE figures requirement. We attached the picture in submission.<br><br>2.which populations have been declining worldwideThank you for your correction, we have revised the sentence<br>3.use h instead of HdThank you for your correction, we have replaced all of term “Hd” with “h”<br>4.is considered coastal due to their need for nursery areasThank you for your suggestion, we used term “semi-oceanic” as stated in some journals which have the similar meaning that you suggested, so we agreed and have revised the sentence<br><br>5.shorter phrases (Line 47-51)Thank you for your correction. We have shortened the phrases.<br>6.were already dead when collectedThank you for your correction. We have revised the sentence.<br>7.sample (Line 83)Thank you for your correction<br>8.100 samples of which length?<br>Thank you for your question, we used 594 bp of nucleotide and 179 sequences in total, not 100. We have revised it in the sentence<br>9.you previously mention a 100 (sequences)<br>Thank you for your correction. We used 179 sequences in total, we have revised in previous statement<br><br>10.please send them (sequence data) to genbank as wellThank you for your suggestion. We have deposited our all sequences data to Genbank as well with accession number MT324149 to MT324248.<br><br>11.use identifiers particular for your area of study so that they dont get confused with other previously publishedThank you for your suggestion. We used * (asterisk) as identifiers of original haplotype from area of study at notes section below the table.<br>12.repeat “find and finding” (Line 290)<br>Thank you for your correction. We have revised the sentence.<br><br>remove any funding-related text from the manuscriptThank you for your correction. We have removed any funding-related text.<br><br>No.Reviewers' commentsAuthor Responses<br>2nd Submission<br>1.like before: usually 13-16 words,<br>title is too longThank you for your correction. We have tried to shorter the title<br>2.which will make co-management actions necessary across<br>regions (Line 38-39)Thank you for your correction in Line 38-39.<br>3.I think the introduction too short.<br>According to your title you could write some more about the<br>management used now and what are the points of<br>improvement or change in your eyes for S. lewiniThank you for your correction. We have tried to enrich the introduction with what this study can give suggestion for better shark management and conservation<br>What?<br>..additional.. (Line 177)Thank you for your correction in Line 177.<br>What..? were displayed? That’s not a<br>sentence here (Line 181) in TableThank you for your suggestion. We used * (asterisk) as identifiers of original haplotype from area of study at notes section below table.<br>One or more? Than add an sThank you for your correction. We have revised the sentence.<br>One or more? Add sThank you for your correction. We have revised the sentence.<br>What?? Not a sentence here.. (Line 280)Thank you for your correction. We have revised the sentence.<br>Here smaller font size 11 before 12!<br>checkThank you for your correction. We have fixed the font size.<br>Check fond size here again its<br>smaller!! (Line 290,291,302)Thank you for your correction. We have fixed the font size.<br>One or more? Add sThank you for your correction.

Powered by Editorial Manager® and ProduXion Manager® from Aries Systems Corporation

	Strange phrase here..I dont know<br>what that should mean..<br><br>Thank you for your correction. We have revised the sentence.<br>3rd SUBMISSION RESPONSE<br>No.Additional Editor Comments<br>(if provided)Author Responses<br>1.Thank you for submitting your manuscript to PLOS ONE. After careful consideration, we feel that it has merit but does not fully meet PLOS ONE's publication criteria as it currently stands. Therefore, we invite you to submit a revised version of the manuscript that addresses the points raised during the review process.<br>2.The English still needs further improvement and I think the authors need to further analyze their genetic connectivity section. In the discussion, it does not reflect some of the findings you get, particularly from your haplotype network. Please read my suggestions on the attached PDF.Thank you for your correction. We have added some sentences in discussion to reflect our genetic data particularly in connectivity section.<br>3.We would appreciate receiving your revised manuscript by 15th May 2020. When you are ready to submit your revision, log on to https://www.editorialmanager.com/pone/ and select the 'Submissions Needing Revision' folder to locate your manuscript file.<br>4.If you would like to make changes to your financial disclosure, please include your updated statement in your cover letter.<br><br>5.To enhance the reproducibility of your results, we recommend that if applicable you deposit your laboratory protocols in protocols.io, where a protocol can be assigned its own identifier (DOI) such that it can be cited independently in the future. For instructions see: http://journals.plos.org/plosone/s/submission-guidelines#loc-laboratory-protocols<br><br>Laboratory protocols<br>To enhance the reproducibility of your results, we recommend and encourage you to deposit laboratory protocols in protocols.io, where protocols can be assigned their own persistent digital object identifiers (DOIs).<br>To include a link to a protocol in your article:<br>1.Describe your step-by-step protocol on protocols.io<br>2.Select Get DOI to issue your protocol a persistent digital object identifier (DOI)<br>3.Include the DOI link in the Methods section of your manuscript using the following format provided by protocols.io: http://dx.doi.org/10.17504/protocols.io.[PROTOCOL DOI]<br>At this stage, your protocol is only visible to those with the link. This allows editors and reviewers to consult your protocol when evaluating the manuscript. You can make your protocols public at any time by selecting Publish on the protocols.io site. Any referenced protocol(s) will automatically be made public when your article is published.Thank you for your suggestion. We have deposited a short laboratory protocol in protocols.io platform and added the DOI number at the end of Method section, Line 125-127.<br><br>6.Please note while forming your response, if your article is accepted, you may have the opportunity to make the peer review history publicly available. The record will include editor decision letters (with reviews) and your responses to reviewer comments. If eligible, we will contact you to opt in or out.<br><br>Thank you for your suggestion. We have tried to reorganize all comments and our responses in this table to make better history of publication process.<br>No.Reviewers' commentsAuthor Responses<br>1.of a haplotype (typo A)Thank you for your correction. We have fixed it.<br>2.explain how genetic information can be used to trave back the origin of shark find from this species found in markets, particularly in Asia. Cite Fields et al 2020, in Animal Conservation, a manuscript that came out this week.<br>Thank you for your suggestion. We have added introduction in Line 53-67 and cited from Fields et al and Jabado et al in Line 68-75.<br><br>DNA extraction<br>Thank you for your correction. We have fixed it.

Powered by Editorial Manager® and ProduXion Manager® from Aries Systems Corporation

	3.I dont think you need to inlude this, as this is know by population geneticists and also because it will also depend of the probability of significance.<br>Thank you for your suggestion. We agreed with your suggestion, we have omitted the sentence.<br><br>4.No significant differentiation was observed<br>(Line 203)Thank you for your correction. We have revised the sentence.<br><br>5.significant differentiationThank you for your correction. We have revised the phrase.<br><br>6.differentiationThank you for your correction. We have revised it.<br>7.include number of Fst and p value<br>Thank you for your suggestion. We have added value of Fst and p in the sentence Line 210.<br>8.is a highlyThank you for your correction. We have revised the phrase.<br><br>9.what type of fisheries, be more specific<br>Thank you for your suggestion. We have added more specific information in Line 275. “according to the global fisheries information system on hammerhead shark capture production 1950-2017 on global scale and Indonesia by FAO”<br><br>10.you have many nucleotide changes between your haplotypes from Indonesia and those from the Western Indian Ocean...what could be happening there? is there really gene flow with the West Indian Ocean? I think gene flow will be restricted, otherwise you would not find such differentiated haplotypes. Re -think this section and go deeper into your results<br>Thank you for your correction. We have stated some sentences in Line 318-330 regarding haplotype differentiation between two haplogroup between Indonesia and western Indian Ocean, and we also highlight in next sentence 331-347, an indication on genetic sharing between population in Aceh and Western Indian Ocean which have similarity in H1 (haplotype one).<br><br>11.and gene flow<br>Thank you for your correction. We have added the phrase in Line 339.
**Additional Information:**	
**Question**	**Response**
**Financial Disclosure**<br><br>Enter a financial disclosure statement that describes the sources of funding for the work included in this submission. Review the submission guidelines for detailed requirements. View published research articles from *PLOS ONE* for specific examples.<br><br>This statement is required for submission and **will appear in the published article** if the submission is accepted. Please make sure it is accurate.	This research was supported by Wildlife Conservation Society (WCS)-Indonesia, in collaboration with Faculty of Fisheries and Marine Sciences IPB University. SH received a scholarship from Indonesia Endowment Fund for Education (LPDP). The funders had no role in study design, data collection and analysis, decision to publish, or preparation of the manuscript.

Powered by Editorial Manager® and ProduXion Manager® from Aries Systems Corporation

**Unfunded studies**<br>Enter: *The author(s) received no specific funding for this work.*<br><br>**Funded studies**<br>Enter a statement with the following details:<br>• Initials of the authors who received each award<br>• Grant numbers awarded to each author<br>• The full name of each funder<br>• URL of each funder website<br>• Did the sponsors or funders play any role in the study design, data collection and analysis, decision to publish, or preparation of the manuscript?<br>• **NO** - Include this sentence at the end of your statement: *The funders had no role in study design, data collection and analysis, decision to publish, or preparation of the manuscript.*<br>• **YES** - Specify the role(s) played.<br><br>* typeset	
**Competing Interests**<br><br>Use the instructions below to enter a competing interest statement for this submission. On behalf of all authors, disclose any competing interests that could be perceived to bias this work—acknowledging all financial support and any other relevant financial or non-financial competing interests.<br><br>This statement **will appear in the published article** if the submission is accepted. Please make sure it is accurate. View published research articles from *PLOS ONE* for specific examples.	The authors have declared that no competing interests exist.

Powered by Editorial Manager® and ProduXion Manager® from Aries Systems Corporation

**NO authors have competing interests**<br><br>Enter: *The authors have declared that no competing interests exist.*<br><br>**Authors with competing interests**<br><br>Enter competing interest details beginning with this statement:<br><br>*I have read the journal's policy and the authors of this manuscript have the following competing interests: [insert competing interests here]*<br><br>* typeset	
**Ethics Statement**<br><br>Enter an ethics statement for this submission. This statement is required if the study involved:<br><br>• Human participants<br>• Human specimens or tissue<br>• Vertebrate animals or cephalopods<br>• Vertebrate embryos or tissues<br>• Field research<br><br>Write "N/A" if the submission does not require an ethics statement.<br><br>General guidance is provided below. Consult the submission guidelines for detailed instructions. **Make sure that all information entered here is included in the Methods section of the manuscript.**	All specimens were collected in dead condition, no live specimens were obtained. Therefore, no approval from any institutional animal ethics committee was required. The samples collection and transport were in accordance with the regulation of Ministry of Marine Affairs and Fisheries of the Republic of Indonesia Number 5/PERMEN-KP/2018 on the prohibition of cowboy shark and hammerhead shark export from Indonesia. This study was also approved under permission number 276/BPSPL.03/PRL/X/2018 and 319/PNK/BPSPL.03/PK.230/REKOM/X/2018 by Ministry of Marine Affairs and Fisheries of the Republic of Indonesia.

Powered by Editorial Manager® and ProduXion Manager® from Aries Systems Corporation

**Format for specific study types**<br><br>**Human Subject Research (involving human participants and/or tissue)**<br>• Give the name of the institutional review board or ethics committee that approved the study<br>• Include the approval number and/or a statement indicating approval of this research<br>• Indicate the form of consent obtained (written/oral) or the reason that consent was not obtained (e.g. the data were analyzed anonymously)<br><br>**Animal Research (involving vertebrate animals, embryos or tissues)**<br>• Provide the name of the Institutional Animal Care and Use Committee (IACUC) or other relevant ethics board that reviewed the study protocol, and indicate whether they approved this research or granted a formal waiver of ethical approval<br>• Include an approval number if one was obtained<br>• If the study involved *non-human primates*, add *additional details* about animal welfare and steps taken to ameliorate suffering<br>• If anesthesia, euthanasia, or any kind of animal sacrifice is part of the study, include briefly which substances and/or methods were applied<br><br>**Field Research**<br><br>Include the following details if this study involves the collection of plant, animal, or other materials from a natural setting:<br>• Field permit number<br>• Name of the institution or relevant body that granted permission	
**Data Availability**<br><br>Authors are required to make all data underlying the findings described fully available, without restriction, and from the time of publication. PLOS allows rare exceptions to address legal and ethical concerns. See the PLOS Data Policy and FAQ for detailed information.	Yes - all data are fully available without restriction

A Data Availability Statement describing where the data can be found is required at submission. Your answers to this question constitute the Data Availability Statement and **will be published in the article**, if accepted.<br><br>**Important:** Stating 'data available on request from the author' is not sufficient. If your data are only available upon request, select 'No' for the first question and explain your exceptional situation in the text box.<br><br>Do the authors confirm that all data underlying the findings described in their manuscript are fully available without restriction?	
**Describe where the data may be found in full sentences. If you are copying our sample text, replace any instances of XXX with the appropriate details.**<br><br>• If the data are **held or will be held in a public repository**, include URLs, accession numbers or DOIs. If this information will only be available after acceptance, indicate this by ticking the box below. For example: *All XXX files are available from the XXX database (accession number(s) XXX, XXX.).*<br>• If the data are all contained **within the manuscript and/or Supporting Information files**, enter the following: *All relevant data are within the manuscript and its Supporting Information files.*<br>• If neither of these applies but you are able to provide **details of access elsewhere**, with or without limitations, please do so. For example:<br><br>*Data cannot be shared publicly because of [XXX]. Data are available from the XXX Institutional Data Access / Ethics Committee (contact via XXX) for researchers who meet the criteria for access to confidential data.*<br><br>*The data underlying the results presented in the study are available from (include the name of the third party*	All relevant data are within the manuscript. All sequences data have been submitted to BOLD and GenBank and can be access publicly.

Powered by Editorial Manager® and ProduXion Manager® from Aries Systems Corporation

and contact information or URL).<br>• This text is appropriate if the data are owned by a third party and authors do not have permission to share the data.<br><br>* typeset	
Additional data availability information:	Tick here if the URLs/accession numbers/DOIs will be available only after acceptance of the manuscript for publication so that we can ensure their inclusion before publication.

Powered by Editorial Manager® and ProduXion Manager® from Aries Systems Corporation

To:
Susana Caballero, PhD
Academic Editor
PLOS ONE

Thank you for your for reviewing and suggesting our manuscript.

I am enclosing herewith our revised manuscript entitled **"Genetic connectivity of the scalloped hammerhead shark *Sphyrna lewini* (Griffith & Smith 1834) population across Indonesia and Western Indian Ocean"** re-submitted to **"PLOS ONE Journal"** for further evaluation.

Indonesia is one of the main shark fin exporters make this manuscript is very important information that will help understanding the genetic diversity in particular of scalloped hammerhead which evaluated as critical endangered species.

This information will be likely used for future assignment comparisons for example for market surveys, planning shark management and conservation area etc. We provide information in this research to support shark fisheries management and conservation efforts in Indonesia. We believe this manuscript makes an important contribution to the science of assessing and sustainably managing small-scale and data poor fisheries such shark fisheries, globally.

In a separate document we through point by point detailing how we have almost entirely accepted and incorporated their suggestions, and enhancing our manuscript in better.

Corresponding Author

Dr. Hawis Madduppa

Manuscript Click here to access/download;Manuscript;3. Manuscript.docx

Genetic connectivity of the scalloped hammerhead shark *Sphyrna lewini* (Griffith & Smith 1834) population across Indonesia and the Western Indian Ocean

Sutanto Hadi[1], Noviar Andayani[2,3], Effin Muttaqin[3], Benaya M Simeon[3], Muhammad Ichsan[3], Beginer Subhan[1] and Hawis Madduppa[1]*

[1] Department of Marine Science and Technology, Faculty of Fisheries and Marine Sciences, IPB University, Indonesia

[2] Department of Biology, Faculty of Mathematic and Natural Science, University of Indonesia, Depok, Indonesia

[3] Wildlife Conservation Society Indonesia, Bogor, Indonesia

*Corresponding author

Email: hawis@apps.ipb.ac.id

Abstract

Sphyrna lewini is an endangered species and its populations have been declining worldwide, including in Indonesia, because of overexploitation. However, there is a lack of recent information regarding recent the population structure in this species, to promote its proper management and conservation status in Indonesia. This study aimed to investigate the genetic diversity, population structure, and connectivity of the *S. lewini* population in Indonesia, i.e., in three major shark landing sites: Aceh (n = 41), Balikpapan (n = 30), and Lombok (n = 29); moreover, additional sequences were retrieved from West Papua (n = 14) and Western Indian Ocean (n = 65) populations. Analyses of the

mitochondrial *CO1* gene successfully identified a total of 179 sequences of *S. lewini*, with an average size of 594 bp, and 40 polymorphic loci in four haplotypes for the Indonesian population and in eight haplotypes for the Western Indian Ocean population. The overall values of genetic diversity in Indonesia were high (h = 0.717; π = 0.013), with the highest values recorded in Aceh (h = 0.668; π = 0.002) and the lowest detected in Papua (h = 0.143; π = 0.000), whereas the overall value was fairly low in the Western Indian Ocean (h = 0.232; π = 0.001). AMOVA and F_{ST} revealed three significant population subdivisions in Indonesia ($F_{ST} = 0.442$; $P < 0.001$), with separated populations for Aceh and West Papua, and a mixed population between Balikpapan and Lombok ($F_{ST} = 0.044$; $P = 0.089$); in contrast, a relatively insignificant differentiation was observed within the population of the Western Indian Ocean ($F_{ST} = -0.013$; $P = 0.601$) and a significant different was detected between the Indonesian and the Western Indian Ocean populations ($F_{ST} = 0.740$; IP < 0.001). The construction of a haplotype network provided evidence of gene flow and haplotype sharing between populations. This study indicated the presence of a complex population of *S. lewini* with limited connectivity in Indonesia, and in the Western Indian Ocean (at the regional scale), which will require co-management actions across regions.

Introduction

The scalloped hammerhead shark, *Sphyrna lewini* is considered a coastal species because of its need for nursery areas; its distribution circumglobally in tropical waters; and its abundance, both around continental margins and close to the coastal region and islands [1,2]. This species has the unique modification of a laterally expanded head, which enhances the ability to navigate and follow geomagnetic orientations across the ocean [3–

5]. *S. lewini* has the capacity to move in high rates of dispersal. Female *S. lewini* specimens exhibit site fidelity to single nursery areas and exhibit no evidence of continued inter-oceanic migration. Male *S. lewini* species are spread over a large area across ocean waters, with clear evidence of cross-reproduction and gamete transmission [6].

S. lewini is one of the most exploited and threatened sharks. Some reports mention around one to three million sharks are killed annually worldwide because of fishing and the shark fin trade [7,8]. This species was considered to be underexploited in 1999, however, in 2009 the International Union for Conservation of Nature (IUCN) listed the species on the Red List with Endangered (EN) status [9]. Five years later the Convention on International Trade in Endangered Species (CITES) included hammerhead shark in Appendix II, and in 2019 the vulnerability status was raised to Critical Endangered (CR) [10].

High exploitation of the population of *S. lewini* may have an impact on population structure, reducing the fecundity of the species and the genetic diversity of its populations [11]. Hammerhead sharks are viviparous with a yolk-sac placenta which annual number of young 12-30 per litter. This species has a slow growth rate, late sexual maturity, long gestation period, and a long lifetime in nature [12-15]. The Combination of high pressure from the capture fisheries and their biological characteristics make this species vulnerable to extinction.

The study of population genetics has become an important tool for understanding population connectivity, defining fisheries management, and conservation strategies. Genetic information can be used to define conservation unit and action plan through investigation on genetic and population structure of shark [16-19], tracing the source of populations supplying the international shark product trade particularly for *S. lewini*

which one of the most abundant products in global market in Hongkong [20], and promote implement trade regulation of endangered species, sustainable shark fisheries and effective conservation [16,20,21]. The genetics of the population structure of *S. lewini*, which is important for fisheries stock management, has been widely investigated in different coastal areas and ocean basins on a global and regional scale [17-19,22,23]. Duncan *et al*. [22] reported a global phylogeographic study of *S. lewini* that indicated that the Indo-West Pacific region is the center of diversity for tropical sharks, such as *S. lewini*, with a high and unique genetic diversity; however, no samples from Indonesia were included in that study, or in the study reported by Ovenden *et al.* [23], which included limited samples from Indonesia.

The aim of this study was to investigate the genetic diversity, population structure, and connectivity of *S. lewini* among populations in Indonesia, where the populations of this species are affected by fishing activities, and to observe the connectivity of this species at a regional scale in the Western Indian Ocean. Finally, we examined the implications of these results for species management and conservation.

Material and Methods

Ethics Statement

All specimens were already dead when collected, and no live specimens were obtained. Therefore, no approval from any institutional animal ethics committee was required. The sample collection and transportation were in accordance with the regulation of the Ministry of Marine Affairs and Fisheries of the Republic of Indonesia (Number 5/PERMEN-KP/2018) on the prohibition of cowboy shark and hammerhead shark export from Indonesia. This study was also approved by Ministry of Marine Affairs and Fisheries

of the Republic of Indonesia, under permission numbers 276/BPSPL.03/PRL/X/2018 and 319/PNK/BPSPL.03/PK.230/REKOM/X/2018.

Tissue Sample Collection

A total of 100 tissue samples of *S. lewini* were collected from October 2017 to November 2018, including 41 samples from the Meulaboh and Aceh Jaya fishing ports (Aceh), 30 samples from a local shark landing in Manggar, Balikpapan, and 29 samples from the Tanjung Luar fishing port (Lombok) (Table 1). The samples (~0.5 cm3) were dissected from flesh or fin tissues and preserved in sample bottles containing 96% ethanol.

Table 1. Sampling collection sites at major shark landing sites in Indonesia

Site	Geographic coordinate	Number of samples
**Aceh (ACH)**		
Meulaboh	N 04° 08’ 29” E 96° 07’ 55”	33
Aceh Jaya	N 04° 38’ 34” E 95° 34’ 58”	8
**Balikpapan (BPN)**		
Manggar	S 01° 12’ 53” E 116° 58’ 24”	30
**Lombok (LOM)**		
Tanjung Luar	S 08° 46’ 39” E 116° 31’ 01”	29
**Total**		**100**

DNA Extraction, Amplification, and Sequencing

DNA extraction was performed at the Biodiversity and Biosystematics Laboratory, IPB University, according to the protocol of the gSYNC DNA extraction kit product. A fragment of the mitochondrial cytochrome oxidase subunit 1 (*CO1*) gene was amplified using the forward primer fish-BCL (5'–TCA ACY AAT CAY AAA GAT ATY GGC AC–3′) and the reverse primer fish-BCH (5'–ACT TCY GGG TGR CCR AAR AAT CA–3′) [24,25] in a 24 µL reaction mixture consisting of 3 µL of DNA template, 12.5 µL of

MyTaq HS Red Mix, 9 μL of ddH_2O, 1.25 μL each of forward and reverse primers. The reaction mixture was processed in a polymerase chain reaction (PCR) on a thermocycler using modified cycling conditions [26,27]: pre-denaturation at 94°C for 15 s; followed by 38 cycles of denaturation at 94°C for 30 s, annealing at 50°C for 30 s, and extension at 72°C for 45 s; and a final extension at 72°C for 10 min. The amplicons were visualized by 1.5% agarose gel electrophoresis added with ethidium bromide at 100 V for 20 min. The gel was observed under UV light to identify bands indicating the presence of DNA fragments. Sequencing was performed using sequencing machine with an optimized protocol of Sanger method [28].

All laboratory protocol both on sampling method and DNA identification were described in a short protocol deposited in protocols.io platform_with digital object identifier (DOI) available at dx.doi.org/10.17504/protocols.io.bfwmjpc6.

Data Analysis

Genetic Diversity

The sequences of 179 mitochondrial *CO1* DNA sequences with an average length of 594 bp were edited and aligned using the ClustalW algorithm [29] implemented in MEGA 6.06 [30]. Genetic diversity parameters, including the number of haplotypes, haplotype diversity (h), and nucleotide diversity (π), were calculated using the DNASp v6 [31] and Arlequin v.3.5 program [32]. We also reanalyzed additional *CO1* sequencing data of *S. lewini* from West Papua retrieved from GenBank (Table 2) (n = 14), which were obtained by Sembiring *et al.* [33], and sequences from previous studies performed in India (n = 6) [34], the United Arab Emirates (n = 30) [35], and Madagascar (n = 29) [36], to assess the genetic diversity in Indonesian and Western Indian Ocean populations.

Table 2. Localities, total number (n), and accession number of *CO1* gene sequences of *S. lewini* from Aceh, Balikpapan, Lombok, and Western Papua (Indonesia) and India, the United Arab Emirates, and Madagascar (Western Indian Ocean)

Locality	n	Accession number	Author
**Indonesia**			
Aceh	41	MT324149-156, MT324187-219	This study
Balikpapan	30	MT324157-186	This study
Lombok	29	MT324220-248	This study
West Papua	14	KF590254-55, KF590271-76, KF793729, KF793738-42	[33]
**Western Indian Ocean**			
India	6	KF899746-51	[34]
United Arab Emirates	30	KP177238-41, KP177241, KP177254, KP177262, KP177272, KP177285-99, KP177300-07	[35]
Madagascar	29	HQ171735-47, HQ171761-76	[36]

Population Structure

An analysis of molecular variance (AMOVA) and fixation index (F_{ST}) analysis [37] were performed for three major group: 1) within and among the four populations from Indonesia, 2) within and among the three populations from the Western Indian Ocean, and 3) comparison between populations from Indonesia and Western Indian Ocean using the Arlequin v.3.5 program (set up, 1000 permutations; significance level threshold, $\alpha = 0.05$), to estimate the overall extent of the genetic variation and differentiation level in Indonesia and the Western Indian Ocean. Population differentiation and its significance between sampling sites were also calculated with pairwise estimates [38-40].

Genetic Connectivity

A haplotype network was constructed with a median-joining method in the Network v5.1.1.0 program [41] for all haplotypes detected in Indonesia and the Western Indian Ocean, to obtain haplotype connectivity in a broader spatial connection in the regional area of the Indian Ocean. The distribution of haplotypes for each location was also provided in a proper map, to show the clear distributions of haplotypes and genetic connectivity among the populations under study.

Results

Genetic Diversity

All sequences of *S. lewini* obtained in this study were deposited in the BOLD System with the Barcode Index Number (BIN) registry of BOLD:AAA2403 and database of GenBank with accession numbers MT324149-248 (Table 2). A total of 179 sequences of 594 bp of the mitochondrial *CO1* gene of *S. lewini* obtained from three sampling sites (Aceh, Balikpapan, and Lombok) and additional sequences of samples from West Papua and the West Indian Ocean region were used to generate a total of 11 haplotype variations with 40 polymorphic loci (Table 3).

Table 3. Forty polymorphic loci of 11 haplotypes from 179 sequences of the mitochondrial *CO1* gene of *S. lewini* samples from four localities in Indonesia and three localities in the Western Indian Ocean region

Haplotypes	Locus Position																																							
	**1**	**15**	**24**	**27**	**33**	**45**	**48**	**63**	**69**	**105**	**120**	**138**	**141**	**156**	**165**	**174**	**186**	**198**	**229**	**279**	**289**	**303**	**306**	**325**	**339**	**342**	**346**	**366**	**396**	**420**	**453**	**456**	**459**	**483**	**495**	**498**	**522**	**531**	**534**	**543**
H1*	A	T	A	C	T	T	G	T	T	A	T	C	G	T	T	C	C	T	T	C	G	T	T	C	C	A	C	T	T	C	C	T	T	C	T	C	C	T	C	T
H2*	.	.	.	A	.	.	.	.	.	G	.	A	T	.	C	T	T	C	C	T	.	C	C	T	T	C	.	C	C	T	.	.	.	A	.	T	.	C	.	C
H3	.	.	.	A	.	.	.	.	C	G	.	A	T	.	C	T	T	C	C	T	.	C	.	T	T	C	.	C	C	T	.	.	.	A	.	T	.	C	.	.
H4*	.	.	.	A	.	.	.	.	.	G	.	A	T	.	C	T	T	C	C	T	.	C	C	T	T	C	.	C	C	T	.	.	.	A	.	T	.	C	.	.
H5	.	.	.	.	.	.	.	.	.	.	.	.	.	.	.	.	.	.	.	.	.	.	.	.	.	.	.	.	.	.	.	.	G	.	G	.	.	.	.	.
H6	.	.	.	.	.	C	.	.	.	.	.	.	.	.	.	.	.	.	.	.	.	.	.	.	.	.	.	.	.	.	.	.	.	.	.	.	.	.	.	.
H7	.	.	C	.	.	.	.	.	.	.	.	.	.	G	.	.	.	.	.	.	.	.	.	.	.	.	.	.	.	.	.	.	.	.	.	.	.	.	.	.
H8	.	C	.	.	C	.	.	.	.	.	.	.	.	.	.	.	.	.	.	.	.	.	.	.	.	.	.	.	.	.	.	.	.	.	.	.	.	.	.	.
H9	.	.	.	.	.	.	.	.	.	.	G	.	.	.	.	.	.	G	.		T	.	.	.	.	.	.	.	.	.	.	.	.	.	.	.	.	.	G	.
H10	G	.	.	.	.	.	.	G	.	.	.	.	.	G	.	.	.	.	.	.	.	.	.	.	.	.	.	.	.	.	.	.	.	.	.	.	.	.	.	.
H11	.	.	.	.	.	.	.	.	.	.	.	.	.	.	.	.	.	.	.	.	.	.	.	.	.	.	T	.	.	.	T	.	.	.	C	.	T	.	.	.

Notes:

Nucleobase at each position is given for H1 and only nucleobases that were different from H1 are written for all other haplotypes. Nucleobases identical to H1 are indicated with dots (**.**)

*Three original haplotypes of the *S. lewini* populations from the three areas of study (Aceh, Balikpapan, and Lombok) in Indonesia. The remaining haplotypes were reanalyzed from previous studies.

The comparison of the genetic diversity of *S. lewini* based on haplotype and nucleotide diversity revealed the presence of variation (Table 4). The haplotype diversity (h) among the localities in Indonesia ranged from 0.143 to 0.668 and the nucleotide diversity (π) ranged from 0.000 to 0.020. The highest genetic diversity in Indonesia was observed for the population from Aceh (h = 0.668; π = 0.020), followed by the Balikpapan population, which exhibited a lower haplotype and nucleotide diversity (h = 0.646; π = 0.002), and the lowest genetic diversity was detected in West Papua (h = 0.143, π = 0.000). The *S. lewini* population from Lombok also exhibited a fairly low genetic diversity (h = 0.362; π = 0.001). However, the overall genetic diversity in Indonesia was relatively high (h = 0.717; π = 0.013). Conversely, the average genetic diversity of the Western Indian Ocean region was low and ranged from 0.000 to 0.467. The overall diversity in the Western Indian Ocean region was also low (h = 0.232; π = 0.001).

Table 4. Genetic diversity of *S. lewini* based on sample size (n), haplotype number (Hn), haplotype diversity (h), and nucleotide diversity (π) in samples from each site in Indonesia and the Western Indian Ocean region

Population	n	Genetic Diversity		
		Hn	h	π
**Indonesia**				
Aceh (ACH)	41	4	0.668	0.020
Balikpapan (BPN)	30	3	0.646	0.002
Lombok (LOM)	29	3	0.362	0.001
West Papua (WEP)	14	2	0.143	0.000
Overall Indonesia	114	4	0.717	0.013
**Western Indian Ocean**				
India (IND)	6	1	0.000	0.000
United Arab Emirates (UAE)	30	8	0.467	0.002
Madagascar (MDG)	29	1	0.000	0.000
Overall Western Indian Ocean	65	8	0.232	0.001

Population Structure

The analysis of the fixation index (F_{ST}) and the corresponding *P*-values between and within the four *S. lewini* populations (ACH, BPN, LOM, and WEP) from Indonesia and the three populations (IND, UAE, and MDG) from the Western Indian Ocean region are shown in Table 5. The overall F_{ST} value in Indonesia was significantly higher than that observed in other regions ($F_{ST} = 0.442$; $P < 0.001$), which indicated the presence of multiple subdivisions in Indonesia.

No significant differentiation was observed in the population from the Western Indian Ocean region (F_{ST} = –0.013; P = 0.601), while a comparison of the population structure between Indonesia and the Western Indian Ocean region yielded significant differentiation (F_{ST} = 0.740; $P < 0.001$). The pairwise F_{ST} values between the populations from the four locations in Indonesia and a comparison with those from Western Indian Ocean population are shown in Table 6. The overall pairwise analysis via the distance method indicated the presence of significant differentiation among the four populations from Indonesia, with the exception of BPN and LOM (F_{ST} = 0.044; P = 0.090), which showed fairly low F_{ST} values and no significant p values. Furthermore, among the populations from Indonesia, the ACH population tended to be closer to the Western Indian Ocean population, as it exhibited a lower F_{ST} value (rather than F_{ST} range) compared with other localities from Indonesia to the Western Indian Ocean.

Table 5. Analysis of molecular variance (AMOVA) for percentage of variation (%), F_{ST} value, and significance level (*P*-value) in *S. lewini* samples from Indonesian populations, Western Indian Ocean populations, and between Indonesian and Western Indian Ocean populations

**Source of variation**	**df**	**Percentage of variation (%)**	**F_{ST} value**	***P* value**
**Indonesia**				
Among Populations	3	44.15	0.442	0.000
Within Populations	110	55.85		
Total	113			
**Western Indian Ocean**				
Among Populations	2	−1.31	−0.013	0.601
Within Populations	62	101.31		
Total	64			
**Indonesia vs. Western Indian Ocean**				
Among Populations	1	74.04	0.740	0.000
Within Populations	177	25.96		
**Total**	**178**			

Table 6. Pairwise F_{ST} values (below the diagonal) and *P*-values (above the diagonal) between the *S. lewini* populations from Aceh (ACH), Balikpapan (BPN), Lombok (LOM), West Papua (WEP), and Western Indian Ocean (WIO)

**Sample Sites**	**ACH**	**BPN**	**LOM**	**WEP**	**WIO**
**ACH**	-	0.000	0.000	0.000	0.000
**BPN**	0.427	-	0.090	0.001	0.000
**LOM**	0.438	0.044	-	0.000	0.000
**WEP**	0.398	0.495	0.736	-	0.000
**WIO**	0.509	0.965	0.973	0.975	-

Genetic Connectivity

A haplotype network analysis identified two main groups of haplotypes (Fig. 1), referred to as Clade A and Clade B henceforth. Clade A consisted of haplotype H1, which was observed in several regions, i.e., Aceh, India, United Arab Emirates, and Madagascar, whereas H5, H6, H7, H8, H9, H10, and H11 were present only in the samples from the United Arab Emirates. Clade B consisted of three haplotypes (H2, H3, and H4), which were spread evenly in Indonesia, although each region exhibited predominant haplotypes. Haplotypes H1, H2, H4, and H3 were predominant in Aceh, Balikpapan, Lombok, and West Papua, respectively (Fig. 2).

Fig 1. Haplotype network of the *S. lewini* (n = 179) population from Indonesia and the Western Indian Ocean region, which was constructed using the Median Joining method

Fig 2. Distribution of the 11 haplotypes of the *S. lewini* population from Indonesia and Western Indian Ocean at the regional scale

Discussion

Genetic Diversity

The overall genetic diversity of *S. lewini* at the haplotype and nucleotide levels was relatively high for populations in Indonesia. These findings are consistent with the results reported by Ovenden *et al.* [23], pertaining to the mitochondria control region of *S. lewini* from three localities in Indonesia, including Lombok, and is a highly genetic diversity of *S. lewini* observed at the global scale [22]. The scalloped hammerhead sharks are a highly

migratory species with a wide distribution in tropical and warm-temperate waters and possibly move across oceanic waters to a distance of up to 1671 km [12]. In principle, because of migration capability and broad ecological niches, this species tends to have a higher genetic diversity than do species from narrow ecological niches [42]. High genetic diversity values are commonly related to large population sizes [43] and are promoted by several factors, such as local population sizes, fast generation times [44], high nucleotide substitution rates [45], and high gene flow between geographically distant populations.

The finding of a relatively high genetic diversity for *S. lewini* in Indonesia appears to be inconsistent with the general assumption that overexploitation of this species as both a target of fishing and bycatch has led to the decline of its populations worldwide [46]. However, the results obtained here for the population from Lombok may be relevant in this context; i.e., the lower genetic diversity detected at that location was probably driven by continuous fishing pressure. *S. lewini* were the top three targeted sharks at the Tanjung Luar fishing port in Lombok and have faced high fishing pressure over more than 40 years, which has led to its overfishing, with recent exploitation rates (E) reaching 0.59 [47].

Furthermore, according to the global fisheries information system on hammerhead shark capture production 1950-2017 on global scale and Indonesia by FAO [48], the hammerhead sharks (Sphyrnidae), including *S. lewini*, are important sharks that were highly exploited in the last two decades, with an estimated rapid increase in global capture, from 220 ton per year in 1985 up to approximately 10,362 tons per year in 2016. In the same period, the capture level in Indonesia also increased significantly, reaching approximately 1,492 tons in 2016. Moreover, Indonesia exhibited one of the highest

volumes of sharks and rays caught on the global catches reported in the period of 2000–2011 [49].

Clarke *et al*. [50] reported similar findings regarding the mitochondrial DNA of the silky shark, *Carcharhinus falciformis*, which exhibited a high genetic diversity under circumstances of overexploitation. Elasmobranchs exhibit adaptability to environmental and anthropogenic stresses, which causes genetic bottlenecks because of their particular life histories [51]. Moreover, the population decline caused by recent fishery activities might be insufficient to reduce genetic diversity, particularly for species with a long-life span (13–20 years), including *S. lewini* [52,53].

However, the continuous decline in these populations, as was expected to occur for the *S. lewini* population from Lombok, was positively correlated with the loss of genetic diversity and created a population bottleneck [53,54], as reported by Pinsky and Palumbi in a meta-analysis of several marine fish [55].

Population Structure

Our findings indicated the presence of homogeneity among the *S. lewini* populations from Balikpapan and Lombok. The pairwise F_{ST} analysis detected no significant genetic differentiation in these two populations with lowest value of F_{ST}. These finding complements those of previous studies conducted in Indo-Australian waters. Ovenden *et al.* [23] reported similar evidence for the mitochondrial control region regarding population structure, with no differentiation between two populations of *S. lewini* (Lombok and northern Australia). This pattern of single-stock population seems to suggest that these localities are a migration zone of *S. lewini* and that a reproductive movement might occur in the coastal areas of these locations, although there are strong

Indonesian throughflow currents between Kalimantan and Sulawesi island. Adult *S. lewini* specimens are highly migratory, with a large body size that supports the high dispersal capacity of this species; hence, they exhibit the possibility of overcoming that geographical barrier. In contrast, we obtained different results from the comparison among these two populations from central Indonesia and one population from Aceh (western Indonesia) and one population from West Papua (eastern Indonesia), which exhibited strong genetic differentiation. These regions were spatially separated by a long distance, with the possibility of the existence of more complex barriers, both inter-island barriers and anthropogenic factors pertaining to a high commercial and artisanal fishing pressure, such as those found along the southern and northern coasts of Java.

Genetic Connectivity

Regarding significant value of F_{ST} among population ($F_{ST} = 0.740$; $P < 0.001$) and haplotype network and distribution shown in Fig 1 and 2, we observed two restricted haplogroup which separated *S. lewini* population in Indonesian with Western Indian Ocean population. Those two haplogroup were separated with 19 different nucleotide bases as result of monomorphic and polymorphic mutation. Limited gene flow that occurs only among populations in Indonesia forms a different gene pool with Western Indian Ocean population. It can be expected due to a complex geographic barrier in Indonesia's marine ecosystem, and confirmed by global distribution pattern of S. lewini, with significant separation population across ocean basin and discontinuous coastlines habitat [6].

Overall, in Indonesia, *S. lewini* populations from Balikpapan, Lombok, and West Papua seemed to be isolated from the population from the Western Indian Ocean, and shared a

haplotype network exclusively only in eastern Indonesian waters. However, we obtained an interesting result regarding haplotype sharing between the populations from Aceh and the Indian Ocean population. H1 was a unique haplotype found in Aceh, Indonesia, and was correlated with the most common haplotype observed in samples from the three locations of the Western Indian Ocean region (India, United Arab Emirates, and Madagascar), which indicates the presence of connectivity between the populations from Aceh and those from the Western Indian Ocean region. Schott and McCreary [56] reported that the Indian Ocean seasonal current carried water from north Sumatra through the Arabian Sea to Madagascar, which represents a possible connectivity channel between the two regions, thus allowing *S. lewini* to migrate and leading to breeding and gene flow. The similarity between the predominant haplotype (H1) of *S. lewini* from Aceh with that of the populations from India, United Arab Emirates, and Madagascar evidenced a genetic sharing process among populations in the Indian Ocean region. However, the *S. lewini* populations from Balikpapan, Lombok, and West Papua seemed to be isolated from the population from the Western Indian Ocean, and shared a haplotype network exclusively in eastern Indonesian waters.

Conservation Implications

The overall high genetic diversity of the *S. lewini* populations in Indonesia suggests that this species has not yet experienced a genetic loss because of exploitation pressure. However, the lower genetic diversity of *S. lewini* from Lombok and West Papua indicated a higher risk of loss of genetic diversity, which probably was the result of high fisheries pressure. Our genetic assessment of *S. lewini* samples from four localities revealed that a single stock exists between Lombok and Balikpapan, while a separate stock was observed

for Aceh and West Papua, indicating that the management of this species should occur on a stock-based approach at least on three mitochondrial-stock conservation management units. The complex population of *S. lewini* with limited connectivity observed in Indonesia and the Western Indian Ocean region indicated the importance of promoting collaborative management strategies among Indonesian management agencies and in conjunction with Western Indian Ocean agencies at the regional scale.

Conclusion

This study provided important findings regarding the population structure of *S. lewini* in Indonesia, with a high genetic diversity and three significant subdivisions that indicated the capability of the population to adapt to rapid environmental changes and pressure, including fishing activities. However, the lower genetic diversity detected in Lombok and West Papua should be considered. The genetic sharing detected among the populations from Indonesia and also those from the Western Indian Ocean proved the connectivity among these populations. A collaborative action across regions is needed to promote sustainable management and conservation purposes, both in Indonesia and at the regional scale in the Western Indian Ocean area.

Acknowledgement

We would like to thank to the institutions and individuals that have made our study possible: colleagues from Marine Biodiversity and Biosystematics Lab (BIODIVSI), Faculty of Marine Science and Technology, IPB University for their help during research. We also appreciated to BPSPL Satker Balikpapan, Listian Nova and Mr. Hery, Head of TPI Manggar, Balikpapan for their help during collecting samples, and to reviewers and

proof readers who have provided highly constructive suggestions for better writing of the paper.

References

1. Klimley AP, Butler SB. Immigration and emigration of a pelagic fish assemblage to seamounts in the Gulf of California related to water mass movements using satellite imagery. Mar Ecol Prog Ser. 1988;49: 11-20.
2. Compagno LJV. FAO Species Catalogue: Sharks of the world. Rome: Food and Agriculture Organization;1984.
3. Montgomery JC, Walker MM. Orientation and navigation in elasmobranchs: which way forward? Environ Biol Fishes. 2001;60: 109-116.
4. Kajiura SM, Holland KN. Electroreception in juvenile scalloped hammerhead and sandbar sharks. J Exp Biol. 2002;205: 3609-3621.
5. Meyer CL, Holland KN, Papastamatiou YP. Sharks can detect changes in the geomagnetic field. J R Soc Interface. 2005;2: 129-130.
6. Daly-Engel TS, Seraphin KD, Holland KN, Coffey JP, Nance HA, Toonen RJ, et al. Global phylogeography with mixed-marker analysis reveals male-mediated dispersal in the endangered scalloped hammerhead shark (*Sphyrna lewini*). PLoS ONE. 2012;7(1): e29986. doi: 10.1371/journal.pone.0029986.
7. Clarke SC, McAllister MK, Milner-Gulland EJ, Kirkwoo GP, Michielsens CGJ, Agnew DJ et al. Global estimates of shark catches using trade records from commercial markets. Ecol Lett. 2006;9(10): 1115-1126.

8. Chapman DD, Simpfendorfer CA, Wiley TR, Poulakis GR, Curtis C, Tringali M et al. Genetic diversity despite population collapse in a critically endangered marine fish: the smalltooth sawfish (*Pristis pectinata*). J Hered. 2011;102: 643-652.
9. Baum J, Clarke S, Domingo A, Ducrocq M, Lamónaca AF, Gaibor N et al. Sphyrna lewini. The IUCN Red List of Threatened Species 2009. 2009; e.T39385A10190088.
10. Rigby CL, Dulvy NK, Barreto R, Carlson J, Fernando D, Fordham S et al. Sphyrna lewini. The IUCN Red List of Threatened Species 2019. 2019; e.T39385A2918526.
11. Ward RD. Genetics in fisheries management. Hidrobiologia. 2000;420: 191–201.
12. Kohler NE, Turner PA. Shark tagging: a review of conventional methods and studies. Environ Biol Fishes. 2001;60: 191-223.
13. White W, Barton C, Potter I. Catch composition and reproductive biology of *Sphyrna lewini* (Griffith & Smith) (Charcharhinifirnes, Sphyrnidae) in Indonesian waters. J Fish Biol. 2008;72: 1675-1689.
14. Ovenden J, Morgan JT, Street R, Tobin A, Simfendorfer CA, Macbeth W, Welch D. Negligible evidence for regional genetic population structure for two shark species *Rhizoprionodon acutus* (Rüppell, 1837) and *Sphyrna lewini* (Griffith & Smith, 1834) with contrasting biology. Mar Biol. 2011;158: 1497-1509.
15. Bessudo S, Soler GA, Kimley PA, Ketchum J, Arauz R, Hearn A, et al. Vertical and horizontal movements of scalloped hamemerhead shark (*Sphyrna lewini*) around Malpelo and Cocos islands (Tropical Eastern Pacific) using satellite telemetry. Boletín de Investigaciones Marinas y Costeras. 2011;40: 91-106.
16. Cardeñosa D, Hyde J, Caballero S. Genetic diversity and population structure of the pelagic thresher shark (*Alopias pelagicus*) in the Pacific Ocean: evidence for two

evolutionarily significant units. PLoS ONE. 2014;9(10): e110193. doi: 10.1371/journal.pone.0110193.

17. Castillo-olguin E, Aribe-Alcocer M, Diaz-Jaimes P. Assessment of the population genetic structure of *Sphyrna lewini* to identify conservation units in the Mexican Pacific. Ciencias Marinas. 2012;38(4): 635-652. doi: 10.7773/cm.v38i4.2110.
18. Quintanilla S, Gómez A, Mariño-Ramírez C, Sorzano C, Bessudo S, Soler G, et al. Conservation genetics of the scalloped hammerhead shark in the Pacific Coast of Colombia. J Hered. 2015;106: 448-458. doi:10.1093/jhered/esv050.
19. Sukumaran S, Sebastian W, Mukundan LP, Menon M, Akhilesh K V., Zacharia PU, et al. Molecular analyses reveal a lack of genetic structuring in the scalloped hammerhead shark, Sphyrna lewini (Griffith & Smith, 1834) along the Indian coast. Mar Biodivers. 2020;50. doi:10.1007/s12526-020-01040-4.
20. Fields AT, Fischer GA, Shea SKH, Zhang H, Feldheim KA, Chapman DD. DNA Zip- coding: identifying the source populations supplying the international trade of a critically endangered coastal shark. Animal Conservation. 2020; doi:10.1111/acv.12585.
21. Jabado RW, Al Ghais SM, Hamza W, Henderson AC, Spaet JLY, Shivji MS, et al. The trade in sharks and their products in the United Arab Emirates. Biol Conserv. 2015;181: 190–198. doi:10.1016/j.biocon.2014.10.032
22. Duncan KM, Martin AP, Bowen BW, De Coute HG. Global phylogeography of the scalloped hammerhead shark (Sphyrna lewini). Mol Ecol. 2006;15:2239-51.
23. Ovenden JR, Kashiwagi T, Broderick D, Giles J, Salini J. The extent of population genetic subdivision differs among four co-distributed shark species in the Indo-Australian archipelago. BMC Evol Biol. 2009;9: 40. doi:10.1186/1471-2148-9-40.

24. Baldwin CC, Mounts JH, Smith DG, Weigt LA. Genetic identification and color descriptions of early life-history stages of Belizean *Phaeoptyx* and *Astrapogon* (Teleostei: Apogonidae) with comments on identification of adult *Phaeoptyx*. Zootaxa. 2009;2008: 1-22.
25. Madduppa H, Ayuningtyas RU, Subhan B, Arafat D. Exploited but unevaluated: DNA barcoding reveals skates and stingrays (Chordata, Chondrichthyes) species landed in the Indonesian fish market. IJMS. 2016;21: 77-84.
26. Prehadi, Sembiring A, Kurniasih EM, Rahmad, Arafat D, Subhan B et al. DNA barcoding and phylogenetic reconstruction of shark species landed in Muncar fisheries landing site in comparison with Southern Java fishing port. Biodiversitas. 2015;16: 55-61.
27. Hadi S, Anggraini NP, Muttaqin E, Simeon BM, Subhan B, Madduppa H. Genetic diversity of the endangered species Sphyrna lewini (Griffith and Smith 1834) in Lombok based on mitochondrial DNA. IOP Conf. Ser.: Earth Environ. Sci. 2019;236: 012024.
28. Sanger F, Nicklen S, Coulson AR. DNA sequencing with chain-terminating inhibitors. Proc Natl Acad Sci. 1977;74(12): 5463-5467. doi:10.1073/pnas.74.12.5463.
29. Thompson JD, Higgins DG, Gibson TJ. CLUSTAL W: improving the sensitivity of progressive multiple sequence alignment through sequence weighting, position-specific gap penalties and weight matrix choice. Nucleic Acids Res. 1994;22(22): 4673-4680.

30. Tamura K, Stecher G, Peterson D, Filipski A, Kumar S. Molecular Evolutionary Genetics Analysis Version 6.0. Mol Biol Evol. 2013;30(12): 2725-2729. doi: 10.1093/molbev/mst197.
31. Rozas J, Ferrer-Mata A, Sánchez-DelBarriol JC, Guirao-Rico S, Librado P, Ramos-Onsins SE. DnaSP v6: DNA Sequence polymorphism analysis of large datasets. Mol Biol Evol. 2017;34: 3299-3302.
32. Excoffier L, H.E. L. Lischer. Arlequin suite ver 3.5: A new series of programs to perform population genetics analyses under Linux and Windows. Mol Ecol Resour. 2010;10: 564-567.
33. Sembiring A, Pertiwi NPD, Mahardini A, Wulandari R, Kurniasih EM, Kuncoro AW, et al. DNA barcoding reveals targeted fisheries for endangered sharks in Indonesia. Fisheries Research. 2015;164: 130-134. doi: 10.1016/j.fishres.2014.11.003.
34. Bineesh KK, Gopalakrishnan A, Akhilesh KV, Sajeela KA, Abdussamad EM, Pillai NGK, et al. DNA barcoding reveals species composition of sharks and rays in the Indian commercial fishery. Mitochondrial DNA. 2016. doi: 10.3109/19401736.2015.1137900.
35. Jabado RW, Ghais SMA, Hamza W, Henderson AC, Spaet JLY, Shivji MS, et al. The trade in sharks and their products in the United Arab Emirates. Biol Conserv. 2015;181: 190-198. doi: 10.1016/j.biocon.2014.10.032.
36. Doukakis P, Hanner R, Shivji M, Bartholomew C, Chapman D, Wong E, et al. Applying Genetic Techniques to Study Remote Shark Fisheries in Northeastern Madagascar. Mitochondrial DNA. 2011;22(S1): 15-20.

37. Wright S. The genetical structure of populations. Annals of Eugenics. 1951;15: 323-354.
38. Weir BS, Cockerham CC. Estimating F-Statistics for the Analysis of Population Structure. Evol. 1984;38(6): 1358. doi:10.2307/2408641.
39. Excoffier L, Smouse PE, Quattro JM. Analysis of molecular variance inferred from metric distances among DNA haplotypes: application to human mitochondrial DNA restriction data. *Genetics*. 1992;131(2): 479- 491.
40. Weir BS. Genetic Data Analysis II: Methods for Discrete Population Genetic Data. Sunderland, Mass: Sinauer Associates; 1996.
41. Bandelt H-J, Forster P, Röhl A. Median-joining networks for inferring intraspecific phylogenies. Mol Biol Evol. 1999;16: 37-48.
42. Habel JC, Schmitt T. Vanishing of the common species: Empty habitats and the role of genetic diversity. Biol. Conserv. 1996;218: 211-216. doi: 10.1016/j.biocon.2017.12.018.
43. Frankham R. Relationship of Genetic Variation to Population Size in Wildlife. Conserv. Biol. 1996;10(6): 1500-1508. doi:10.1046/j.1523-1739.1996.10061500.x.
44. Hague MTJ, Routman EJ. Does population size affect genetic diversity? A test with sympatric lizard species. Heredity. 2016;116: 92-98. doi: 10.1038/hdy.2015.76.
45. Martin AP, Naylor GJP, Palumbi SR. Rates of mitochondrial DNA evolution in sharks are slow compared with mammals. Nature. 1992;357: 153-157. doi: 10.1038/357153a0.
46. Compagno LJV, Dando M, Fowler SL. Sharks of the World. Princeton: Princeton University Press; 2005.

47. Simeon BM, Agustina S, Muttaqin E, Yulianto I, Ichsan M, Muhsin M. Laporan teknis: Pemanfaatan Hasil Tangkap Hiu dan Pari di Tanjung Luar, Provinsi Nusa Tenggara Barat. Bogor: Wildlife Conservation Society-Indonesia Program; 2017.
48. Food and Agriculture Organization (FAO). Fisheries and Aquaculture Information and Statistics Service: Global Capture Production 1950-2017; Database: Indonesian hammerhead shark capture 2017 [cited 2020 Mar 5]. Available from: http://www.fao.org/fishery/statistics/global-capture-production/query/es.
49. Dent F, Clarke. State of the Global Market for Shark Products. Rome: Food and Agriculture Organization; 2015.
50. Clarke CR, Karl SA, Horn RL, Bernard AM, Lea JS, Hazin FH et al. Global mitochondrial DNA phylogeography and population structure of the silky shark, *Carcharhinus falciformis.* Mar Biol. 2015;162(5): 945-955. doi: 10.1007/s00227-015-2636-6.
51. Chapman DD, Simpfendorfer CA, Wiley TR, Poulakis GR, Curtis C, Tringali M et al. Genetic diversity despite population collapse in a critically endangered marine fish: the smalltooth sawfish (*Pristis pectinata*). J Hered. 2011;102: 643-652.
52. Sánchez-de Ita JA, Quiñónez-Velázquez C, Galván-Magaña F, Bocanegra-Castillo N, Félix-Uraga R. Age and growth of the silky shark *Carcharhinus falciformis* from the west coast of Baja California Sur, Mexico. J App Ichthyol. 2011;27: 20-24.
53. Nei M, Maruyama T, Chakraborty R. The bottleneck effect and genetic variability in populations. Evolution. 1975;29(1): 1. doi:10.2307/2407137.
54. Beebee T, Rowe G. An Introduction to Molecular Ecology. 2nd ed. New York: Oxford University Press Inc; 2008.

55. Pinsky ML, Palumbi SR. Meta-analysis reveals lower genetic diversity in overfished populations. Mol Ecol. 2013;23(1): 29-39. doi:10.1111/mec.12509.
56. Schott FA, McCreary JP. The monsoon circulation of the Indian Ocean. Prog Oceanogr. 2001;51: 1-123. doi: 10.1016/S0079-6611(01)00083-0.

Figure 1

Click here to access/download;Figure;FIG 1 (1).tif

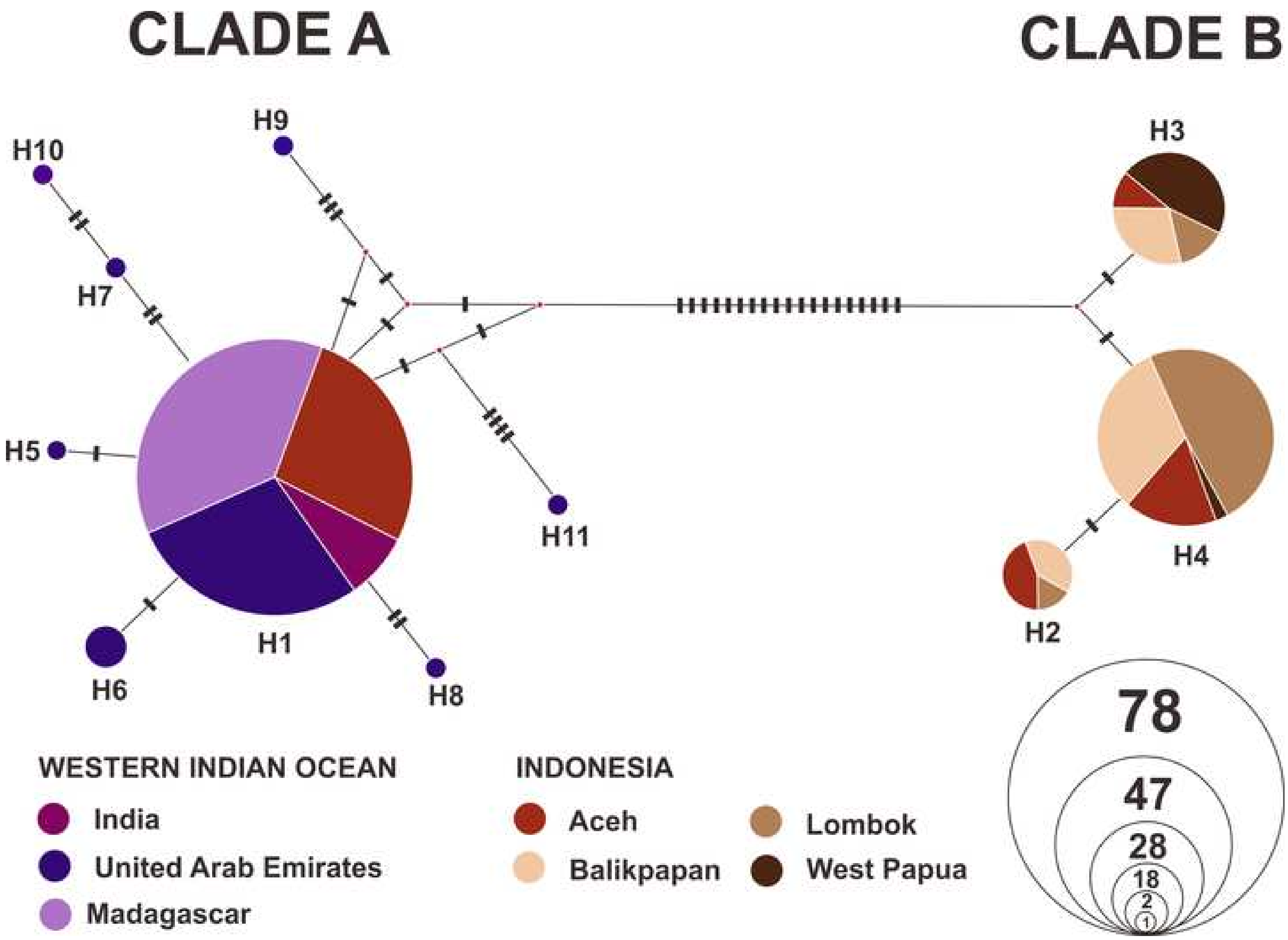

Figure 2

Click here to access/download;Figure;FIG 2 (1).tif

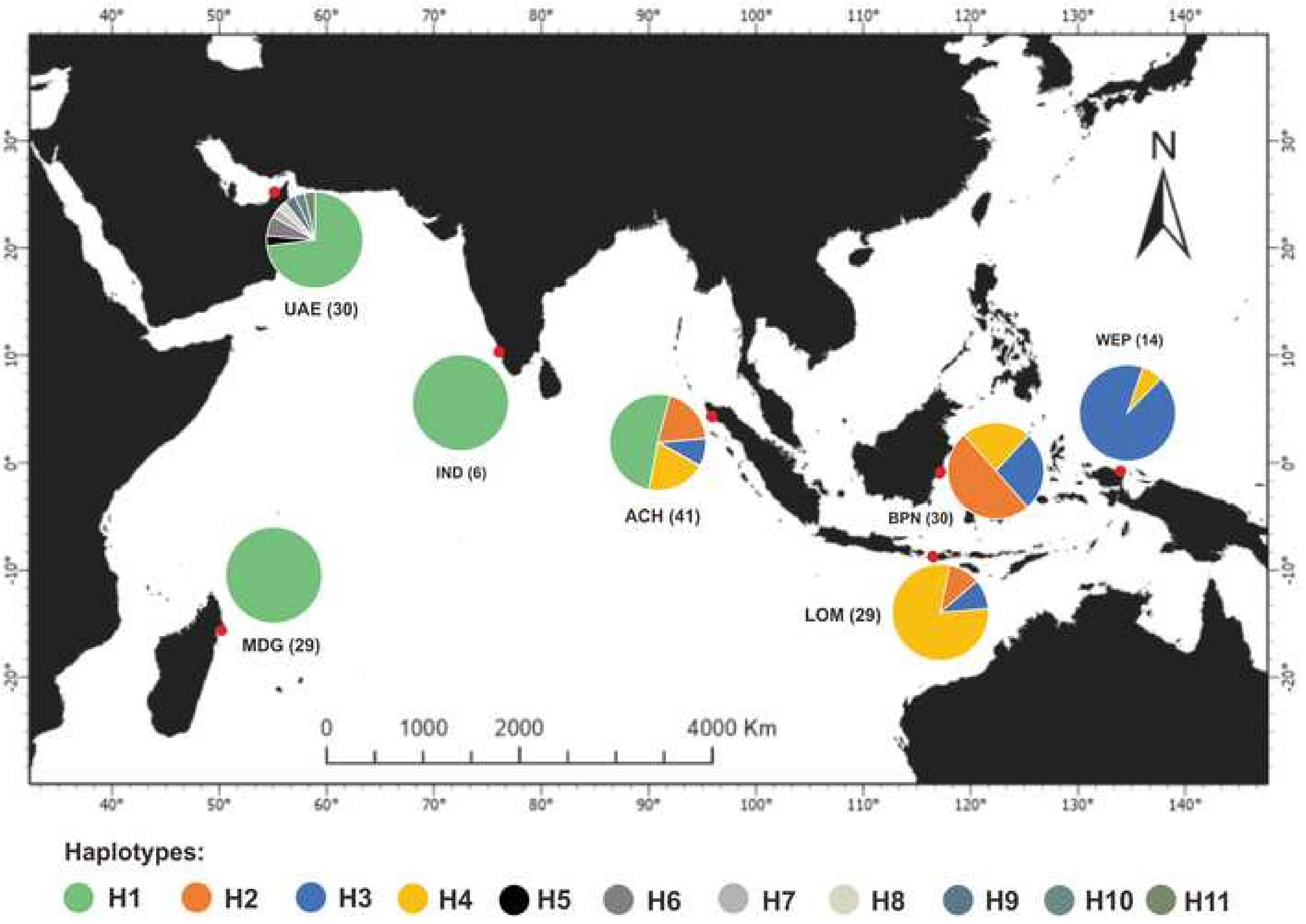

Genetic connectivity of the scalloped hammerhead shark *Sphyrna lewini* (Griffith & Smith 1834) population across Indonesia and the Western Indian Ocean

Sutanto Hadi[1], Noviar Andayani[2,3], Effin Muttaqin[3], Benaya M Simeon[3], Muhammad Ichsan[3], Beginer Subhan[1] and Hawis Madduppa[1]*

[1] Department of Marine Science and Technology, Faculty of Fisheries and Marine Sciences, IPB University, Indonesia

[2] Department of Biology, Faculty of Mathematic and Natural Science, University of Indonesia, Depok, Indonesia

[3] Wildlife Conservation Society Indonesia, Bogor, Indonesia

*Corresponding author

Email: hawis@apps.ipb.ac.id

Abstract

Sphyrna lewini is an endangered species and its populations have been declining worldwide, including in Indonesia, because of overexploitation. However, there is a lack of recent information regarding recent the population structure in this species, to promote its proper management and conservation status in Indonesia. This study aimed to investigate the genetic diversity, population structure, and connectivity of the *S. lewini* population in Indonesia, i.e., in three major shark landing sites: Aceh (n = 41), Balikpapan (n = 30), and Lombok (n = 29); moreover, additional sequences were retrieved from West Papua (n = 14) and Western Indian Ocean (n = 65) populations. Analyses of the

mitochondrial *CO1* gene successfully identified a total of 179 sequences of *S. lewini*, with an average size of 594 bp, and 40 polymorphic loci in four haplotypes for the Indonesian population and in eight haplotypes for the Western Indian Ocean population. The overall values of genetic diversity in Indonesia were high (h = 0.717; π = 0.013), with the highest values recorded in Aceh (h = 0.668; π = 0.002) and the lowest detected in Papua (h = 0.143; π = 0.000), whereas the overall value was fairly low in the Western Indian Ocean (h = 0.232; π = 0.001). AMOVA and F_{ST} revealed three significant population subdivisions in Indonesia (F_{ST} = 0.442; P < 0.001), with separated populations for Aceh and West Papua, and a mixed population between Balikpapan and Lombok (F_{ST} = 0.044; P = 0.089); in contrast, a relatively insignificant differentiation was observed within the population of the Western Indian Ocean (F_{ST} = –0.013; P = 0.601) and a significant different was detected between the Indonesian and the Western Indian Ocean populations (F_{ST} = 0.740; IP < 0.001). The construction of ~~A~~ a haplotype network provided evidence of gene flow and haplotype sharing between populations. This study indicated the presence of a complex population of *S. lewini* with limited connectivity in Indonesia, and in the Western Indian Ocean (at the regional scale), which will require co-management actions across regions.

Introduction

The scalloped hammerhead shark, *Sphyrna lewini* is considered a coastal species because of its need for nursery areas; its distribution circumglobally in tropical waters; and its abundance, both around continental margins and close to the coastal region and islands [1,–2]. This species has the unique modification of a laterally expanded head, which enhances the ability to navigate and follow geomagnetic orientations across the ocean [3–

Formatted: Font: 12 pt

Formatted: Font: 12 pt

Commented [SH1]: Academic Editor:
of a haplotype (typo A)

Sutanto Hadi:
Thank you for your correction. We have fixed it.

Formatted: Font: 12 pt

5]. *S. lewini* has the capacity to move in high rates of dispersal. Female *S. lewini* specimens exhibit site fidelity to single nursery areas and exhibit no evidence of continued inter-oceanic migration. Male *S. lewini* species are spread over a large area across ocean waters, with clear evidence of cross-reproduction and gamete transmission [6].

S. lewini is one of the most exploited and threatened sharks. Some reports~~a threatened shark species~~ mention~~,~~ ~~It~~around ~~has been estimated~~ ~~that~~ one to three million ~~specimens~~ sharks are killed annually worldwide because of fishing and the shark fin trade [7,8]. This species was considered to be underexploited in 1999~~;~~, however, ~~it is~~in 2009 the International Union for Conservation of Nature (IUCN) ~~currently l~~listed the species on the Red List with Endangered (EN) status [9]. Five years later ~~as an endangered species in the IUCN red list [7] and in~~ the ~~Appendix II of The~~ Convention on International Trade in Endangered Species (CITES) included hammerhead shark in Appendix II, and in 2019 the vulnerability status was raised to Critical Endangered (CR) [10].

High exploitation of the population of *S. lewini* may have an impact on population structure, reducing the fecundity of the species and the genetic diversity of its populations [11]. Hammerhead sharks are viviparous with a yolk-sac placenta which annual number of young 12-30 per litter. This species has a slow growth rate, late sexual maturity, long gestation period, and a long lifetime in nature [12-15]. The ~~High exploitation of the population of *S. lewini* may have an impact on population structure, reducing the fecundity of the species and the genetic diversity of its populations [8]. Furthermore~~Combination of high pressure from the capture fisheries and their biological characteristics make this species vulnerable to extinction. ~~, this species has a low resilience to exploitation because of its late sexual maturity and a long lifetime in nature [9, 10].~~

Formatted: Font: Times New Roman, 12 pt, No underline, Font color: Text 1

Formatted: Font: 12 pt

Formatted: Not Highlight

Formatted: Not Highlight

Formatted: Not Highlight

Formatted: Font: Times, 11 pt, Font color: Auto, English (United Kingdom)

The study of population genetics has become an important tool for understanding population connectivity, defining fisheries management, and conservation strategies ~~[11]~~. Genetic information can be used to define conservation unit and action plan through investigation on genetic and population structure of shark [16-19], tracing the source of populations supplying the international shark product trade particularly for *S. lewini* which one of the most abundant products in global market in Hongkong [20], and promote implement trade regulation of endangered species, sustainable shark fisheries and effective conservation [16,20,21]. The genetics of the population structure of *S. lewini*, which is important for fisheries stock management, has been widely investigated in different coastal areas and ocean basins on a global and regional scale [~~6,~~17-19,22,23 ~~12~~]. Duncan *et al*. [~~13~~22] reported a global phylogeographic study of *S. lewini* that indicated that the Indo-West Pacific region is the center of diversity for tropical sharks, such as *S. lewini*, with a high and unique genetic diversity; however, no samples from Indonesia were included in that study, or in the study reported by Ovenden *et al.* [~~14~~23], which included limited samples from Indonesia. ~~.~~

The aim of this study was to investigate the genetic diversity, population structure, and connectivity of *S. lewini* among populations in Indonesia, where the populations of this species are affected by fishing activities, and to observe the connectivity of this species at a regional scale in the Western Indian Ocean. Finally, we examined the implications of these results for species management and conservation.

Material and Methods

Ethics Statement

Formatted: Font: Italic

Commented [SH2]: Academic Editor:
explain how genetic information can be used to trave back the origin of shark find from this species found in markets, particularly in Asia. Cite Fields et al 2020, in Animal Conservation, a manuscript that came out this week.

Sutanto Hadi:
Thank you for your suggestion. We have added introduction in Line 53-67 and cited from Fields et al and Jabado et al in Line 68-75.

All specimens were already dead when collected, and no live specimens were obtained. Therefore, no approval from any institutional animal ethics committee was required. The sample collection and transportation were in accordance with the regulation of the Ministry of Marine Affairs and Fisheries of the Republic of Indonesia (Number 5/PERMEN-KP/2018) on the prohibition of cowboy shark and hammerhead shark export from Indonesia. This study was also approved by Ministry of Marine Affairs and Fisheries of the Republic of Indonesia, under permission numbers 276/BPSPL.03/PRL/X/2018 and 319/PNK/BPSPL.03/PK.230/REKOM/X/2018.

Tissue Sample Collection

A total of 100 tissue samples of *S. lewini* were collected from October 2017 to November 2018, including 41 samples from the Meulaboh and Aceh Jaya fishing ports (Aceh), 30 samples from a local shark landing in Manggar ~~Baru~~, Balikpapan, and 29 samples from the Tanjung Luar fishing port (Lombok) (Table 1). The samples (~0.5 cm3) were dissected from flesh or fin tissues and preserved in sample bottles containing 96% ethanol.

Table 1. Sampling collection sites at major shark landing sites in Indonesia

Site	Geographic coordinate	Number of samples
**Aceh (ACH)**		
Meulaboh	N 04° 08’ 29” E 96° 07’ 55”	33
Aceh Jaya	N 04° 38’ 34” E 95° 34’ 58”	8
**Balikpapan (BPN)**		
Manggar ~~Baru~~	S 01° 12’ 53” E 116° 58’ 24”	30
**Lombok (LOM)**		
Tanjung Luar	S 08° 46’ 39” E 116° 31’ 01”	29
**Total**		**100**

DNA Extraction, Amplification, and Sequencing

DNA ~~Extraction~~ extraction ~~of DNA~~ was performed at the Biodiversity and Biosystematics Laboratory, IPB University, according to the protocol of the gSYNC DNA extraction kit product. A fragment of the mitochondrial cytochrome oxidase subunit 1 (*CO1*) gene was amplified using the forward primer fish-BCL (5'–TCA ACY AAT CAY AAA GAT ATY GGC AC–3′) and the reverse primer fish-BCH (5'–ACT TCY GGG TGR CCR AAR AAT CA–3′) [~~15~~24, ~~16~~25~~]~~ ~~(Baldwin *et al.* 2009; Madduppa *et al.* 2016)~~] in a 24 µL reaction mixture consisting of 3 µL of DNA template, 12.5 µL of MyTaq HS Red Mix, 9 µL of ddH_2O, 1.25 µL each of forward and reverse primers. The reaction mixture was processed in a polymerase chain reaction (PCR) on a thermocycler using modified cycling conditions [~~17~~26,27 ~~18~~] ~~(Prehadi *et al.* 2015; Hadi *et al.* 2019)~~: pre-denaturation at 94°C for 15 s; followed by 38 cycles of denaturation at 94°C for 30 s, annealing at 50°C for 30 s, and extension at 72°C for 45 s; and a final extension at 72°C for 10 min. The amplicons were visualized by 1.5% agarose gel electrophoresis ~~smeared~~ added with ethidium bromide at 100 V for 20 min. The gel was observed under UV light to identify bands indicating the presence of DNA fragments. Sequencing was performed using sequencing machine with an optimized protocol of Sanger method [28].

All laboratory protocol both on sampling method and DNA identification were described in a short protocol deposited in protocols.io platform with digital object identifier (DOI) available at dx.doi.org/10.17504/protocols.io.bfwmjpc6. ~~Sequencing was performed using the BigDye Terminator v3.1 cycle sequencing kit with an optimized protocol of the Applied Biosystems 3730 genetic analyzer at the 1st BASE, Malaysia.~~

Data Analysis

Commented [SH3]: Academic Editor:
DNA extraction

Sutanto Hadi:
Thank you for your correction. We have fixed it.

Commented [SH4]: Sutanto Hadi
We have deposited a short laboratory protocol in protocols.io and added the DOI number at the end of Method section, Line 125-127.

Formatted: Default Paragraph Font, Font: Times New Roman, 12 pt, Font color: Text 1, Not Highlight

Formatted: Font: Times New Roman, 12 pt, Font color: Text 1

Formatted: Font: Times New Roman, 12 pt

Formatted: Font: Times New Roman, 12 pt, Font color: Text 1, Not Highlight

Formatted: Font: Times New Roman, 12 pt, Font color: Text 1

Formatted: Font: Times New Roman, 12 pt, Font color: Text 1

Formatted: Font: Times New Roman, 12 pt, Font color: Text 1

Formatted: Font color: Text 1

Formatted: English (United Kingdom)

Genetic Diversity

The sequences of 179 mitochondrial *CO1* DNA sequences with an average length of 594 bp were edited and aligned using the ClustalW algorithm [29] implemented in MEGA 6.06 [~~19~~30]. Genetic diversity parameters, including the number of haplotypes, haplotype diversity (h), and nucleotide diversity (π), were calculated using the DNASp v6 [~~20~~31] and Arlequin v.3.5 program [~~21~~32]. We also reanalyzed additional *CO1* sequencing data of *S. lewini* from West Papua retrieved from GenBank (Table 2) (n = 14), which were obtained by Sembiring *et al.* [~~22~~33], and sequences from previous studies performed in India (n = 6) [~~23~~34], the United Arab Emirates (n = 30) [~~24~~35], and Madagascar (n = 29) [~~25~~36], to assess the genetic diversity in Indonesian and Western Indian Ocean populations.

Table 2. Localities, total number (n), and accession number of *CO1* gene sequences of *S. lewini* from Aceh, Balikpapan, Lombok, and Western Papua (Indonesia) and India, the United Arab Emirates, and Madagascar (Western Indian Ocean)

Locality	n	Accession number	Author
**Indonesia**			

Aceh	41	MT324149-156, MT324187-219~~,~~ ~~MT324149-156~~	This study
Balikpapan	30	MT324157-186	This study
Lombok	29	MT324220-248	This study
West Papua	14	KF590254-55, KF590271-76, KF793729, KF793738-42	[~~22~~33]
**Western Indian Ocean**			
India	6	KF899746-51	[~~23~~34]
United Arab Emirates	30	KP177238-41, KP177241, KP177254, KP177262, KP177272, KP177285-99, KP177300-07	[~~24~~35]
Madagascar	29	HQ171735-47, HQ171761-76	[~~25~~36]

Population Structure

An analysis of molecular variance (AMOVA) and ~~Wright's~~ fixation index (F_{ST}) analysis [~~26~~37] were performed for three major group: 1) within and among the four populations from Indonesia, 2) within and among the three populations from the Western Indian Ocean, and 3) comparison between populations from Indonesia and Western Indian Ocean using the Arlequin v.3.5 program (set up, 1000 permutations; significance level threshold, α = 0.05), to estimate the overall extent of the genetic variation and differentiation level in Indonesia and the Western Indian Ocean. Population differentiation and its significance between sampling sites were also calculated with pairwise estimates [38-40]~~s~~. ~~The value of F_{ST} ranges from zero to one; a low level of differentiation was implied when the F_{ST} value was closer to zero; otherwise, a high level of differentiation was assumed when the value was closer to one [27, 28].~~

Commented [SH5]: Academic Editor:
I dont think you need to inlude this, as this is know by population geneticists and also because it will also depend of the probability of significance.

Sutanto Hadi:
Thank you for your suggestion. We agreed with your suggestion, we have omitted the sentence.

Genetic Connectivity

A haplotype network was constructed with a median-joining method in the Network v5.1.1.0 program [~~29~~41] for all haplotypes detected in Indonesia and the Western Indian Ocean, to obtain haplotype connectivity in a broader spatial connection in the regional area of the Indian Ocean. The distribution of haplotypes for each location was also provided in a proper map, to show the clear distributions of haplotypes and genetic connectivity among the populations under study.

Results

Genetic Diversity

All sequences of *S. lewini* obtained in this study were deposited in the BOLD System with the Barcode Index Number (BIN) registry of BOLD:AAA2403 and database of GenBank with accession numbers MT324149-248 (Table 2). A total of 179 sequences of 594 bp of the mitochondrial *CO1* gene of *S. lewini* obtained from three sampling sites (Aceh, Balikpapan, and Lombok) and additional sequences of samples from West Papua and the West Indian Ocean region were used to generate a total of 11 haplotype variations with 40 polymorphic loci (Table 3).

Table 3. Forty polymorphic loci of 11 haplotypes from 179 sequences of the mitochondrial *CO1* gene of *S. lewini* samples from four localities in Indonesia and three localities in the Western Indian Ocean region

**Haplotypes**	**Locus Position**																																							
	**1**	**15**	**24**	**27**	**33**	**45**	**48**	**63**	**69**	**105**	**120**	**138**	**141**	**156**	**165**	**174**	**186**	**198**	**229**	**279**	**289**	**303**	**306**	**325**	**339**	**342**	**346**	**366**	**396**	**420**	**453**	**456**	**459**	**483**	**495**	**498**	**522**	**531**	**534**	**543**
H1*	A	T	A	C	T	T	G	T	T	A	T	C	G	T	T	C	C	T	T	C	G	T	T	C	C	A	C	T	T	C	C	T	T	C	T	C	C	T	C	[illegible]
H2*	.	.	.	A	.	.	.	.	.	G	.	A	T	.	C	T	T	C	C	T	.	C	C	T	T	C	.	C	C	T	.	.	.	A	.	T	.	C	.	[illegible]
H3	.	.	.	A	.	.	.	.	C	G	.	A	T	.	C	T	T	C	C	T	.	C	.	T	T	C	.	C	C	T	.	.	.	A	.	T	.	C	.	[illegible]
H4*	.	.	.	A	.	.	.	.	.	G	.	A	T	.	C	T	T	C	C	T	.	C	C	T	T	C	.	C	C	T	.	.	.	A	.	T	.	C	.	[illegible]
H5	.	.	.	.	.	.	.	.	.	.	.	.	.	.	.	.	.	.	.	.	.	.	.	.	.	.	.	.	.	.	.	.	G	.	G	.	.	.	.	[illegible]
H6	.	.	.	.	.	C	.	.	.	.	.	.	.	.	.	.	.	.	.	.	.	.	.	.	.	.	.	.	.	.	.	.	.	.	.	.	.	.	.	[illegible]
H7	.	.	C	.	.	.	.	.	.	.	.	.	.	G	.	.	.	.	.	.	.	.	.	.	.	.	.	.	.	.	.	.	.	.	.	.	.	.	.	[illegible]
H8	.	C	.	.	C	.	.	.	.	.	.	.	.	.	.	.	.	.	.	.	.	.	.	.	.	.	.	.	.	.	.	.	.	.	.	.	.	.	.	[illegible]
H9	.	.	.	.	.	.	.	.	.	.	G	.	.	.	.	.	.	G	.		T	.	.	.	.	.	.	.	.	.	.	.	.	.	.	.	.	.	G	[illegible]
H10	G	.	.	.	.	.	.	G	.	.	.	.	.	G	.	.	.	.	.	.	.	.	.	.	.	.	.	.	.	.	.	.	.	.	.	.	.	.	.	[illegible]
H11	.	.	.	.	.	.	.	.	.	.	.	.	.	.	.	.	.	.	.	.	.	.	.	.	.	.	T	.	.	.	T	.	.	.	C	.	T	.	.	[illegible]

Notes:

Nucleobase at each position is given for H1 and only nucleobases that were different from H1 are written for all other haplotypes. Nucleobases identical to H1 are indicated with dots (.)

*Three original haplotypes of the *S. lewini* populations from the three areas of study (Aceh, Balikpapan, and Lombok) in Indonesia. The remaining haplotypes were reanalyzed from previous studies.

The comparison of the genetic diversity of *S. lewini* based on haplotype and nucleotide diversity revealed the presence of variation (Table 4). The haplotype diversity (h) among the localities in Indonesia ranged from 0.143 to 0.668 and the nucleotide diversity (π) ranged from 0.000 to 0.020. The highest genetic diversity in Indonesia was observed for the population from Aceh (h = 0.668; π = 0.020), followed by the Balikpapan population, which exhibited a lower haplotype and nucleotide diversity (h = 0.646; π = 0.002), and the lowest genetic diversity was detected in West Papua (h = 0.143, π = 0.000). The *S. lewini* population from Lombok also exhibited a fairly low genetic diversity (h = 0.362; π = 0.001). However, the overall genetic diversity in Indonesia was relatively high (h = 0.717; π = 0.013). Conversely, the average genetic diversity of the Western Indian Ocean region was low and ranged from 0.000 to 0.467. The overall diversity in the Western Indian Ocean region was also low (h = 0.232; π = 0.001).

Table 4. Genetic diversity of *S. lewini* based on sample size (n), haplotype number (Hn), haplotype diversity (h), and nucleotide diversity (π) in samples from each site in Indonesia and the Western Indian Ocean region

Population	n	Genetic Diversity		
		Hn	h	π
**Indonesia**				
Aceh (ACH)	41	4	0.668	0.020
Balikpapan (BPN)	30	3	0.646	0.002
Lombok (LOM)	29	3	0.362	0.001
West Papua (WEP)	14	2	0.143	0.000
Overall Indonesia	114	4	0.717	0.013
**Western Indian Ocean**				
India (IND)	6	1	0.000	0.000
United Arab Emirates (UAE)	30	8	0.467	0.002
Madagascar (MDG)	29	1	0.000	0.000
Overall Western Indian Ocean	65	8	0.232	0.001

Population Structure

The analysis of the fixation index (F_{ST}) and the corresponding *P*-values between and within the four *S. lewini* populations (ACH, BPN, LOM, and WEP) from Indonesia and the three populations (IND, UAE, and MDG) from the Western Indian Ocean region are shown in Table 5. The overall F_{ST} value in Indonesia was significantly higher than that observed in other regions (F_{ST} = 0.442; $P < 0.001$), which indicated the presence of multiple subdivisions in Indonesia.

~~A low F_{ST} value with n~~No significant ~~differences~~ differentiation was observed in the population from the Western Indian Ocean region (F_{ST} = –0.013; P = 0.601), while a comparison of the population structure between Indonesia and the Western Indian Ocean region yielded significant differentiation~~ces~~ (F_{ST} = 0.740; P < 0.001). The pairwise F_{ST} values between the populations from the four locations in Indonesia and a comparison with those from Western Indian Ocean population are shown in Table 6. The overall pairwise analysis via the distance method indicated the presence of significant differentiation ~~ces~~ among the four populations from Indonesia, with the exception of BPN and LOM (F_{ST} = 0.044; P = 0.090), which showed fairly low F_{ST} values and no significant p values. Furthermore, among the populations from Indonesia, the ACH population tended to be closer to the Western Indian Ocean population, as it exhibited a lower F_{ST} value (rather than F_{ST} range) compared with other localities from Indonesia to the Western Indian Ocean.

Commented [SH6]: Academic Editor:
No significant differentiation was observed

Sutanto Hadi:
Thank you for your correction. We have revised the sentence.

Commented [SH7]: Academic Editor:
significant differentiation

Sutanto Hadi:
Thank you for your correction. We have revised the phrase.

Commented [SH8]: Academic Editor:
differentiation

Sutanto Hadi:
Thank you for your correction. We have revised it.

Commented [SH9]: Academic Editor:
include number of Fst and p value

Sutanto Hadi:
Thank you for your suggestion. We have added value of Fst and p

Table 5. Analysis of molecular variance (AMOVA) for percentage of variation (%), F_{ST} value, and significance level (P-value) in *S. lewini* samples from Indonesian populations, Western Indian Ocean populations, and between Indonesian and Western Indian Ocean populations

Source of variation	df	Percentage of variation (%)	F_{ST} value	P value
**Indonesia**				
Among Populations	3	44.15	0.442	0.000
Within Populations	110	55.85		
Total	113			
**Western Indian Ocean**				
Among Populations	2	−1.31	−0.013	0.601
Within Populations	62	101.31		
Total	64			
**Indonesia vs. Western Indian Ocean**				
Among Populations	1	74.04	0.740	0.000
Within Populations	177	25.96		
**Total**	**178**			

Formatted: Indent: First line: 0.03"
Formatted: Indent: First line: 0.22"
Formatted: Indent: First line: 0.22"
Formatted: Indent: First line: 0.22"
Formatted: Indent: First line: 0.22"
Formatted: Indent: First line: 0.22"
Formatted: Indent: First line: 0.22"
Formatted: Indent: First line: 0.03"
Formatted: Indent: First line: 0.22"
Formatted: Indent: First line: 0.22"
Formatted: Indent: First line: 0.22"

Table 6. Pairwise F_{ST} values (below the diagonal) and P-values (above the diagonal) between the *S. lewini* populations from Aceh (ACH), Balikpapan (BPN), Lombok (LOM), West Papua (WEP), and Western Indian Ocean (WIO)

Sample Sites	ACH	BPN	LOM	WEP	WIO
**ACH**	-	0.000	0.000	0.000	0.000
**BPN**	0.427	-	0.090~~1~~	0.001	0.000
**LOM**	0.438	0.044	-	0.000	0.000
**WEP**	0.398	0.495	0.736	-	0.000
**WIO**	0.509	0.965	0.973	0.975	-

Genetic Connectivity

A haplotype network analysis identified two main groups of haplotypes (Fig. 1), referred to as Clade A and Clade B henceforth. Clade A consisted of haplotype H1, which was observed in several regions, i.e., Aceh, India, United Arab Emirates, and Madagascar, whereas H5, H6, H7, H8, H9, H10, and H11 were present only in the samples from the United Arab Emirates. Clade B consisted of three haplotypes (H2, H3, and H4), which were spread evenly in Indonesia, although each region exhibited predominant haplotypes. Haplotypes H1, H2, H4, and H3 were predominant in Aceh, Balikpapan, Lombok, and West Papua, respectively (Fig. 2).

Fig 1. Haplotype network of the *S. lewini* (n = 179) population from Indonesia and the Western Indian Ocean region, which was constructed using the Median Joining method

Fig 2. Distribution of the 11 haplotypes of the *S. lewini* population from Indonesia and Western Indian Ocean at the regional scale

Discussion

Genetic Diversity

The overall genetic diversity of *S. lewini* at the haplotype and nucleotide levels was relatively high for populations in Indonesia. These findings are consistent with the results reported by Ovenden *et al.* [~~14~~23], pertaining to the mitochondria control region of *S. lewini* from three localities in Indonesia, including Lombok, and is ~~the~~ a highly genetic diversity of *S. lewini* observed at the global scale [~~13~~22]. The scalloped hammerhead

Commented [SH10]: Academic Editor:
is a highly

Sutanto Hadi:
Thank you for your correction. We have revised the phrase.

sharks are a highly migratory species with a wide distribution in tropical and warm-temperate waters and possibly move across oceanic waters to a distance of up to ~~1500~~ 1671 km [~~9~~12]. In principle, because of migration capability and broad ecological niches, this species tends to have a higher genetic diversity than do species from narrow ecological niches [~~3~~42~~0~~]. High genetic diversity values are commonly related to large population sizes [~~31~~43] and are promoted by several factors, such as local population sizes, fast generation times [~~32~~44], high nucleotide substitution rates [~~33~~45], and high gene flow between geographically distant populations.

The finding of a relatively high genetic diversity for *S. lewini* in Indonesia appears to be inconsistent with the general assumption that overexploitation of this species as both a target of fishing and bycatch has led to the decline of its populations worldwide [~~34~~46]. However, the results obtained here for the population from Lombok may be relevant in this context; i.e., the lower genetic diversity detected at that location was probably driven by continuous fishing pressure. *S. lewini* were the top three targeted sharks at the Tanjung Luar fishing port in Lombok and have faced high fishing pressure over more than 40 years, which has led to its overfishing, with recent exploitation rates (E) reaching 0.59 [~~35, 36~~47].

Furthermore, according to ~~the global information system statistic of the FAO on fisherie~~the global fisheries information system on hammerhead shark capture production 1950-2017 on global scale and Indonesia by FAO ~~s~~ [~~37~~48], the hammerhead sharks (Sphyrnidae), including *S. lewini*, are important sharks that were highly exploited in the last two decades, with an estimated rapid increase in global capture, from 220 ton per year in 1985 up to approximately 10,362 tons per year in 2016. In the same period, the capture level in Indonesia also increased significantly, reaching approximately 1,492 tons

Commented [SH11]: Academic Editor:
what type of fisheries, be more specific

Sutanto Hadi:
Thank you for your suggestion. We have added more specific information.

in 2016. Moreover, Indonesia exhibited one of the highest volumes of sharks and rays caught on the global catches reported in the period of 2000–2011 [~~38~~49].

Clarke *et al*. [~~39~~50] reported similar findings regarding the mitochondrial DNA of the silky shark, *Carcharhinus falciformis*, which exhibited a high genetic diversity under circumstances of overexploitation. Elasmobranchs exhibit adaptability to environmental and anthropogenic stresses, which causes genetic bottlenecks because of their particular life histories [~~40~~51]. Moreover, the population decline caused by recent fishery activities might be insufficient to reduce genetic diversity, particularly for species with a ~~long life~~long-life span (13–20 years), including *S. lewini* [~~41~~52, ~~42~~53].

However, the continuous decline in these populations, as was expected to occur for the *S. lewini* population from Lombok, was positively correlated with the loss of genetic diversity and created a population bottleneck [~~42~~53, ~~43~~54], as reported by Pinsky and Palumbi ~~[44]~~ in a meta-analysis of several marine fish [55].

Population Structure

Our findings indicated the presence of homogeneity among the *S. lewini* populations from Balikpapan and Lombok. The pairwise F_{ST} analysis detected no significant genetic differentiation in these two populations with lowest value of F_{ST}. These finding complements those of previous studies conducted in Indo-Australian waters. Ovenden *et al.* [~~14~~23] reported similar evidence for the mitochondrial control region regarding population structure, with no differentiation between two populations of *S. lewini* (Lombok and northern Australia). This pattern of single-stock population seems to suggest that these localities are a migration zone of *S. lewini* and that a reproductive

movement might occur in the coastal areas of these locations, although there are strong Indonesian throughflow currents between Kalimantan and Sulawesi island. Adult *S. lewini* specimens are highly migratory, with a large body size that supports the high dispersal capacity of this species; hence, they exhibit the possibility of overcoming that geographical barrier. In contrast, we obtained different results from the comparison among these two populations from central Indonesia and one population from Aceh (western Indonesia) and one population from West Papua (eastern Indonesia), which exhibited strong genetic differentiation. These regions were spatially separated by a long distance, with the possibility of the existence of more complex barriers, both inter-island barriers and anthropogenic factors pertaining to a high commercial and artisanal fishing pressure, such as those found along the southern and northern coasts of Java.

Genetic Connectivity

Regarding significant value of F_{ST} among population (F_{ST} = 0.740; P < 0.001) and haplotype network and distribution shown in Fig 1 and 2, we observed two restricted haplogroup which separated *S. lewini* population in Indonesian with Western Indian Ocean population. Those two haplogroup were separated with 19 different nucleotide bases as result of monomorphic and polymorphic mutation. Limited gene flow that occurs only among populations in Indonesia forms a different gene pool with Western Indian Ocean population. It can be expected due to a complex geographic barrier in Indonesia's marine ecosystem, and confirmed by global distribution pattern of S. lewini, with significant separation population across ocean basin and discontinuous coastlines habitat [6].

Formatted: Font: Italic

Overall, in Indonesia, *S. lewini* populations from Balikpapan, Lombok, and West Papua seemed to be isolated from the population from the Western Indian Ocean, and shared a haplotype network exclusively only in eastern Indonesian waters. However, ~~W~~we obtained an interesting result regarding haplotype sharing between the populations from ~~Indonesia~~ Aceh and the Indian Ocean population. ~~.~~ H1 was a unique haplotype found in Aceh, Indonesia, and was correlated with the most common haplotype observed in samples from the three locations of the Western Indian Ocean region (India, United Arab Emirates, and Madagascar), which indicates the presence of connectivity between the populations from Aceh and those from the Western Indian Ocean region. Schott and McCreary [~~45~~56] reported that the Indian Ocean seasonal current carried water from north Sumatra through the Arabian Sea to Madagascar, which represents a possible connectivity channel between the two regions, thus allowing *S. lewini* to migrate and leading to ~~cross-~~breeding and gene flow. The similarity between the predominant haplotype (H1) of *S. lewini* from Aceh with that of the populations from India, United Arab Emirates, and Madagascar evidenced a genetic sharing process among populations in the Indian Ocean region. However, the *S. lewini* populations from Balikpapan, Lombok, and West Papua seemed to be isolated from the population from the Western Indian Ocean, and shared a haplotype network exclusively in eastern Indonesian waters.

Conservation Implications

The overall high genetic diversity of the *S. lewini* populations in Indonesia suggests that this species has not yet experienced a genetic loss because of exploitation pressure. However, the lower genetic diversity of *S. lewini* from Lombok and West Papua indicated a higher risk of loss of genetic diversity, which probably was the result of high fisheries

Commented [SH12]: Academic Editor:
you have many nucleotide changes between your haplotypes from Indonesia and those from the Weste Indian Ocean...what could be happening there? is there really gene flow with the West Indian Ocean? I think gene flow will be restricted, otherwise you would not find such differentiated haplotypes. Re -think this section and go deeper into your results

Sutanto Hadi:
Thank you for your correction. We have stated some sentences in Line 318-330 regarding haplotype differentiation between two haplogroup between Indonesia and western Indian Ocean, and we also highlight in next sentence 331-347, an indication on genetic sharing between population in Aceh and Western Indian Ocean which have similarity in H1 (haplotype one).

Commented [SH13]: Academic Editor:
and gene flow

Sutanto Hadi:
Thank you for your correction. We have added the phrase.

pressure. Our genetic assessment of *S. lewini* samples from four localities revealed that a single stock ~~exist~~exists between Lombok and Balikpapan, while a separate stock was observed for Aceh and West Papua, indicating that the management of this species should occur on a stock-based approach at least on three mitochondrial-stock conservation management units. The complex population of *S. lewini* with limited connectivity observed in Indonesia and the Western Indian Ocean region indicated the importance of promoting collaborative management strategies among Indonesian management agencies and in conjunction with Western Indian Ocean agencies at the regional scale.

Conclusion

This study provided important findings regarding the population structure of *S. lewini* in Indonesia, with a high genetic diversity and three significant subdivisions that indicated the capability of the population to adapt to rapid environmental changes and pressure, including fishing activities. However, the lower genetic diversity detected in Lombok and West Papua should be considered. The genetic sharing detected among the populations from Indonesia and also those from the Western Indian Ocean proved the connectivity among these populations. A collaborative action across regions is needed to promote sustainable management and conservation purposes, both in Indonesia and at the regional scale in the Western Indian Ocean area.

Acknowledgement

We would like to thank to the institutions and individuals that have made our study possible: colleagues from Marine Biodiversity and Biosystematics Lab (BIODIVSI), Faculty of Marine Science and Technology, IPB University for their help during research.

We also appreciated to BPSPL Satker Balikpapan, Listian Nova and Mr. Hery, Head of TPI Manggar, Balikpapan for their help during collecting samples, and to reviewers and proof readers who have provided highly constructive suggestions for better writing of the paper.

References

1. Klimley AP, Butler SB. Immigration and emigration of a pelagic fish assemblage to seamounts in the Gulf of California related to water mass movements using satellite imagery. Mar Ecol Prog Ser. 1988;49: 11-20.
2. Compagno LJV. FAO Species Catalogue: Sharks of the world. Rome: Food ~~And~~ and Agriculture Organization;1984.
3. Montgomery JC, Walker MM. Orientation and navigation in elasmobranchs: which way forward? Environ Biol Fishes. 2001;60: 109-116.
4. Kajiura SM, Holland KN. Electroreception in juvenile scalloped hammerhead and sandbar sharks. J Exp Biol. 2002;205: 3609-3621.
5. Meyer CL, Holland KN, Papastamatiou YP. Sharks can detect changes in the geomagnetic field. J R Soc Interface. 2005;2: 129-130.

~~6.~~ Daly-Engel TS, Seraphin KD, Holland KN, Coffey JP, Nance HA, Toonen RJ, et al. Global phylogeography with mixed-marker analysis reveals male-mediated dispersal in the endangered scalloped hammerhead shark (*Sphyrna lewini*). PLoS ONE. 2012;7(1): e29986. doi: 10.1371/journal.pone.0029986.

6.

Formatted: Font color: Text 1, (Asian) Korean

7. Clarke SC, McAllister MK, Milner-Gulland EJ, Kirkwoo GP, Michielsens CGJ, Agnew DJ et al. Global estimates of shark catches using trade records from commercial markets. Ecol Lett. 2006;9(10): 1115-1126.

~~7.~~ Chapman DD, Simpfendorfer CA, Wiley TR, Poulakis GR, Curtis C, Tringali M et al. Genetic diversity despite population collapse in a critically endangered marine fish: the smalltooth sawfish (*Pristis pectinata*). J Hered. 2011;102: 643-652.

8.

9. Baum J, Clarke S, Domingo A, Ducrocq M, Lamónaca AF, Gaibor N et al. Sphyrna lewini. The IUCN Red List of Threatened Species 2009. 2009; e.T39385A10190088.

10. Rigby CL, Dulvy NK, Barreto R, Carlson J, Fernando D, Fordham S et al. Sphyrna lewini. The IUCN Red List of Threatened Species 2019. 2019; e.T39385A2918526.

11. Ward RD. Genetics in fisheries management. Hidrobiologia. 2000;420: 191–201.

~~8.~~1. ~~Castillo-olguin E, Aribe-Alcocer M, Diaz-Jaimes P. Assessment of the population genetic structure of *Sphyrna lewini* to identify conservation units in the Mexican Pacific. Ciencias Marinas. 2012;38(4): 635-652. doi: 10.7773/cm.v38i4.2110.~~

~~9.~~1. ~~Ward RD. Genetics in fisheries management. Hidrobiologia. 2000;420: 191–201.~~

12. Kohler NE, Turner PA. Shark tagging: a review of conventional methods and studies. Environ Biol Fishes. 2001;60: 191-223.

13. White W, Barton C, Potter I. Catch composition and reproductive biology of *Sphyrna lewini* (Griffith & Smith) (Charcharhinifirnes, Sphyrnidae) in Indonesian waters. J Fish Biol. 2008;72: 1675-1689.

~~10.~~14. Ovenden J, Morgan JT, Street R, Tobin A, Simfendorfer CA, Macbeth W, Welch D. Negligible evidence for regional genetic population structure for two shark species

Formatted: Font: Times New Roman, 12 pt

Formatted: List Paragraph, Justified, Indent: Left: 0", Hanging: 0.3"

Formatted: Font: Times New Roman, 12 pt

Formatted: Font color: Text 1

Formatted: Font: Times New Roman, 12 pt, English (United Kingdom)

Formatted: Font: Times New Roman

Formatted: English (United Kingdom)

Formatted: Font:

Formatted: Font: Times New Roman, 12 pt, Font color: Text 1

Formatted: List Paragraph, Justified, Indent: Left: 0", Hanging: 0.3", Numbered + Level: 1 + Numbering Style: 1, 2, 3, … + Start at: 1 + Alignment: Left + Aligned at: 0.25" + Indent at: 0.5"

Formatted: Font: Times New Roman, 12 pt, Italic, Font color: Text 1

Formatted: Font: Times New Roman, 12 pt, Font color: Text 1

Rhizoprionodon acutus (Rüppell, 1837) and *Sphyrna lewini* (Griffith & Smith, 1834) with contrasting biology. Mar Biol. 2011;158: 1497-1509.

~~11.~~15. Bessudo S, Soler GA, Kimley PA, Ketchum J, Arauz R, Hearn A, et al. Vertical and horizontal movements of scalloped hamemerhead shark (*Sphyrna lewini*) around Malpelo and Cocos islands (Tropical Eastern Pacific) using satellite telemetry. Boletín de Investigaciones Marinas y Costeras*.* 2011;40: 91-106.

~~12.~~16. Cardeñosa D, Hyde J, Caballero S. Genetic diversity and population structure of the pelagic thresher shark (*Alopias pelagicus*) in the Pacific Ocean: evidence for two evolutionarily significant units. PLoS ONE. 2014;9(10): e110193. doi: 10.1371/journal.pone.0110193.~~.~~

17. Castillo-olguin E, Aribe-Alcocer M, Diaz-Jaimes P. Assessment of the population genetic structure of *Sphyrna lewini* to identify conservation units in the Mexican Pacific. Ciencias Marinas. 2012;38(4): 635-652. doi: 10.7773/cm.v38i4.2110.

18. Quintanilla S, Gómez A, Mariño-Ramírez C, Sorzano C, Bessudo S, Soler G, et al. Conservation genetics of the scalloped hammerhead shark in the Pacific Coast of Colombia. J Hered. 2015;106: 448-458. doi:10.1093/jhered/esv050.~~.~~

19. Sukumaran S, Sebastian W, Mukundan LP, Menon M, Akhilesh K V., Zacharia PU, et al. Molecular analyses reveal a lack of genetic structuring in the scalloped hammerhead shark, Sphyrna lewini (Griffith & Smith, 1834) along the Indian coast. Mar Biodivers. 2020;50. doi:10.1007/s12526-020-01040-4.

20. Fields AT, Fischer GA, Shea SKH, Zhang H, Feldheim KA, Chapman DD. DNA Zip- coding: identifying the source populations supplying the international trade of a critically endangered coastal shark. Animal Conservation. 2020; doi:10.1111/acv.12585.

Formatted: Font: Italic

Formatted: Font: Italic

Formatted: Font color: Text 1

Formatted: Font color: Text 1

Formatted: Font: Times New Roman, 12 pt

Formatted: Font: Times New Roman, 12 pt

Formatted: Font color: Text 1

21. Jabado RW, Al Ghais SM, Hamza W, Henderson AC, Spaet JLY, Shivji MS, et al. The trade in sharks and their products in the United Arab Emirates. Biol Conserv. 2015;181: 190–198. doi:10.1016/j.biocon.2014.10.032

22. Duncan KM, Martin AP, Bowen BW, De Coute HG. Global phylogeography of the scalloped hammerhead shark (Sphyrna lewini). Mol Ecol. 2006;15:2239-51.

~~13.~~

~~14.~~1. ~~Duncan KM, Martin AP, Bowen BW, De Coute HG. Global phylogeography of the scalloped hammerhead shark (Sphyrna lewini). Mol Ecol. 2006;15:2239-51.~~

~~15.~~23. Ovenden JR, Kashiwagi T, Broderick D, Giles J, Salini J. The extent of population genetic subdivision differs among four co-distributed shark species in the Indo-Australian archipelago. BMC Evol Biol. 2009;9: 40. doi:10.1186/1471-2148-9-40.

~~16.~~24. Baldwin CC, Mounts JH, Smith DG, Weigt LA. Genetic identification and color descriptions of early life-history stages of Belizean *Phaeoptyx* and *Astrapogon* (Teleostei: Apogonidae) with comments on identification of adult *Phaeoptyx*. Zootaxa. 2009;2008: 1-22.

~~17.~~25. Madduppa H, Ayuningtyas RU, Subhan B, Arafat D. Exploited but unevaluated: DNA barcoding reveals skates and stingrays (Chordata, Chondrichthyes) species landed in the Indonesian fish market. IJMS. 2016;21: 77-84.

~~18.~~26. Prehadi, Sembiring A, Kurniasih EM, Rahmad, Arafat D, Subhan B et al. DNA barcoding and phylogenetic reconstruction of shark species landed in Muncar fisheries landing site in comparison with Southern Java fishing port. Biodiversitas. 2015;16: 55-61.

~~19.~~27. Hadi S, Anggraini NP, Muttaqin E, Simeon BM, Subhan B, Madduppa H. Genetic diversity of the endangered species Sphyrna lewini (Griffith and Smith 1834) in

Formatted: Justified, Indent: Left: 0", Hanging: 0.3"

Formatted: Font: Times New Roman, 12 pt, Font color: Text 1

Lombok based on mitochondrial DNA. IOP Conf. Ser.: Earth Environ. Sci. 2019;236: 012024.

28. Sanger F, Nicklen S, Coulson AR. DNA sequencing with chain-terminating inhibitors. Proc Natl Acad Sci. 1977;74(12): 5463-5467. doi:10.1073/pnas.74.12.5463.

29. Thompson JD, Higgins DG, Gibson TJ. CLUSTAL W: improving the sensitivity of progressive multiple sequence alignment through sequence weighting, position-specific gap penalties and weight matrix choice. Nucleic Acids Res. 1994;22(22): 4673-4680.

~~20.~~30. Tamura K, Stecher G, Peterson D, Filipski A, Kumar S. Molecular Evolutionary Genetics Analysis Version 6.0. Mol Biol Evol. 2013;30(12): 2725-2729. doi: 10.1093/molbev/mst197.

~~21.~~31. Rozas J, Ferrer-Mata A, Sánchez-DelBarriol JC, Guirao-Rico S, Librado P, Ramos-Onsins SE. DnaSP v6: DNA Sequence polymorphism analysis of large datasets. Mol Biol Evol. 2017;34: 3299-3302.

~~22.~~32. Excoffier L, H.E. L. Lischer. Arlequin suite ver 3.5: A new series of programs to perform population genetics analyses under Linux and Windows. Mol Ecol Resour. 2010;10: 564-567.

~~23.~~33. Sembiring A, Pertiwi NPD, Mahardini A, Wulandari R, Kurniasih EM, Kuncoro AW, et al. DNA barcoding reveals targeted fisheries for endangered sharks in Indonesia. Fisheries Research. 2015;164: 130-134. doi: 10.1016/j.fishres.2014.11.003.

~~24.~~34. Bineesh KK, Gopalakrishnan A, Akhilesh KV, Sajeela KA, Abdussamad EM, Pillai NGK, et al. DNA barcoding reveals species composition of sharks and rays in

Formatted: Indent: Left: 0", Hanging: 0.3", Line spacing: Double, Tab stops: Not at 0.39"

the Indian commercial fishery. Mitochondrial DNA. 2016. doi: 10.3109/19401736.2015.1137900.

~~25.~~35. Jabado RW, Ghais SMA, Hamza W, Henderson AC, Spaet JLY, Shivji MS, et al. The trade in sharks and their products in the United Arab Emirates. Biol Conserv. 2015;181: 190-198. doi: 10.1016/j.biocon.2014.10.032.

~~26.~~36. Doukakis P, Hanner R, Shivji M, Bartholomew C, Chapman D, Wong E, et al. Applying Genetic Techniques to Study Remote Shark Fisheries in Northeastern Madagascar. Mitochondrial DNA. 2011;22(S1): 15-20.

37. Wright S. The genetical structure of populations. Annals of Eugenics. 1951;15: 323-354.

38. Weir BS, Cockerham CC. Estimating F-Statistics for the Analysis of Population Structure. Evol. 1984;38(6): 1358. doi:10.2307/2408641.

39. Excoffier L, Smouse PE, Quattro JM. Analysis of molecular variance inferred from metric distances among DNA haplotypes: application to human mitochondrial DNA restriction data. *Genetics.* 1992;131(2): 479- 491.

40. Weir BS. Genetic Data Analysis II: Methods for Discrete Population Genetic Data. Sunderland, Mass: Sinauer Associates; 1996.

~~27.~~

~~28. Wright S. Evolution and the Genetics of Populations. 4th vol. Variability within and among Natural Populations. Chicago: University of Chicago Press; 1978.~~

~~29. Hartl DL, Clark AG. Principles of Population Genetics. 3nd ed. Sunderland: Sinauer Associates Inc; 1997.~~

~~30.~~41. Bandelt H-J, Forster P, Röhl A. Median-joining networks for inferring intraspecific phylogenies. Mol Biol Evol. 1999;16: 37-48.

Formatted: Font: Times New Roman

Formatted: Pattern: Clear

Formatted: Font: Times New Roman, 12 pt

Formatted: Font color: Text 1

Formatted: Font: Times New Roman, 12 pt

Formatted: Font color: Text 1

Formatted: Font: Times New Roman, 12 pt

Formatted: Indent: Left: 0", Hanging: 0.3"

Formatted: Font: Times New Roman, 12 pt

Formatted: Font: Times New Roman, 12 pt, Font color: Text 1

~~31.~~42. Habel JC, Schmitt T. Vanishing of the common species: Empty habitats and the role of genetic diversity. Biol. Conserv. 1996;218: 211-216. doi: 10.1016/j.biocon.2017.12.018.

~~32.~~43. Frankham R. Relationship of Genetic Variation to Population Size in Wildlife. Conserv. Biol. 1996;10(6): 1500-1508. doi:10.1046/j.1523-1739.1996.10061500.x.

~~33.~~44. Hague MTJ, Routman EJ. Does population size affect genetic diversity? A test with sympatric lizard species. Heredity. 2016;116: 92-98. doi: 10.1038/hdy.2015.76.

~~34.~~45. Martin AP, Naylor GJP, Palumbi SR. Rates of mitochondrial DNA evolution in sharks are slow compared with mammals. Nature. 1992;357: 153-157. doi: 10.1038/357153a0.

~~35.~~46. Compagno LJV, Dando M, Fowler SL. Sharks of the World. Princeton: Princeton University Press; 2005.

~~36. Hordyk A, Ono K, Valencia S, Loneragan N, Prince J. A novel length-based empirical estimation method of spawning potential ratio (SPR), and tests of its performance, for small-scale, data-poor fisheries. ICES J Mar Sci. 2015;2-15. doi: 10.1093/icesjms/fsu004.~~

~~37.~~47. Simeon BM, Agustina S, Muttaqin E, Yulianto I, Ichsan M, Muhsin M. Laporan teknis: Pemanfaatan Hasil Tangkap Hiu dan Pari di Tanjung Luar, Provinsi Nusa Tenggara Barat. Bogor: Wildlife Conservation Society-Indonesia Program; 2017.

~~38.~~48. Food and Agriculture Organization (FAO). Fisheries and Aquaculture Information and Statistics Service: Global Capture Production 1950-2017; Database: Indonesian hammerhead shark capture 2017 [cited 2020 Mar 5]. ~~Database: Indonesian hammerhead shark capture [Internet].~~Available from: http://www.fao.org/fishery/statistics/global-capture-production/query/es.

39.49. Dent F, Clarke. State of the Global Market for Shark Products. Rome: Food And and Agriculture Organization; 2015.

40. Clarke CR, Karl SA, Horn RL, Bernard AM, Lea JS, Hazin FH et al. Global mitochondrial DNA phylogeography and population structure of the silky shark, *Carcharhinus falciformis.* Mar Biol. 2015;162(5): 945-955. doi: 10.1007/s00227-015-2636-6

50. .

51. Chapman DD, Simpfendorfer CA, Wiley TR, Poulakis GR, Curtis C, Tringali M et al. Genetic diversity despite population collapse in a critically endangered marine fish: the smalltooth sawfish (*Pristis pectinata*). J Hered. 2011;102: 643-652.

41.1. Chapman DD, Simpfendorfer CA, Wiley TR, Poulakis GR, Curtis C, Tringali M et al. Genetic diversity despite population collapse in a critically endangered marine fish: the smalltooth sawfish (*Pristis pectinata*). J Hered. 2011;102: 643-652.

42.52. Sánchez-de Ita JA, Quiñónez-Velázquez C, Galván-Magaña F, Bocanegra-Castillo N, Félix-Uraga R. Age and growth of the silky shark *Carcharhinus falciformis* from the west coast of Baja California Sur, Mexico. J App Ichthyol. 2011;27: 20-24.

43.53. Nei M, Maruyama T, Chakraborty R. The bottleneck effect and genetic variability in populations. Evolution. 1975;29(1): 1. doi:10.2307/2407137.

44.54. Beebee T, Rowe G. An Introduction to Molecular Ecology. 2nd ed. New York: Oxford University Press Inc; 2008.

45.55. Pinsky ML, Palumbi SR. Meta-analysis reveals lower genetic diversity in overfished populations. Mol Ecol. 2013;23(1): 29-39. doi:10.1111/mec.12509.

Formatted: Font color: Text 1

Formatted: Font: Times New Roman, 12 pt

Formatted: Indent: Left: 0", Hanging: 0.3"

~~46.~~ Schott FA, McCreary JP. The monsoon circulation of the Indian Ocean. Prog Oceanogr. 2001;51: 1-123. doi: 10.1016/S0079-6611(01)00083-0.

56.

Formatted: List Paragraph, Justified, Indent: Left: 0", Hanging: 0.3", Line spacing: Double, Numbered + Level: 1 + Numbering Style: 1, 2, 3, … + Start at: 1 + Alignment: Left + Aligned at: 0.25" + Indent at: 0.5"

To:
Susana Caballero, PhD
Academic Editor
PLOS ONE

Thank you for reviewing and suggesting our manuscript:

"Genetic connectivity and diversity of endangered species the scalloped hammerhead shark Sphyrna lewini (Griffith & Smith 1834) population across Indonesia and the Western Indian Ocean"

I would like to response your comments in the following systematic table format:

1st SUBMISSION RESPONSE			
**No.**	**REVIEWER COMMENT**		**AUTHOR RESPONSES**
	**Additional Editor Comments (if provided)**		
1.	This is a very interesting manuscript and it present very important information that will help understanding the genetic diversity of scalloped hammerhead in this region and this information will be likely used for future assignment comparisons for example for market surveys etc. However, the English NEEDS to be improved. I really hope the authors can find the help of a native English speaker that can help them with this. I wrote some things in the PDF I am attaching but it definitely needs an overall improvement of the English		Thank you for your interest and suggestion. We have improved overall of the English for better manuscript after proof read by professional native English speaker.
2.	The resolution of the figures needs to be improved because they look grainy		Thank you for your suggestion. We have improved the resolution of figures and generated in PACE to meet PLOS ONE requirement.
3.	We would appreciate receiving your revised manuscript by 20th of April. When you are ready to submit your revision, log on to https://www.editorialmanager.com/pone/ and select the 'Submissions Needing		

	Revision' folder to locate your manuscript file.<br>If you would like to make changes to your financial disclosure, please include your updated statement in your cover letter.		
4.	To enhance the reproducibility of your results, we recommend that if applicable you deposit your laboratory protocols in protocols.io, where a protocol can be assigned its own identifier (DOI) such that it can be cited independently in the future. For instructions see: ttp://journals.plos.org/plosone/s/submission-guidelines#loc laboratory-protocols		
5.	Please include the following items when submitting your revised manuscript:<br>• A rebuttal letter that responds to each point raised by the academic editor and reviewer(s). This letter should be uploaded as separate file and labelled **'Response to Reviewers'**.<br>• A marked-up copy of your manuscript that highlights changes made to the original version. This file should be uploaded as separate file and labelled **'Revised Manuscript with Track Changes'**.<br>•An unmarked version of your revised paper without tracked changes. This file should be uploaded as separate file and labelled **'Manuscript'**.		
**No.**	**Journal Requirements**		**Author Responses**
1.	When submitting your revision, we need you to address these additional requirements:<br>Please ensure that your manuscript meets PLOS ONE's style requirements, including those for file naming. The PLOS ONE style templates can be		Thank you for your suggestion. We have read and followed the format as The PLOS ONE style templates.

	found at http://www.plosone.org/attachments/PLOSOne_formatting_sample_main_body.pdf and http://www.plosone.org/attachments/PLOSOne_formatting_sample_title_authors_affiliations.pdf		
2.	In your Methods section, please provide additional location information of the sampling collection sites, including geographic coordinates for the data set if available.		Thank you for your suggestion. We have provided additional location information including geographic coordinates in a table in manuscript.
3.	We suggest you thoroughly copyedit your manuscript for language usage, spelling, and grammar. If you do not know anyone who can help you do this, you may wish to consider employing a professional scientific editing service.		Thank you for your suggestion. We have sent and proof read our manuscript to a professional scientific editor to check and enhance our manuscript grammar, spelling and language use.
4.	Upon resubmission, please provide the following:<br>• The name of the colleague or the details of the professional service that edited your manuscript<br>• A copy of your manuscript showing your changes by either highlighting them or using track changes (uploaded as a *supporting information* file)<br>• A clean copy of the edited manuscript (uploaded as the new *manuscript* file)		
5.	Thank you for stating the following in the Acknowledgments Section of your manuscript:<br>"This research was supported by Wildlife Conservation Society (WCS)-Indonesia, in collaboration with Faculty of Fisheries and Marine Sciences IPB University. Sutanto Hadi received a scholarship from Indonesia Endowment Fund for Education (LPDP)."		Thank you for your suggestion. We have removed any funding-related statement from the manuscript.

	We note that you have provided funding information that is not currently declared in your Funding Statement. However, funding information should not appear in the Acknowledgments section or other areas of your manuscript. We will only publish funding information present in the Funding Statement section of the online submission form.<br>Please remove any funding-related text from the manuscript and let us know how you would like to update your Funding Statement. Currently, your Funding Statement reads as follows:<br>"NO - The funders had no role in study design, data collection and analysis, decision to<br>publish, or preparation of the manuscript."		
6.	PLOS requires an ORCID iD for the corresponding author in Editorial Manager on papers submitted after December 6th, 2016. Please ensure that you have an ORCID iD and that it is validated in Editorial Manager. To do this, go to ‘Update my Information’ (in the upper left-hand corner of the main menu), and click on the Fetch/Validate link next to the ORCID field. This will take you to the ORCID site and allow you to create a new iD or authenticate a pre-existing iD in Editorial Manager.<br>Please see the following video for instructions on linking an ORCID iD to your Editorial Manager account: https://www.youtube.com/watch?v=_xcclfuvtxQ<br>[Note: HTML markup is below. Please do not edit.]		

**2nd SUBMISSION RESPONSE**			
**No.**	**Reviewers' comments**		**Author Responses**
1.	While revising your submission, please upload your figure files to the Preflight Analysis and Conversion Engine (PACE) digital diagnostic tool, https://pacev2.apexcovantage.com/. PACE helps ensure that figures meet PLOS requirements. To use PACE, you must first register as a user. Registration is free. Then, login and navigate to the UPLOAD tab, where you will find detailed instructions on how to use the tool. If you encounter any issues or have any questions when using PACE, please email us at figures@plos.org. Please note that Supporting Information files do not need this step.		Thank you for your suggestion. We have uploaded and adjusted the pictures to PACE to meet PLOS ONE figures requirement. We attached the picture in submission.
2.	which populations have been declining worldwide		Thank you for your correction, we have revised the sentence
3.	use h instead of Hd		Thank you for your correction, we have replaced all of term “Hd” with “h”
4.	is considered coastal due to their need for nursery areas		Thank you for your suggestion, we used term “semi-oceanic” as stated in some journals which have the similar meaning that you suggested, so we agreed and have revised the sentence
5.	shorter phrases (Line 47-51)		Thank you for your correction. We have shortened the phrases.
6.	were already dead when collected		Thank you for your correction. We have revised the sentence.
7.	sample (Line 83)		Thank you for your correction
8.	100 samples of which length?		Thank you for your question, we used 594 bp of nucleotide and 179 sequences in total, not 100. We have revised it in the sentence
9.	you previously mention a 100 (sequences)		Thank you for your correction. We used 179 sequences in total, we have revised in previous statement

10.	please send them (sequence data) to genbank as well		Thank you for your suggestion. We have deposited our all sequences data to Genbank as well with accession number MT324149 to MT324248.
11.	use identifiers particular for your area of study so that they dont get confused with other previously published		Thank you for your suggestion. We used * (asterisk) as identifiers of original haplotype from area of study at notes section below the table.
12.	repeat “find and finding” (Line 290)		Thank you for your correction. We have revised the sentence.
	remove any funding-related text from the manuscript		Thank you for your correction. We have removed any funding-related text.
**No.**	**Reviewers' comments**		**Author Responses**
	**2nd Submission**		
1.	like before: usually 13-16 words,<br>title is too long		Thank you for your correction. We have tried to shorter the title
2.	which will make co-management actions necessary across<br>regions (Line 38-39)		Thank you for your correction in Line 38-39.
3.	I think the introduction too short. According to your title you could write some more about the<br>management used now and what are the points of<br>improvement or change in your eyes for S. lewini		Thank you for your correction. We have tried to enrich the introduction with what this study can give suggestion for better shark management and conservation
	What?<br>..additional.. (Line 177)		Thank you for your correction in Line 177.
	What..? were displayed? That’s not a sentence here (Line 181) in Table		Thank you for your suggestion. We used * (asterisk) as identifiers of original haplotype from area of study at notes section below table.
	One or more? Than add an s		Thank you for your correction. We have revised the sentence.
	One or more? Add s		Thank you for your correction. We have revised the sentence.
	What?? Not a sentence here.. (Line 280)		Thank you for your correction. We have revised the sentence.
	Here smaller font size 11 before 12! check		Thank you for your correction. We have fixed the font size.
	Check fond size here again its smaller!! (Line 290,291,302)		Thank you for your correction. We have fixed the font size.
	One or more? Add s		Thank you for your correction.

	Strange phrase here..I dont know what that should mean..		Thank you for your correction. We have revised the sentence.
**3rd SUBMISSION RESPONSE**			
**No.**	**Additional Editor Comments (if provided)**		**Author Responses**
1.	Thank you for submitting your manuscript to PLOS ONE. After careful consideration, we feel that it has merit but does not fully meet PLOS ONE's publication criteria as it currently stands. Therefore, we invite you to submit a revised version of the manuscript that addresses the points raised during the review process.		
2.	The English still needs further improvement and I think the authors need to further analyze their genetic connectivity section. In the discussion, it does not reflect some of the findings you get, particularly from your haplotype network. Please read my suggestions on the attached PDF.		Thank you for your correction. We have added some sentences in discussion to reflect our genetic data particularly in connectivity section.
3.	We would appreciate receiving your revised manuscript by 15th May 2020. When you are ready to submit your revision, log on to https://www.editorialmanager.com/pone/ and select the 'Submissions Needing Revision' folder to locate your manuscript file.		
4.	If you would like to make changes to your financial disclosure, please include your updated statement in your cover letter.		
5.	To enhance the reproducibility of your results, we recommend that if applicable you deposit your laboratory protocols in protocols.io, where a protocol can be assigned its own identifier (DOI) such that it can be cited independently in the future. For instructions see: http://journals.plos.org/plosone/s/submi		Thank you for your suggestion. We have deposited a short laboratory protocol in protocols.io platform and added the DOI number at the end of Method section, Line 125-127.

	ssion-guidelines#loc-laboratory-protocols<br><br>**Laboratory protocols**<br><br>To enhance the reproducibility of your results, we recommend and encourage you to deposit laboratory protocols in protocols.io, where protocols can be assigned their own persistent digital object identifiers (DOIs).<br><br>To include a link to a protocol in your article:<br><br>1. Describe your step-by-step protocol on protocols.io<br>2. Select **Get DOI** to issue your protocol a persistent digital object identifier (DOI)<br>3. Include the DOI link in the Methods section of your manuscript using the following format provided by protocols.io: http://dx.doi.org/10.17504/protocols.io.[PROTOCOL DOI]<br><br>At this stage, your protocol is only visible to those with the link. This allows editors and reviewers to consult your protocol when evaluating the manuscript. You can make your protocols public at any time by selecting **Publish** on the protocols.io site. Any referenced protocol(s) will automatically be made public when your article is published.		
6.	Please note while forming your response, if your article is accepted, you may have the opportunity to make the peer review history publicly available. The record will include editor decision letters (with reviews) and your responses to reviewer comments. If		Thank you for your suggestion. We have tried to reorganize all comments and our responses in this table to make better history of publication process.

	eligible, we will contact you to opt in or out.		
**No.**	**Reviewers' comments**		**Author Responses**
1.	of a haplotype (typo A)		Thank you for your correction. We have fixed it.
2.	explain how genetic information can be used to trave back the origin of shark find from this species found in markets, particularly in Asia. Cite Fields et al 2020, in Animal Conservation, a manuscript that came out this week.		Thank you for your suggestion. We have added introduction in Line 53-67 and cited from Fields et al and Jabado et al in Line 68-75.
	DNA extraction		Thank you for your correction. We have fixed it.
3.	I dont think you need to inlude this, as this is know by population geneticists and also because it will also depend of the probability of significance.		Thank you for your suggestion. We agreed with your suggestion, we have omitted the sentence.
4.	No significant differentiation was observed<br>(Line 203)		Thank you for your correction. We have revised the sentence.
5.	significant differentiation		Thank you for your correction. We have revised the phrase.
6.	differentiation		Thank you for your correction. We have revised it.
7.	include number of Fst and p value		Thank you for your suggestion. We have added value of Fst and p in the sentence Line 210.
8.	is a highly		Thank you for your correction. We have revised the phrase.
9.	what type of fisheries, be more specific		Thank you for your suggestion. We have added more specific information in Line 275.<br>"according to the global fisheries information system on hammerhead shark capture production 1950-2017 on global scale and Indonesia by FAO"
10.	you have many nucleotide changes between your haplotypes from		Thank you for your correction. We have stated some sentences in Line 318-330

	Indonesia and those from the Western Indian Ocean...what could be happening there? is there really gene flow with the West Indian Ocean? I think gene flow will be restricted, otherwise you would not find such differentiated haplotypes. Re -think this section and go deeper into your results		regarding haplotype differentiation between two haplogroup between Indonesia and western Indian Ocean, and we also highlight in next sentence 331-347, an indication on genetic sharing between population in Aceh and Western Indian Ocean which have similarity in H1 (haplotype one).
11.	and gene flow		Thank you for your correction. We have added the phrase in Line 339.
