# Supplementary material for: Genetic connectivity of the scalloped hammerhead shark Sphyrna lewini across Indonesia and the Western Indian Ocean
Source: PLoS One. 2020 Oct 1;15(10):e0230763. doi: 10.1371/journal.pone.0230763 (PMC7529310; doi:10.1371/journal.pone.0230763)
Supplement: Attachment — Submitted filename: Response to Reviewers.docx [file pone.0230763.s003.docx]

**Dear Susana Caballero, PhD
Academic Editor
PLOS ONE**

Thank you for reviewing and suggesting our manuscript:

“Genetic connectivity and diversity of endangered species the scalloped hammerhead shark Sphyrna lewini (Griffith & Smith 1834) population across Indonesia and the Western Indian Ocean”

I would like to response your comments:

**Additional Editor Comments (if provided):**

This is a very interesting manuscript and it present very important information that will help understanding the genetic diversity of scalloped hammerhead in this region and this information will be likely used for future assignment comparisons for example for market surveys etc. However, the English NEEDS to be improved. I really hope the authors can find the help of a native English speaker that can help them with this. I wrote some things in the PDF I am attaching but it definitely needs an overall improvement of the English

.
**Thank you for your interest and suggestion. We have improved overall of the English for better manuscript after proof read by professional native English speaker.**

The resolution of the figures needs to be improved because they look grainy.

**Thank you for your suggestion. We have improved the resolution of figures and generated in PACE to meet PLOS ONE requirement.**

We would appreciate receiving your revised manuscript by 20th of April. When you are ready to submit your revision, log on to https://www.editorialmanager.com/pone/ and select the 'Submissions Needing Revision' folder to locate your manuscript file.

If you would like to make changes to your financial disclosure, please include your updated statement in your cover letter.

To enhance the reproducibility of your results, we recommend that if applicable you deposit your laboratory protocols in protocols.io, where a protocol can be assigned its own identifier (DOI) such that it can be cited independently in the future. For instructions see: ttp://journals.plos.org/plosone/s/submission-guidelines#loc laboratory-protocols

Please include the following items when submitting your revised manuscript:

- A rebuttal letter that responds to each point raised by the academic editor and reviewer(s). This letter should be uploaded as separate file and labelled **'Response to Reviewers'**.
- A marked-up copy of your manuscript that highlights changes made to the original version. This file should be uploaded as separate file and labelled **'Revised Manuscript with Track Changes'**.
  •An unmarked version of your revised paper without tracked changes. This file should be uploaded as separate file and labelled **'Manuscript'**.

**Journal Requirements:**

When submitting your revision, we need you to address these additional requirements:

1. Please ensure that your manuscript meets PLOS ONE's style requirements, including those for file naming. The PLOS ONE style templates can be found at http://www.plosone.org/attachments/PLOSOne_formatting_sample_main_body.pdf and
   http://www.plosone.org/attachments/PLOSOne_formatting_sample_title_authors_affiliat
   ions.pdf

**Thank you for your suggestion. We have read and followed the format as The PLOS ONE style templates.**

2. In your Methods section, please provide additional location information of the sampling collection sites, including geographic coordinates for the data set if available.

**Thank you for your suggestion. We have provided additional location information including geographic coordinates in a table in manuscript**

3. We suggest you thoroughly copyedit your manuscript for language usage, spelling, and grammar. If you do not know anyone who can help you do this, you may wish to consider employing a professional scientific editing service.

**Thank you for your suggestion. We have sent and proof read our manuscript to a professional scientific editor to check and enhance our manuscript grammar, spelling and language use.**

Upon resubmission, please provide the following:

- The name of the colleague or the details of the professional service that edited your manuscript
- A copy of your manuscript showing your changes by either highlighting them or using track changes (uploaded as a *supporting information* file)
- A clean copy of the edited manuscript (uploaded as the new *manuscript* file)

4. Thank you for stating the following in the Acknowledgments Section of your manuscript:

"This research was supported by Wildlife Conservation Society (WCS)-Indonesia, in collaboration with Faculty of Fisheries and Marine Sciences IPB University. Sutanto Hadi received a scholarship from Indonesia Endowment Fund for Education (LPDP)."

We note that you have provided funding information that is not currently declared in your Funding Statement. However, funding information should not appear in the Acknowledgments section or other areas of your manuscript. We will only publish funding information present in the Funding Statement section of the online submission form.

Please remove any funding-related text from the manuscript and let us know how you would like to update your Funding Statement. Currently, your Funding Statement reads as follows:
"NO - The funders had no role in study design, data collection and analysis, decision to
publish, or preparation of the manuscript."

**Thank you for your suggestion. We have removed any funding-related statement from the
manuscript.**

5. PLOS requires an ORCID iD for the corresponding author in Editorial Manager on papers submitted after December 6th, 2016. Please ensure that you have an ORCID iD and that it is validated in Editorial Manager. To do this, go to ‘Update my Information’ (in the upper left-hand corner of the main menu), and click on the Fetch/Validate link next to the ORCID field. This will take you to the ORCID site and allow you to create a new iD or authenticate a pre-existing iD in Editorial Manager.

Please see the following video for instructions on linking an ORCID iD to your Editorial Manager account:

<https://www.youtube.com/watch?v=_xcclfuvtxQ>

[Note: HTML markup is below. Please do not edit.]

**Reviewers' comments:**

[NOTE: If reviewer comments were submitted as an attachment file, they will be attached to this email and accessible via the submission site. Please log into your account, locate the manuscript record, and check for the action link "View Attachments". If this link does not appear, there are no attachment files to be viewed.]

While revising your submission, please upload your figure files to the Preflight Analysis and Conversion Engine (PACE) digital diagnostic tool, https://pacev2.apexcovantage.com/. PACE helps ensure that figures meet PLOS requirements. To use PACE, you must first register as a user. Registration is free. Then, login and navigate to the UPLOAD tab, where you will find detailed instructions on how to use the tool. If you encounter any issues or have any questions when using PACE, please email us at figures@plos.org. Please note that Supporting Information files do not need this step.

**Thank you for your suggestion. We have uploaded and adjusted the pictures to PACE to meet PLOS ONE figures requirement. We attached the picture in submission.**

Please send them (sequence data) to GenBank as well.

**Thank you for your suggestion. We have deposited our all sequences data to Genbank as well with accession number MT324149 to MT324248.**
