# Supplementary material for: Genetic connectivity of the scalloped hammerhead shark Sphyrna lewini across Indonesia and the Western Indian Ocean
Source: PLoS One. 2020 Oct 1;15(10):e0230763. doi: 10.1371/journal.pone.0230763 (PMC7529310; doi:10.1371/journal.pone.0230763)
Supplement: Attachment — Submitted filename: 1. Response to Reviewers.docx [file pone.0230763.s007.docx]

**To:**

**Susana Caballero, PhD
Academic Editor
PLOS ONE**

Thank you for reviewing and suggesting our manuscript:

“Genetic connectivity and diversity of endangered species the scalloped hammerhead shark Sphyrna lewini (Griffith & Smith 1834) population across Indonesia and the Western Indian Ocean”

I would like to response your comments in the following systematic table format:

| **1^st^ SUBMISSION RESPONSE** | | | |
| --- | --- | --- | --- |
| **No.** | **REVIEWER COMMENT** |  | **AUTHOR RESPONSES** |
|  | **Additional Editor Comments**  **(if provided)** |  |  |
| 1. | This is a very interesting manuscript and it present very important information that will help understanding the genetic diversity of scalloped hammerhead in this region and this information will be likely used for future assignment comparisons for example for market surveys etc. However, the English NEEDS to be improved. I really hope the authors can find the help of a native English speaker that can help them with this. I wrote some things in the PDF I am attaching but it definitely needs an overall improvement of the English |  | Thank you for your interest and suggestion. We have improved overall of the English for better manuscript after proof read by professional native English speaker. |
| 2. | The resolution of the figures needs to be improved because they look grainy |  | Thank you for your suggestion. We have improved the resolution of figures and generated in PACE to meet PLOS ONE requirement. |
| 3. | We would appreciate receiving your revised manuscript by 20th of April. When you are ready to submit your revision, log on to https://www.editorialmanager.com/pone/ and select the 'Submissions Needing Revision' folder to locate your manuscript file.  If you would like to make changes to your financial disclosure, please include your updated statement in your cover letter. |  |  |
| 4. | To enhance the reproducibility of your results, we recommend that if applicable you deposit your laboratory protocols in protocols.io, where a protocol can be assigned its own identifier (DOI) such that it can be cited independently in the future. For instructions see: ttp://journals.plos.org/plosone/s/submission-guidelines#loc laboratory-protocols |  |  |
| 5. | Please include the following items when submitting your revised manuscript:   - A rebuttal letter that responds to each point raised by the academic editor and reviewer(s). This letter should be uploaded as separate file and labelled **'Response to Reviewers'**. - A marked-up copy of your manuscript that highlights changes made to the original version. This file should be uploaded as separate file and labelled **'Revised Manuscript with Track Changes'**. •An unmarked version of your revised paper without tracked changes. This file should be uploaded as separate file and labelled **'Manuscript'**. |  |  |
|  |  |  |  |
| **No.** | **Journal Requirements** |  | **Author Responses** |
| 1. | When submitting your revision, we need you to address these additional requirements:  Please ensure that your manuscript meets PLOS ONE's style requirements, including those for file naming. The PLOS ONE style templates can be found at http://www.plosone.org/attachments/PLOSOne_formatting_sample_main_body.pdf and http://www.plosone.org/attachments/PLOSOne_formatting_sample_title_authors_affiliat ions.pdf |  | Thank you for your suggestion. We have read and followed the format as The PLOS ONE style templates. |
| 2. | In your Methods section, please provide additional location information of the sampling collection sites, including geographic coordinates for the data set if available. |  | Thank you for your suggestion. We have provided additional location information including geographic coordinates in a table in manuscript. |
| 3. | We suggest you thoroughly copyedit your manuscript for language usage, spelling, and grammar. If you do not know anyone who can help you do this, you may wish to consider employing a professional scientific editing service. |  | Thank you for your suggestion. We have sent and proof read our manuscript to a professional scientific editor to check and enhance our manuscript grammar, spelling and language use. |
| 4. | Upon resubmission, please provide the following:   - The name of the colleague or the details of the professional service that edited your manuscript - A copy of your manuscript showing your changes by either highlighting them or using track changes (uploaded as a *supporting information* file) - A clean copy of the edited manuscript (uploaded as the new *manuscript* file) |  |  |
| 5. | Thank you for stating the following in the Acknowledgments Section of your manuscript:  "This research was supported by Wildlife Conservation Society (WCS)-Indonesia, in collaboration with Faculty of Fisheries and Marine Sciences IPB University. Sutanto Hadi received a scholarship from Indonesia Endowment Fund for Education (LPDP)."  We note that you have provided funding information that is not currently declared in your Funding Statement. However, funding information should not appear in the Acknowledgments section or other areas of your manuscript. We will only publish funding information present in the Funding Statement section of the online submission form.  Please remove any funding-related text from the manuscript and let us know how you would like to update your Funding Statement. Currently, your Funding Statement reads as follows: "NO - The funders had no role in study design, data collection and analysis, decision to publish, or preparation of the manuscript." |  | Thank you for your suggestion. We have removed any funding-related statement from the manuscript. |
| 6. | PLOS requires an ORCID iD for the corresponding author in Editorial Manager on papers submitted after December 6th, 2016. Please ensure that you have an ORCID iD and that it is validated in Editorial Manager. To do this, go to ‘Update my Information’ (in the upper left-hand corner of the main menu), and click on the Fetch/Validate link next to the ORCID field. This will take you to the ORCID site and allow you to create a new iD or authenticate a pre-existing iD in Editorial Manager.  Please see the following video for instructions on linking an ORCID iD to your Editorial Manager account:  <https://www.youtube.com/watch?v=_xcclfuvtxQ>  [Note: HTML markup is below. Please do not edit.] |  |  |
| **2^nd^ SUBMISSION RESPONSE** | | | |
| **No.** | **Reviewers' comments** |  | **Author Responses** |
| 1. | While revising your submission, please upload your figure files to the Preflight Analysis and Conversion Engine (PACE) digital diagnostic tool, https://pacev2.apexcovantage.com/. PACE helps ensure that figures meet PLOS requirements. To use PACE, you must first register as a user. Registration is free. Then, login and navigate to the UPLOAD tab, where you will find detailed instructions on how to use the tool. If you encounter any issues or have any questions when using PACE, please email us at figures@plos.org. Please note that Supporting Information files do not need this step. |  | Thank you for your suggestion. We have uploaded and adjusted the pictures to PACE to meet PLOS ONE figures requirement. We attached the picture in submission. |
| 2. | which populations have been declining worldwide |  | Thank you for your correction, we have revised the sentence |
| 3. | use h instead of Hd |  | Thank you for your correction, we have replaced all of term “Hd” with “h” |
| 4. | is considered coastal due to their need for nursery areas |  | Thank you for your suggestion, we used term “semi-oceanic” as stated in some journals which have the similar meaning that you suggested, so we agreed and have revised the sentence |
| 5. | shorter phrases (Line 47-51) |  | Thank you for your correction. We have shortened the phrases. |
| 6. | were already dead when collected |  | Thank you for your correction. We have revised the sentence. |
| 7. | sample (Line 83) |  | Thank you for your correction |
| 8. | 100 samples of which length? |  | Thank you for your question, we used 594 bp of nucleotide and 179 sequences in total, not 100. We have revised it in the sentence |
| 9. | you previously mention a 100 (sequences) |  | Thank you for your correction. We used 179 sequences in total, we have revised in previous statement |
| 10. | please send them (sequence data) to genbank as well |  | Thank you for your suggestion. We have deposited our all sequences data to Genbank as well with accession number MT324149 to MT324248. |
| 11. | use identifiers particular for your area of study so that they dont get confused with other previously published |  | Thank you for your suggestion. We used * (asterisk) as identifiers of original haplotype from area of study at notes section below the table. |
| 12. | repeat “find and finding” (Line 290) |  | Thank you for your correction. We have revised the sentence. |
|  | remove any funding-related text from the manuscript |  | Thank you for your correction. We have removed any funding-related text. |
| **No.** | **Reviewers' comments** |  | **Author Responses** |
|  | **2^nd^ Submission** |  |  |
| 1. | like before: usually 13-16 words, title is too long |  | Thank you for your correction. We have tried to shorter the title |
| 2. | which will make co-management actions necessary across regions (Line 38-39) |  | Thank you for your correction in Line 38-39. |
| 3. | I think the introduction too short. According to your title you could write some more about the management used now and what are the points of improvement or change in your eyes for S. lewini |  | Thank you for your correction. We have tried to enrich the introduction with what this study can give suggestion for better shark management and conservation |
|  | What? ..additional.. (Line 177) |  | Thank you for your correction in Line 177. |
|  | What..? were displayed? That’s not a sentence here (Line 181) in Table |  | Thank you for your suggestion. We used * (asterisk) as identifiers of original haplotype from area of study at notes section below table. |
|  | One or more? Than add an s |  | Thank you for your correction. We have revised the sentence. |
|  | One or more? Add s |  | Thank you for your correction. We have revised the sentence. |
|  | What?? Not a sentence here.. (Line 280) |  | Thank you for your correction. We have revised the sentence. |
|  | Here smaller font size 11 before 12! check |  | Thank you for your correction. We have fixed the font size. |
|  | Check fond size here again its smaller!! (Line 290,291,302) |  | Thank you for your correction. We have fixed the font size. |
|  | One or more? Add s |  | Thank you for your correction. |
|  | Strange phrase here..I dont know what that should mean.. |  | Thank you for your correction. We have revised the sentence. |
| **3^rd^ SUBMISSION RESPONSE** | | | |
| **No.** | **Additional Editor Comments**  **(if provided)** |  | **Author Responses** |
| 1. | Thank you for submitting your manuscript to PLOS ONE. After careful consideration, we feel that it has merit but does not fully meet PLOS ONE’s publication criteria as it currently stands. Therefore, we invite you to submit a revised version of the manuscript that addresses the points raised during the review process. |  |  |
| 2. | The English still needs further improvement and I think the authors need to further analyze their genetic connectivity section.  In the discussion, it does not reflect some of the findings you get, particularly from your haplotype network.  Please read my suggestions on the attached PDF. |  | Thank you for your correction. We have added some sentences in discussion to reflect our genetic data particularly in connectivity section. |
| 3. | We would appreciate receiving your revised manuscript by 15th May 2020. When you are ready to submit your revision, log on to <https://www.editorialmanager.com/pone/> and select the 'Submissions Needing Revision' folder to locate your manuscript file. |  |  |
| 4. | If you would like to make changes to your financial disclosure, please include your updated statement in your cover letter. |  |  |
| 5. | To enhance the reproducibility of your results, we recommend that if applicable you deposit your laboratory protocols in [protocols.io](http://protocols.io), where a protocol can be assigned its own identifier (DOI) such that it can be cited independently in the future. For instructions see: <http://journals.plos.org/plosone/s/submission-guidelines#loc-laboratory-protocols>  Laboratory protocols  To enhance the reproducibility of your results, we recommend and encourage you to deposit laboratory protocols in [protocols.io](https://www.protocols.io/), where protocols can be assigned their own persistent digital object identifiers (DOIs).  To include a link to a protocol in your article:   1. Describe your step-by-step protocol on protocols.io 2. Select **Get DOI** to issue your protocol a persistent digital object identifier (DOI) 3. Include the DOI link in the Methods section of your manuscript using the following format provided by protocols.io: http://dx.doi.org/10.17504/protocols.io.[PROTOCOL DOI]   At this stage, your protocol is only visible to those with the link. This allows editors and reviewers to consult your protocol when evaluating the manuscript. You can make your protocols public at any time by selecting **Publish** on the protocols.io site. Any referenced protocol(s) will automatically be made public when your article is published. |  | Thank you for your suggestion. We have deposited a short laboratory protocol in protocols.io platform and added the DOI number at the end of Method section, Line 125-127. |
| 6. | Please note while forming your response, if your article is accepted, you may have the opportunity to make the peer review history publicly available. The record will include editor decision letters (with reviews) and your responses to reviewer comments. If eligible, we will contact you to opt in or out. |  | Thank you for your suggestion. We have tried to reorganize all comments and our responses in this table to make better history of publication process. |
| **No.** | **Reviewers' comments** |  | **Author Responses** |
| 1. | of a haplotype (typo A) |  | Thank you for your correction. We have fixed it. |
| 2. | explain how genetic information can be used to trave back the origin of shark find from this species found in markets, particularly in Asia. Cite Fields et al 2020, in Animal Conservation, a manuscript that came out this week. |  | Thank you for your suggestion. We have added introduction in Line 53-67 and cited from Fields et al and Jabado et al in Line 68-75. |
|  | DNA extraction |  | Thank you for your correction. We have fixed it. |
| 3. | I dont think you need to inlude this, as this is know by population geneticists and also because it will also depend of the probability of significance. |  | Thank you for your suggestion. We agreed with your suggestion, we have omitted the sentence. |
| 4. | No significant differentiation was observed  (Line 203) |  | Thank you for your correction. We have revised the sentence. |
| 5. | significant differentiation |  | Thank you for your correction. We have revised the phrase. |
| 6. | differentiation |  | Thank you for your correction. We have revised it. |
| 7. | include number of Fst and p value |  | Thank you for your suggestion. We have added value of Fst and p in the sentence Line 210. |
| 8. | is a highly |  | Thank you for your correction. We have revised the phrase. |
| 9. | what type of fisheries, be more specific |  | Thank you for your suggestion. We have added more specific information in Line 275.  “according to the global fisheries information system on hammerhead shark capture production 1950-2017 on global scale and Indonesia by FAO” |
| 10. | you have many nucleotide changes between your haplotypes from Indonesia and those from the Western Indian Ocean...what could be happening there? is there really gene flow with the West Indian Ocean? I think gene flow will be restricted, otherwise you would not find such differentiated haplotypes. Re -think this section and go deeper into your results |  | Thank you for your correction. We have stated some sentences in Line 318-330 regarding haplotype differentiation between two haplogroup between Indonesia and western Indian Ocean, and we also highlight in next sentence 331-347, an indication on genetic sharing between population in Aceh and Western Indian Ocean which have similarity in H1 (haplotype one). |
| 11. | and gene flow |  | Thank you for your correction. We have added the phrase in Line 339. |
| **4^th^ SUBMISSION RESPONSE** | | | |
| **No.** | **Reviewers' comments** |  | **Author Responses** |
| 1. | be careful when you explain your findings. You never talk about insignificant differentiation. You say non-significant and siginificantly different. This mistake was already in the previous version. You can look as example sifferentpapers on genetics to see examples on how to write this. |  | Thank you for your correction.  We have tried to look other paper as a reference to fix it. |
| 2. | in a network you can say haplotype sharing but be careful when you say gene flow...haplotype sharing can be the result of gene flow OR from ancestra sharing of alleles. |  | Thank you for your suggestions.  We have detected haplotype sharing between population in Aceh and the Western Indian Ocean, but as you said maybe it is not as a result of gene flow. We have added some sentences to reflected our result in abstract and discussion in Genetic Connectivity section. |
| 3. | The combination (Line 65) |  | Thank you for your correction. We have fix it. |
| 4. | protocols (plural) (Line 125) |  | Thank you for your correction. We have fixed it. |
| 5. | what about the probability? was it significant or non-significant?  (Line 214) |  | Thank you for your question. It was significant, we have written the value of Fst and P. |
